# NIR-II nanoprobes in-vivo assembly to improve image-guided surgery for metastatic ovarian cancer

**DOI:** 10.1038/s41467-018-05113-8

**Published:** 2018-07-24

**Authors:** Peiyuan Wang, Yong Fan, Lingfei Lu, Lu Liu, Lingling Fan, Mengyao Zhao, Yang Xie, Congjian Xu, Fan Zhang

**Affiliations:** 1 Department of Chemistry, Shanghai Key Laboratory of Molecular Catalysis and Innovative Materials, State Key Laboratory of Molecular Engineering of Polymers and iChem, Fudan University, Shanghai, 200433 P.R. China; 2 Department of Obstetrics and Gynecology of Shanghai Medical School, Fudan University, Shanghai, 200032 P.R. China; 3 Obstetrics and Gynecology Hospital, Fudan University, Shanghai, 200011 P.R. China; 4 Department of Orthopedics, Changhai Hospital, Second Military Medical University, Shanghai, 200433 P.R. China

## Abstract

Local recurrence is a common cause of treatment failure for patients with solid tumors. Tumor-specific intraoperative fluorescence imaging may improve staging and debulking efforts in cytoreductive surgery and, thereby improve prognosis. Here, we report in vivo assembly of the second near-infrared window (NIR-II) emitting downconversion nanoparticles (DCNPs) modified with DNA and targeting peptides to improve the image-guided surgery for metastatic ovarian cancer. The NIR-II imaging quality with DCNPs is superior to that of clinically approved ICG with good photostability and deep tissue penetration (8 mm). Stable tumor retention period experienced 6 h by in vivo assembly of nanoprobes can be used for precise tumor resection. Superior tumor-to-normal tissue ratio is successfully achieved to facilitate the abdominal ovarian metastases surgical delineation. Metastases with ≤1 mm can be completely excised under NIR-II bioimaging guidance. This novel technology provides a general new basis for the future design of nanomaterials for medical applications.

## Abstract (web summary)

Real-time fluorescence imaging in the NIR-II region offers non-invasive detection of ovarian metastatic tumors and their image-guided surgery. Here the authors describe NIR-II nanoprobes in vivo assembly for detection of disseminated ovarian cancer.

## Introduction

Surgical removal of malignant disease constitutes one of the most common and effective treatments for cancer and is often the only curative treatment option^1–3^. The ability to visualize the full extent of tumor during the operation, including regional metastatic spread and microscopic lesions, has major implications for the therapeutic outcome^4–6^. However, surgeons can mainly rely on palpation and visual inspection currently. Highly sensitive intraoperative detection of small and occult tumors remains a challenge for conventional imaging modalities, such as X-ray, computed tomography (CT), magnetic resonance imaging (MRI), and ultrasound with limited sensitivity and signal specificity, long acquisition time, and ionizing radiation risk^7–11^. In contrast, in vivo fluorescence imaging has emerged as a valuable tool for improving diagnosis of staging tumors, monitoring response to therapy, and detecting recurrent or residual disease^12–15^. Fluorescence imaging offers the promise of safe, noninvasive detection with key advantages including real-time, superior resolution, and high specificity for small tumor nodules during diagnostic and intraoperative surgical procedures^16–19^. Recently, efforts have focused on using visible and short near-infrared (NIR-I, 650–900 nm) wavelength fluorescent dyes as contrast agents for delineating tumor margins in both preclinical cancer models and human patients. However, these agents are suboptimal for reflectance-based intraoperative imaging due to limited penetration depth (1–3 mm) and high-tissue autofluorescence^20–22^. Second-window near-infrared fluorescence (NIR-II, 900–1700 nm) probes such as single-walled carbon nanotubes (SWNTs)^23,24^, quantum dots (QDs)^25,26^, lanthanide-based downconversion nanoparticles (DCNPs)^27–29^, and organic dyes^30–33^ are promising for in vivo fluorescence imaging due to sub-10-µm high-resolution imaging at a few centimeters tissue penetration depth and low-tissue autofluorescence^23–33^, which are promising candidates for both preoperative imaging and intraoperative reality^28^. Especially, DCNPs play a very important role in the NIR-II fluorescent bioimaging applications due to their distinct properties, such as highly controlled particle size, nonphotobleaching, long lifetime and high-efficiency optical properties^27–29^.

Furthermore, in current fluorescence image-guided surgery practice, long tumor retention period with photostable probes is essential for the following precision imaging-guided resection. Although fluorescence imaging and surgical guidance of tumors with clinically approved indocyanine green (ICG)^34–36^ and methylene blue (MB)^37,38^ have been widely investigated to detect a variety of tumors. However, these probes typically experienced short-time tumor retention because of their rapid clearance. On the other hand, fluorescent probes for surgical navigation often severely accumulate in organs of the reticuloendothelial system (RES, such as the liver and spleen), and induce contamination for the intestinal tract, which will increase the unwanted background signals to interfere with the image-guided surgery of abdominal tumor^39–42^. Therefore, the effective image-guided surgery strategy with high tumor-to-normal tissue (T/N) ratio and long tumor retention are prerequisite to intraoperatively visualize the contrast between tumor nidus and normal tissue in real time.

Herein, we report in vivo assembly of NIR-II emitting DCNPs modified with DNA and targeting peptides to improve the image-guided surgery for metastatic ovarian cancer. The stable tumor retention period experienced as long as 6 h by organizing the tumor targeted DCNP building blocks into larger assembled superstructures. In addition, we found that NIR-II fluorescence bioimaging of in vivo assembled nanoprobes can accurately delineate tumors margins and tumors, which were capable of precisely being removed during this long and stable tumor retention window. Furthermore, RES retention was reduced accompanied with refraining from the assembly of the building blocks in bloodstream by two-staged in sequence injection of the building blocks for the tumor site in vivo assembly. This approach combines the concerns about chronic toxicity and whole body elimination, resulting in weak background signals. Therefore, T/N ratio was significantly enhanced to facilitate the abdominal ovarian metastases surgical delineation. Histology analysis of hematoxylin and eosin (H&E) staining confirms that metastases with ≤1 mm can be completely excised under NIR-II fluorescence bioimaging guidance. These findings of NIR-II fluorescence image-guided tumor surgery via in vivo assembly hold promise for effective clinical application.

## Results

### Preparation and in vitro assembly of NIR-II Nanoprobes

**Fig. 1:**
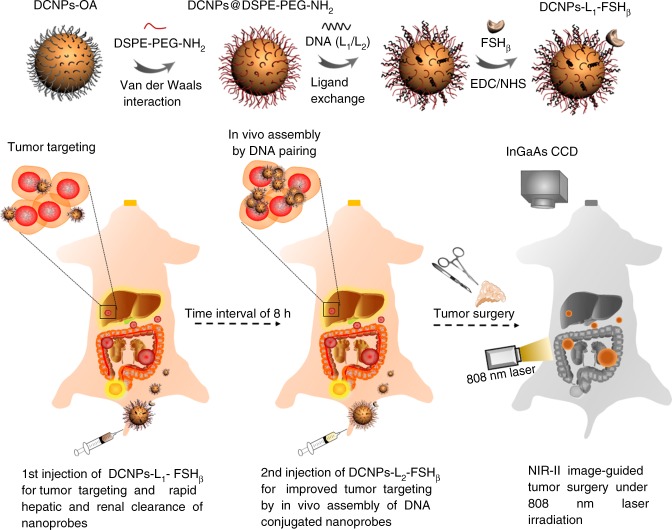
Schematic illustration of NIR-II nanoprobes fabrication for ovarian metastasis surgery under NIR-II bioimaging guidance. Schematic of stepwise fabrication of DNA and FSH_β_ modified DCNPs (DCNPs-L_1_-FSH_β_) and in vivo assembly of DCNPs-L_1_-FSH_β_ (first injection) and DCNPs-L_2_-FSH_β_ (second injection) with improved tumor targeting and rapid hepatic and renal clearance after two-staged in sequence injections. Metastatic ovarian tumors can be clearly observed and precisely removed with NIR-II image-guided surgery

**Fig. 2:**
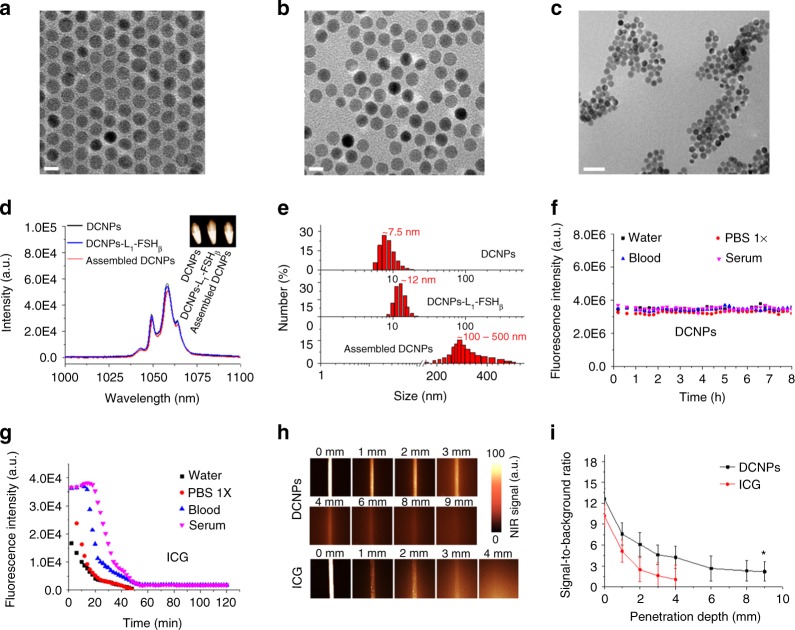
In vitro assembly of NIR-II nanoprobes. Transmission electron microscope (TEM) images of as-made NaGdF_4_: 5% Nd@NaGdF_4_ nanocrystals (**a**), DNA (L_1_) modified DCNPs (**b**) and DNA complementary induced assembly between DCNPs-L_1_-FSH_β_ and DCNPs-L_2_-FSH_β_ in PBS (**c**). **d** The NIR-II fluorescence spectrum of DCNPs, DCNPs-L_1_-FSH_β_, and self-assembled DCNPs. Inset, NIR-II fluorescence images of the corresponding samples. **e** Size distribution of DCNPs, DCNPs-L_1_-FSH_β_, and self-assembled DCNPs determined by dynamic light scattering. **f**, **g** Photostability of DCNPs (**f**) and ICG (**g**) in a variety of biological media at 37 °C under continuous 808 nm laser exposure at a power density of 0.2 W cm^−2^. **h** NIR-II fluorescence images show complete attenuation of NIR-I light (ICG) by 3 mm, while NIR-II signals (DCNPs) are able to be detected through 8 mm of phantom tissues. Representative images are for *n* = 5 per group. **i** Signal to background ratios of DCNPs and ICG as a function of tissue phantom depth. Scale bars represent 10 nm in **a**, **b** and 50 nm in (**c**). Mean ± s.d. for *n* = 5 (*P < 0.05 vs. ICG, two-sided Student’s *t* test)

Figure 1 illustrates the principles of NIR-II image-guided ovarian tumor resection based on in vivo self-assembly of DNA functionalized NIR-II lanthanide probes for improved tumor targeting by two-staged in sequence injection. The NIR-II fluorescence NaGdF_4_: 5% Nd@NaGdF_4_ DCNPs were fabricated by the successive layer-by-layer (SILAR) method with uniform particle size of ~7.5 nm and highly efficient 1060 nm NIR-II emission under 808 nm laser irradiation^27^ (Figs. 1 and 2a, d, e). To facilitate the bioapplication, the oleic acid capped DCNPs were transferred to the aqueous phase by using monolayer of amine-phospholipids through Van der waals interaction^43^. The fluorescence quenching effect of water can be avoided after gradually coating a 2.5 nm inert NaGdF_4_ shell on the 5.0 nm luminescent NaGdF_4_: 5% Nd cores (Supplementary Fig. 1). Then direct surface conjugation of DCNPs with complementary DNA (L_1_ or L_2_) was realized through ligand exchange strategy^44^ (Figs. 1 and 2b, d). In order to improve the ovarian tumor targeting efficiency, follicle-stimulating hormone (FSH_β_) peptide specific to the epithelium ovarian cancer^45^ (Supplementary Fig. 2) was covalently anchored on DCNPs via an EDC/NHS reaction to obtain DCNPs-L_1_-FSH_β_ nanoprobes. These nanoprobes possessed superior aqueous solubility, stability, and sustainable emission fluorescence in water and different biological buffers at 37 °C (Fig. 2f, Supplementary Figs. 3−5). We subsequently explored the strategies for fabricating DNA-based assemblies. The size distribution of the DCNPs-L_1_-FSH_β_ is ~8–17 nm. However, after introducing the complementary probe DCNPs-L_2_-FSH_β_ in biological buffers (phosphate buffer solution (PBS) 1×), the size distribution was changed to ~100–500 nm, clearly suggesting that DCNPs grafted with specific DNA sequence can assemble efficiently (Fig. 2c, d, e). Due to the huge potentials of complementary nature of DNA hybridization, the observed hydrodynamic diameters of nanoclusters in serum at 37 °C were stable over long periods of study (~8 h) under a continuous wave of 808 nm laser irradiation (laser output power density = 0.2 W cm^−2^, fluence rate 40 mW cm^−2^, and working distance = 30 cm^46^) (Supplementary Figs. 6 and 7), suggesting the assembled DCNPs were very stable in tumor site during the bioimaging process. Moreover, compared with the clinically approved ICG probe, superior photostability of DCNPs were observed by exposing the nanoprobes in water, PBS, blood, and serum at 37 °C to a continuous irradiation with 808 nm laser (Fig. 2f, g; Supplementary Fig. 8). The penetration depth of DCNPs (8 mm) was almost three times higher than ICG (3 mm) and the signal to background ratio of DCNPs (~4.7) was also threefold higher than ICG (~1.6) in 3 mm depth (Fig. 2h, i; Supplementary Fig. 9), indicating the advantage of the DCNPs nanoprobes for bioimaging. Before the in vivo ovarian tumor bioimaging study, the potential cytotoxicity of DNA and FSH_β_ modified DCNPs were evaluated in human ovarian carcinoma cell line CaOV_3_. The cells exhibited over 85% viability after incubation with 500 μg mL^−1^ nanoprobes, indicating the low cytotoxicity of intracellular assembly (Supplementary Fig. 10). Moreover, creatinine and glutamic pyruvic transaminase levels in the mice blood, as the indicators of kidney and liver function, are kept normal after injection of NIR-II nanoprobes, demonstrating little side effect of these nanoprobes on kidney and liver (Supplementary Fig. 11).

### In vivo assembly of NIR-II nanoprobes

**Fig. 3:**
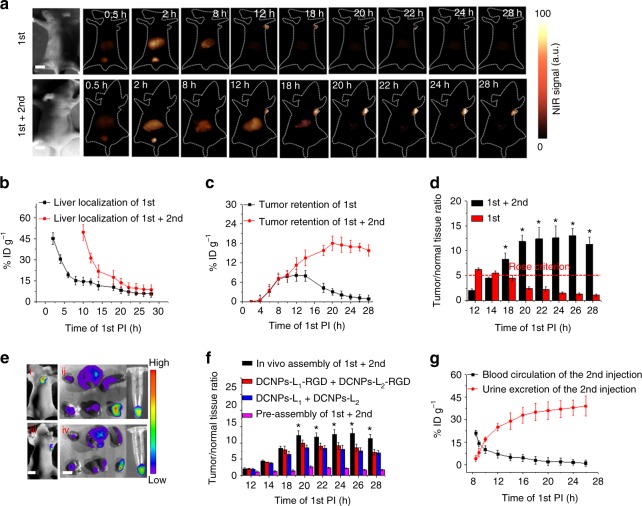
In vivo assembly of NIR-II nanoprobes. **a** NIR-II fluorescence bioimaging (1000 nm long-pass filter) of the nude mice with murine epidermal tumor by single caudal vein first injection and two-staged in sequence injection (first + second) (interval between two injection is 8 h) under 808 nm excitation (fluence rate = 40 mW cm^−2^). The concentration of DCNPs in single injection is same to the sum of that for two-staged injection. Liver distribution of nanoprobes (**b**), tumor targeting efficiency (**c**), and T/N ratio (**d**) with single first injection and two-staged in sequence injection, respectively. The red dotted line in **d** indicates the Rose criterion. (^*^P < 0.05 vs. first, two-sided Student’s *t* test). **e** Fluorescence images of DCNPs-L_1_(Cy5)-FSH_β_ + DCNPs-L_2_(Cy7)-FSH_β_ (i) and DCNPs-L_1_(Cy5)-FSH_β_ + DCNPs-L_1_(Cy7)-FSH_β_ (ii) in epidermal tumor of the nude mice and the corresponding harvest organs, blood and tumors (left to right and to bottom: heart, liver, spleen, lung, kidney, tumor, and blood). **f** T/N ratio of the in vivo assembly (first + second), active targeting (DCNPs-L_1_-RGD + DCNPs-L_2_-RGD), passive targeting (DCNPs-L_1_ + DCNPs-L_2_), and preassembly (first + second) groups. (^*^P < 0.05 vs. DCNPs-L_1_-RGD + DCNP-L_2_-RGD, two-sided Student’s *t* test). **g** Blood circulation and urine excretion of nanoprobes after second injection. The T/N ratio is tumor-to-liver ratio in this epidermal tumor model experiments. All scale bars, 1 cm. Representative images are for *n* = 5 per group. Mean ± s.d. for *n* = 5

Encouraged by the above in vitro assembly results, the in vivo experiment was carried out in a murine model with subcutaneous human ovarian adenocarcinoma. After tail injection of DCNPs-L_1_-FSH_β_, NIR-II fluorescence in both liver and bladder can be clearly observed in 30 min and showed a minimum signal in liver at approximately 8 h postinjection (PI), suggesting the hepatic and renal clearance of the nanoprobes (Fig. 3a). Furthermore, the NIR-II fluorescent signals can be observed within tumor at 6 h PI and reach at a maximum at 12 h PI. Then the fluorescence in tumor site gradually faded from 14 h PI and markedly decreased from 18 h PI. The fluorescence signals of blood samples, collected from 2 to 18 h PI, were greatly reduced at 8 h PI and all that of RES organs were also very weak from then on (Supplementary Figs. 12 and 13). Moreover, according to biodistribution results, the half-life of the nanoprobes in blood is ~2 h and 45.1% ID g^−1^ is excreted from urine (Supplementary Fig. 14a). Meanwhile, the biodistribution of nanoprobes in RES organs and tumors were investigated by inductively coupled plasma mass spectrometer (ICP-MS). The liver retention of nanoprobes decreased markedly from 45.2% to 15.1% ID g^−1^ in 8 h PI, and then decreased to 5.6% ID g^−1^ at 28 h PI. The maximum tumor retention of nanoprobes was only 8.1% ID g^−1^ (at 12 h PI) and then continuously decreased to below 3.1% after 20 h PI, demonstrating that the nanoprobes typically possess insufficient tumor retention because of their rapid clearance (Fig. 3b, c; Supplementary Fig. 14b). The rapid whole-body elimination and short time tumor retention of the nanoprobes resulted in continuously attenuated T/N ratio (from 5.5 at 12 h PI to 2.5 at 20 h PI) (Fig. 3d). According to the Rose criterion, which states that a T/N ratio of 5 is needed to distinguish image features with 100% certainty^30^, therefore single injection of DCNPs-L_1_-FSH_β_ is unfavorable for stable image-guided tumor surgery.

We were then motivated to study whether in vivo assembly could alter the tumor retention kinetics of NIR-II nanoprobes in a favorable manner. To validate the simultaneous assembly performance of the two building blocks in vivo, we tested whether nanoprobes grafted with Cy5 fluorescent dyes labeled DNA (DCNPs-L_1_(Cy5)-FSH_β_) could spontaneously bind nanoprobes linked with Cy7 labeled complementary DNA (DCNPs-L_2_(Cy7)-FSH_β_), causing Förster resonance energy transfer (FRET) between the two dyes while bringing them within close proximity. The FRET-specific fluorescence for both tumor and harvest organs of the complementary group were more than twice as high as the noncomplementary group (DCNPs-L_1_(Cy5)-FSH_β_ + DCNPs-L_1_(Cy7)-FSH_β_) (Fig. 3e; Supplementary Fig. 15), demonstrating the colocalization assembly of the two complementary components can occur within tumors. Meanwhile, as shown in the confocal laser scanning microscope results of tumor frozen section (Supplementary Fig. 16), FRET signals can be observed around the nuclei of tumor cells and few signals can be detected in interstitial space. Furthermore, in vivo assembled DCNPs in tumor site can also be clearly observed by transmission electron microscope (TEM) images^47^ (Supplementary Fig. 17).

In order to achieve both efficient tumor retention and rapid RES clearance, the interval between two injections of DCNPs-L_1_-FSH_β_ (first) and complementary nanoprobes DCNPs-L_2_ -FSH_β_ (second) was optimized. The superior T/N ratio was realized when the second was administrated at 8 h PI of first injection (Supplementary Figs. 18–22). The NIR-II fluorescence in tumor site gradually increased from 18 h PI of first injection and significantly enhanced after 20 h PI of first injection (Fig. 3a). The T/N ratio from 20 h to 28 h PI of first injection with the two-staged in sequence injection strategy was maintained at ~12.5 steadily and more than five times higher than that of single injection approach with only DCNPs-L_1_-FSH_β_ (T/N = 2.5 in 20 h PI) (Fig. 3d). Furthermore, compared with nonassembling (first + first_,_ T/N = 2.5) and preassembly (first + second, T/N = 2.7) groups, the in vivo assembly strategy exhibited highest T/N ratio (Fig. 3f; Supplementary Figs. 23–25). In comparison to the passive targeting nanoprobes (DCNPs-L_1_ + DCNPs-L_2,_ T/N = 8.6) and arginine–glycine–aspartic acid (RGD) targeting motifs modified nanoprobes (T/N = 9.9), FSH_β_ modification shows the most efficient targeting performance for this in vivo assembly strategy (Supplementary Fig. 25).

We then explored the tumor retention efficiency of the two-staged in sequence injection strategy by ICP-MS. Interestingly, the maximum tumor retention amount of two-staged injection of complementary NIR-II nanoprobes (~17.5% ID g^−1^) were two-fold higher than single injection of DCNPs-L_1_-FSH_β_ (~8.1% ID g^−1^) (Fig. 3b) with same amount, clearly demonstrating the advantage of this novel strategy with improved tumor retention. Moreover, we also assessed the potential of body excretion properties after the second injection. The half-life of the nanoprobes in blood circulation was found to be approximately 2 h when the second was administrated at 8 h PI of first injection (Fig. 3g), which was similar to that of single first injection (Supplementary Fig. 14a), and increased to ~6 h and ~10 h when the second injection was administrated at 6 h PI and 4 h PI of first injection (Supplementary Fig. 26), demonstrating that the in sequence injection strategy with 8 h intervals can effectively prevent assembling of complementary probes in blood. After 2 h PI of second injection, the remarkable NIR-II fluorescent signal was observed in the bladder (Supplementary Fig. 27), suggesting part of nanoprobes could also transport down the ureters bilaterally and into the bladder for renal excretion. The excretion kinetics was investigated by intravenously injecting the two components in sequence, collecting urine and blood over the course of 26 h PI of first injection for ICP-MS analysis. In all, ~39.5% ID g^−1^ of the imaging agent was excreted through the urine (Fig. 3g), which was coincided with urine excretion (~45.1% ID g^−1^) results with only first injection (Supplementary Fig. 14a). Furthermore, the NIR-II fluorescence signal of the blood samples is diminished at 10 h PI of second injection, which is almost consistent with the first injection (8 h PI), further suggesting that additional second injection has little effect on body clearance (Supplementary Figs. 12, 28 and 29). More importantly, as the main RES, the biodistribution of nanoprobes in liver were greatly reduced at 10 h PI of second injection, which is also agree with liver localization with only first injection (8 h PI) (Fig. 3b). All these results demonstrated the validity of our in sequence injection strategy to achieve effective both tumor retention and body clearance.

### NIR-II image-guided metastatic tumor lesions surgery

**Fig. 4:**
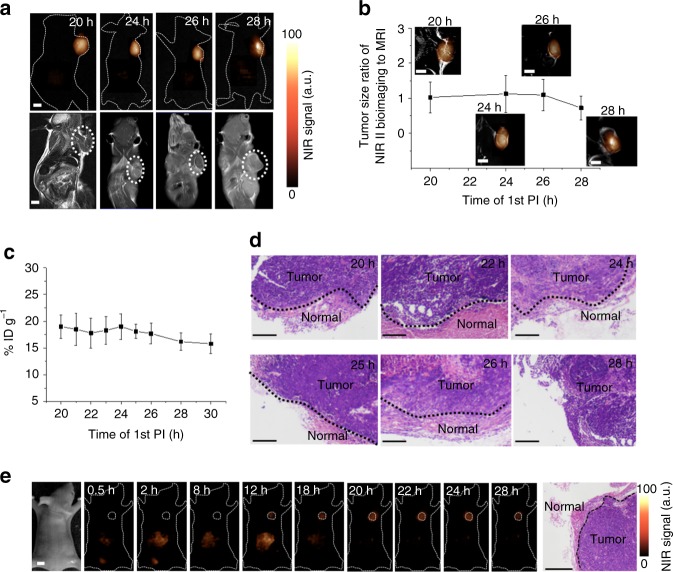
Optimal surgery time window of in vivo assembly. **a** Comparison of MRI and NIR-II fluorescence imaging (1000 nm long-pass filter) of subcutaneous human ovarian adenocarcinoma. **b** Correlation for the tumor size ratio between MRI and NIR-II fluorescence bioimaging method. **c** Tumor retention of assembled DCNPs from 20 to 30 h PI. **d** H&E staining results of the tumors resected in 20–28 h PI under NIR-II fluorescence bioimaging guidance. **e** NIR-II fluorescence image-guided tumor surgery with the guided dose of clinic approved ICG probe (1.5 mg kg^−1^). Tumor margin was confirmed by the H&E staining result. Scale bars, 5 mm in NIR-II bioimaging and MRI results, 0.2 mm in H&E staining images. Representative images are for *n* = 5 per group. Mean ± s.d. for *n* = 5

In order to realize precise image-guided resection for the tumor, it is a key to make sure the full outline of tumors with various size can be visualized during the surgery process. The T/N ratio with the in vivo assembly strategy has been demonstrated can be kept at ~12.5 constantly from 20 to 28 h PI of first injection, which was 2.5-folds and 2-folds higher than that of reported NIR-I probes ICG (~5)^34^ and NIR-II emission carbon nanotube (~6)^16^, respectively, allowing accurate image-guided tumor-removal surgery. The full outline of tumors with various sizes can be visualized by the NIR-II bioimaging in this superior time window (Supplementary Figs. 30 and 31). Tumor delineating effect of NIR-II fluorescence bioimaging via in vivo assembly was further evaluated by using MRI and histopathological analyses. MRI was carried out with the clinical contrast agent of gadobenate dimeglumine (Gd-BOPTA). As shown in Fig. 4a, the tumors margin can be readily distinguished by MRI. The tumor profile detected by the NIR-II imaging exhibited excellent consistency with that of MRI results from 20 to 26 h PI of first injection (Fig. 4b), suggesting that there was a ~6 h stable “optimal surgical time window” for ovarian tumor resection. The nanoprobes accumulated in tumor were investigated by the ICP-MS, revealing stable retention in tumor from 20 to 26 h PI of first injection (Fig. 4c). On the other hand, tumors were removed in this surgical time window (Supplementary Movie 1; Supplementary Fig. 32) and then evaluated by H&E staining. The border regions between normal and tumor tissues indicated the efficient tumor margin identification in the surgical time window (Fig. 4d). Meanwhile, the resected tumor in 28 h PI has no borderline between the tumor and normal tissues in comparison to those operated in 20–26 h PI (Fig. 4d; Supplementary Fig. 33). Then, the peripheral tissue was further collected and the H&E staining demonstrated that there was residual tumor with resection beyond the optimal surgical time window (Supplementary Fig. 34). According to H&E results, even the eye-invisible ultrasmall tumors ≤1 mm (eight days after CaVO_3_ cells subcutaneous injection) can be detected and completely resected in this surgical time window (Supplementary Fig. 35). Finally, it is worth mentioning that NIR-II fluorescence image-guided surgery was carried out with the clinical approved dose (1.5 mg kg^−1^)^48^, and fluence rate (40 mW cm^−2^)^49^ (Fig. 4e; Supplementary Figs. 36–38). Tumor can be successfully removed under NIR-II bioimaging guidance, suggesting potential clinical application of our in vivo assembly strategy. Furthermore, other two ovarian epidermal tumor models (HO8910 and A2780 cell lines) with lower FSH_β_ receptor (FSHR) expression levels can also be observed and thoroughly removed, suggesting the universality of our approach for ovarian tumor surgery (Supplementary Figs. 2, 39, 40, and 41).

**Fig. 5:**
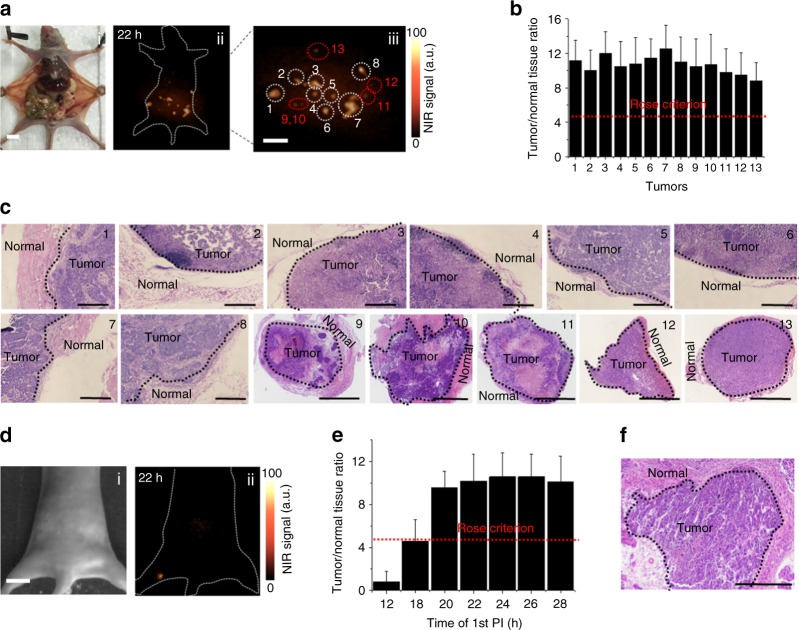
NIR-II image-guided surgery. **a** Optical photo of human ovarian adenocarcinoma peritoneal metastases model (i), the corresponding NIR-II fluorescence bioimaging (1000 nm long-pass filter) results obtained at 22 h PI (ii) and the enlargement of the NIR-II nanoprobes labeled large peritoneal metastatic tumors (Nos. 1–8) and ultrasmall lesions (Nos. 9–13) (iii). Scale bar, 1 cm. **b** T/N ratios plotted as a function of different labeled peritoneal metastatic tumors, red dotted line is according to the Rose criterion. **c** H&E staining results of tumor margin in Nos. 1–8 (scale bars, 0.2 mm) and metastatic lesions in Nos. 9–13 (scale bars, 0.5 mm). Tumors were resected under NIR-II fluorescence bioimaging guidance in **a**. **d** NIR-II fluorescence bioimaging results of the popliteal lymph node metastasis at 22 h PI. Scale bar, 1 cm. **e** T/N ratios plotted as a function of different PI of the first injection, red dotted line is according to the Rose criterion. **f** H&E staining results of popliteal lymph node metastasis (Scale bar, 0.5 mm). Tumors were resected under NIR-II fluorescence bioimaging guidance in **d**. The T/N ratio is tumor-to-normal peripheral tissue ratio in this peritoneal metastases model and popliteal lymph node metastasis model experiments. Representative images are for *n* = 5 per model. Mean ± s.d. for *n* = 5

We finally investigated the potential application for intraoperative imaging of this in vivo assembly strategy in human ovarian adenocarcinoma peritoneal metastases model for the first time (Supplementary Figs. 42–44). Unexpectedly, all the large tumor boundary (Nos. 1–8) and invisible small metastatic lesion (Nos. 9–13) are capable of being identified by NIR-II fluorescence bioimaging in the optimal surgical time window (Fig. 5a; Supplementary Movie 2 and Supplementary Fig. 45). T/N ratios of all the tumors were still kept on the ~11 similar to that of the epidermal tumor (Fig. 5b). H&E staining were studied after tumors were removed under NIR-II fluorescence bioimaging, confirming the precise delineation of the tumor margin (Fig. 5c, Nos. 1–8). Significantly, ≤1 mm metastatic lesions were thoroughly removed, further demonstrating that the novel tumor targeting strategy were able to correctly identify eye-invisible cancerous metastases (Fig. 5c, Nos. 9–13). Finally, this in vivo strategy was further applied for early lesions diagnosis with all T/N ratios are higher than 9.0 (Supplementary Figs. 46 and 47). H&E staining further illustrated the subsequent effective surgical resection with size ≤1 mm (Supplementary Fig. 48), and all the resected fluorescent metastases were confirmed to be malignant. Furthermore, for additional resection of lymph node metastasis^50^, the in vivo assembly strategy can further facilitate popliteal lymph node metastasis detection (Fig. 5d, e; Supplementary Figs. 49–53). H&E staining result demonstrated that microscopic lymph node metastasis can be successfully removed (Fig. 5f).

## Discussion

Imaging-guided radiologic surgical approaches such as X-ray, CT imaging, MRI, and ultrasound had a considerable role in preoperative staging and intraoperative planning of resection, especially in the field of neurosurgical image guidance^6,7,30^. They have been used in assisting surgical procedures, which can observe the entire human body with almost unlimited depth in soft tissues^43^. However, these imaging platform set-ups are all complex imaging systems equipped with either a high-voltage, X-ray source or powerful magnetic fields, which can cost millions of dollars to build^51^. Most important of all, these approaches are not tumor specific and generally are not useful for intraoperative applications^17^. In contrast, fluorescence imaging, as an optical technique, relates naturally to surgical inspection and practice, offers superior resolution and sensitivity compared to preoperative radiological imaging and to visual inspection and palpation during surgery^16–19,52^.

Paradigm shifts in surgery arise when surgeons are empowered to perform surgery faster, better, and less expensively than current standards. Optical imaging that exploits invisible NIR-I fluorescent light has the potential to improve cancer surgery outcomes, minimize the time patients are under anesthesia and lower healthcare costs largely by way of its improved contrast and depth of tissue penetration relative to visible light^53,54^. Accordingly, the past few years have witnessed an explosion of proof-of-concept clinical trials in the field. Although lots of image contrasts (such as clinically approved ICG^34–36^ and MB^37,38^ dyes) have been widely investigated for surgical resection, however, the penetration depth, massive RES uptake and unstable tumor retention induced unreliable surgical time window, thereby reducing the ability of the surgeon to readily locate and resect tumors.

The contrast agent emitting within the NIR-II light has diminished atuofluorescence and allows centimeters imaging depth at low resolution and microscale resolution of anatomical feature that are otherwise unresolvable within the traditional NIR-I region. Thus far, NIR-II contrast agents including SWNTs^23,24^, QDs^25,26^, DCNPs^27–29^, and organic dyes^30–33^ have been established for in vivo imaging of vascular flow, lymphatic, and tumors. Although the organic dyes shows great potentials to facilitate Food and Drug Administration approval and clinical translation, these small molecule probes typically experienced short time tumor retention because of their rapid clearance, which hindered the following precision imaging guided resection. For the inorganic SWNTs, QDs, and DCNPs NIR-II probes, the long-term cytotoxicity is still a major concern. One way to overcome these problems is to design nanoprobes with sizes smaller than 10 nm, i.e., below what is believed to be the threshold for renal clearance^55,56^. Since the well controllable particle size and superior optical properties, including nonphotobleaching, long lifetime and high efficiency, the DCNPs NIR-II probes have attracted a great of attentions for the in vivo bioimaging recently. For example, early ovarian tumor detection was explored by using lanthanide DCNPs (Er^3+^ and Ho^3+^ doped)^39,57^, and even multiorgan cancer metastases could be successfully visualized (Er^3+^ doped)^58^. Unfortunately, all current in vivo bioimaging strategy for imaging-guided surgery with DCNPs NIR-II nanoprobes are still limited by the serious RES accumulation and short-time tumor retention^28,29^. In the present work, we have developed a novel in vivo self-assembly bioimaging strategy to realize the rapid whole body clearance and long tumor retention nanoprobes and then utilized the NIR-II fluorescence bioimaging for efficient tumor surgery. The in vivo assembly strategy has two outstanding advantages. First, the NIR-II fluorescent nanoprobes showed ~8 mm penetration depth. With two-staged in sequence injection strategy, the long tumor retention (~6 h) of the assembled contrast agents can serve as the “optimal surgical time window” for sufficient ovarian surgery. Meanwhile, RES organs and blood are exempted from nanoprobes assembling. Second, in comparison with the clinically approved ICG probes (Supplementary Fig. 54), T/N ratio was significantly enhanced (~12.5) with background signals reduced in this special time window, resulting in the discrimination between malignant and normal tissue types which avoid incomplete resections or the unnecessary removal of healthy tissue. Inspired by these two specialties, peritoneal ovarian metastasis are accurately observed and precisely resected for the first time. Experienced ovarian cancer surgeons can already remove all visible disease through extensive surgical effort, and yet over 80% of women with advanced high grade serous carcinoma still relapse. The recent clinical study indicated that the additional resection of macroscopically normal nodes in addition to all visible macroscopic disease made no impact upon progression/recurrence. In the present work, since ≤1 mm metastatic lesions can be thoroughly removed, this novel tumor targeting strategy might be able to avoid the recurrence by correctly identifying eye-invisible cancerous metastases.

In summary, our results present the potential benefit of intraoperative tumor-specific NIR-II fluorescence imaging in staging and debulking surgery for ovarian cancer using the nanoprobes in vivo assembly strategy. This technique did not create unwanted interference with standard surgical procedures. The combination of optical imaging technologies with tumor-targeting strategies can shift the paradigm of surgical oncologic imaging, offering the unique opportunity to intraoperatively detect and quantify tumor growth and intra-abdominal spread. The results presented here clearly demonstrate the concept and advantage of in vivo assembly strategy facilitating for other abdominal metastases, such as liver tumor, intraoperative delineation, and surgery.

## Methods

### Nanoprobes synthesis

NIR-II fluorescence NaGdF_4_: 5% Nd@NaGdF_4_ DCNPs were fabricated by the SILAR method^27^. Please see the supporting information for the details for the NaGdF_4_: 5% Nd@NaGdF_4_ DCNPs nanoparticle synthesis protocols.

### DNA and FSH_β_ peptide modified DCNPs nanoprobes preparation

The oleic acid capped DCNPs (0.1 mmol) in 5 mL of chloroform was mixed with a chloroform solution (1 mL) containing 25 mg DSPE-PEG_2000_-NH_2_ in a round bottom flask. Chloroform was then removed by evaporating slowly under argon atmosphere for 24 h at room temperature. Then, the obtained mixed film was hydrated with MilliQ water (5 mL), and the obtained hydrophilic DCNPs modified with amino phospholipids could be dispersed greatly after vigorously sonication. Excess lipids were purified from amino groups modified DCNPs by centrifugation (105,700×*g*, 30 min) at least three times. The solution was filtered with 0.22 µm filers to remove possible large aggregates.

The synthesis of DNA functionalized DCNPs was carried out according to a published ligand-exchange method^44^. Briefly, the DSPE-PEG_2000_-NH_2_ capped DCNPs (20 μmol) in 0.8 mL of aqueous solution were carefully added to a water solution (2 mL) containing 200 nmol DNA (L_1_), and the solution was vigorously stirred overnight. Afterward, the solution of DCNPs could be clearer due to the L_1_ attachment. Then the water solution was transferred to a microtube. After vigorous sonication for several minutes, excess L_1_ was removed from DNA and amino modified DCNPs by centrifugation and washing. The large aggregates in the dispersion were removed via filtration through a 0.22 μm membrane filter. Coating L_2_ onto amino modified DCNPs was carried out all the same as that of L_1_ except 200 nmol L_2_ were used instead of 200 nmol L_1_. Finally, the two kinds of samples were dispersed in PBS (1×). Then the carboxylic acid groups on FSH_β_ were activated by EDC (N-(3-dimethylaminopropyl)-N′-ethylcarbodiimide hydrochloride) and NHS (N-hydroxysuccinimide). In a typical procedure, 1 mg of FSH_β_ was first dissolved in 5 mL 2-(N-morpholino) ethanesulfonic acid (MES) buffer (0.1 M, pH 5), 8 mg EDC was added to above solution under stirring, then 8 mg NHS was added, the resulting mixture was stirred for 30 min at room temperature.

Then 0.1 mmol of amino and L_1_ functionalized DCNPs was added into the solution and stirred vigorously for 24 h. The resulting L_1_ and FSH_β_ modified DCNPs (DCNPs-L_1_-FSH_β_) were finally centrifuged at 105,700 × g, washed several times with MES buffer to remove unreacted FSH_β_ followed by lyophilizing. DCNPs-L_2_-FSH_β_ was carried out all the same as that of L_1_ except 0.1 mmol of amino and L_2_ functionalized DCNPs were used instead of 0.1 mmol of amino and L_1_ functionalized DCNPs. Finally, the two kinds of samples ((DCNPs-L_1_-FSH_β_ and DCNPs-L_2_-FSH_β_) were dispersed in PBS (1×).

### Different ovarian tumor models preparation

Subcutaneous tumor model was prepared as following. Animal procedures were in agreement with the guidelines of the Institutional Animal Care and Use Committee of Fudan University and performed in accordance with the institutional guidelines for animal handling. CaOV_3_ cells were provided by American Type Culture Collection (ATCC, Manassas, VA, USA). CaOV_3_ cells (5 × 10^5^ dish^−1^) were seeded in cell culture flash in 8 mL of DMEM medium supplemented with 10% FBS and 1% antibiotics and incubated in CO_2_ for 24 h at 37 °C. Then CaOV_3_ tumor cells were harvested by centrifugation and resuspended in sterile PBS. CaOV_3_ cells (5 × 10^7^ cells mouse^−1^) were implanted subcutaneously into the right fore leg of 5-week-old female mice. When the tumors reached 0.2–0.7 cm in diameter (12–28 days after implant), the tumor-bearing mice were subjected to imaging studies. A2780 and H08910 epidermal ovarian tumor models were prepared as the same procedure, except for A2780, H08910 cell line was used instead of CaOV_3_ cell line, reactively. The FSHR expression of all cell lines were analyzed by immunohistochemical, western bloting (Supplementary Fig. 55), and the real-time quantitative polymerase chain reaction methods.

Peritoneal ovarian metastasis model was prepared as follows: CaVO_3_ cells (5 × 10^5^ dish^−1^) were seeded in cell culture flash in 8 mL of DMEM medium supplemented with 10% FBS and 1% antibiotics and incubated in CO_2_ for 24 h at 37 °C. Then CaOV_3_ tumor cells were harvested by centrifugation and resuspended in sterile PBS. CaOV_3_ cells (5 × 10^7^ cells mouse^−1^) were intraperitoneally injected into 5-week-old female mice. Tumor size was observed under scarified mice from the first week.

Popliteal lymph node metastasis model was prepared as following. Five-week-old mice were inoculated in the left hind paw with CaOV_3_ cells (5 × 10^8^ cells mouse^−1^). After 21 days intradermal injection, tumors cells spontaneously formed lymph node metastasis in the popliteal lymph nodes.

### In vivo assembly in subcutaneous human ovarian tumor

The in vivo experiments were carried out when the tumor size was ~1–7 mm. For the two-staged in sequence injection, in order to obtain the optimal time interval of the two injections, the first injection of DCNPs-L_1_-FSH_β_ (7.5 mg kg^−1^) was administrated by caudal vein injection, then DCNPs-L_2_-FSH_β_ (second injection, 7.5 mg kg^−1^) was injected at 4, 6, 8, and 10 h PI of first injection. Meanwhile the NIR-II fluorescence bioimaging results at different time point after first injection were obtained by 808 nm laser irradiation (fluence rate = 40 mW cm^−2^). In order to obtain the half-life of the nanoprobes in blood in each group, blood was collected at different time points of the first injection for ICP-MS analysis. The superior T/N ratio was realized when the second was administrated at 8 h PI of first injection. For the later in vivo assembly experiment, DCNPs-L_1_-FSH_β_ (7.5 mg kg^−1^) was administrated by tail injection, after 8 h PI of first injection, the second injection (DCNPs-L_2_-FSH_β_, 7.5 mg kg^−1^) was also administrated by tail injection. NIR-II fluorescence bioimaging results were obtained from 0.5 h ~28 PI of first injection. Tumors, organs, blood, and urine were collected and weighed for biodistribution analysis by ICP-MS.

### In vivo assembly in the peritoneal ovarian metastasis

The in vivo experiments were carried out when the cells were intraperitoneally injected at 7, 12, 17, and 22 days. The procedure was the same as subcutaneous ovarian tumor. As briefly, the second injection was administrated at 8 h PI of the first injection, and the peritoneal ovarian metastases were observed by NIR-II fluorescence bioimaging under 808 nm laser irradiation (laser output power density = 0.2 W cm^−2^, fluence rate 40 mW cm^−2^, and working distance = 30 cm).

### In vivo assembly in the popliteal lymph node metastasis

The in vivo experiments were carried out when the cells were inoculated in the right hind paw for 21 days. The procedure was the same as subcutaneous ovarian tumor. As briefly, the second injection was administrated at 8 h PI of the first injection, and the popliteal lymph node metastases were observed by NIR-II fluorescence bioimaging under 808 nm laser irradiation (laser output power density = 0.2 W cm^−2^, fluence rate = 40 mW cm^−2^, and working distance = 30 cm).

### Visualization of assembled DCNPs in tumor under TEM

A group of mice bearing subcutaneous human ovarian adenocarcinoma were administrated with single injection of DCNPs-L_1_-FSH_β_ (first injection) and another group of mice were injected with DCNPs-L_2_-FSH_β_ (second injection) after 8 h PI of first injection. The tumors of 12 h PI of the single first injection in the first group and 12, 15, and 18 h PI of second injection in the second group were resected and then sliced up by freezing microtome. After being washed by PBS (1×) for three times, all the tumor slice were freeze-dried for 24 h. The samples were analyzed by TEM.

### Surgical resection surgery of tumors

The epidemic tumors can be observed under NIR-II fluorescence bioimaging in the optimal surgical time window (20–26 h PI of first injection). Tumors were resected in 20, 22, 24, 25, and 26 h PI of first injected under NIR-II fluorescent bioimaging guidance. Meanwhile, tumors were also removed out of the optimal surgical time window (28 h PI of the first injection). All the tumors were further analyzed by H&E staining. The large metastatic tumors and small eye-invisible lesions in peritoneal ovarian metastasis mice were removed under InGaAs camera with 808 nm laser irradiation (laser output power density = 0.2 W cm^−2^, fluence rate = 40 W cm^−2^, and working distance = 30 cm) at 22 PI of the first injection. All collected tissues were further analyzed by H&E staining. The early invisible lesions were resected under the same procedure. Finally, the peritoneal ovarian metastases with different stages were also removed under the same procedure (Supplementary Fig. 56).

### Data availability

Data supporting the findings of this study are available within the article and the associated Supplementary information Section. Any other data are available from the corresponding authors upon reasonable request.

## Electronic supplementary material

Supplementary Information

Description of Additional Supplementary Files

Supplementary Movie 1

Supplementary Movie 2
