# Supplementary material for: NIR-II nanoprobes in-vivo assembly to improve image-guided surgery for metastatic ovarian cancer
Source: Nat Commun. 2018 Jul 24;9:2898. doi: 10.1038/s41467-018-05113-8 (PMC6057964; doi:10.1038/s41467-018-05113-8)
Supplement: Supplementary file 1 — Supplementary Information [file 41467_2018_5113_MOESM1_ESM.docx]

**NIR-II Nanoprobes *in-vivo* Assembly to Improve Image-guided Surgery for** **Metastatic** **Ovarian Cancer**

Peiyuan Wang^1^, Yong Fan^1^, Lingfei Lu^1^, Lu Liu^1^, Lingling Fan ^2,3^*, Mengyao Zhao^1^, Yang Xie^4^, Congjian Xu^2,3^, Fan Zhang ^1^*

^1^Department of Chemistry, Shanghai Key Laboratory of Molecular Catalysis and Innovative Materials, State Key Laboratory of Molecular Engineering of Polymers and i*Chem*, Fudan University, Shanghai 200433, P. R. China.

^2^Department of Obstetrics and Gynecology of Shanghai Medical School, Fudan University, Shanghai 200032, P. R. China.

^3^Obstetrics and Gynecology Hospital, Fudan University, Shanghai 200011, P. R. China.

^4^Department of Orthopedics, Changhai Hospital, Second Military Medical University, Shanghai, 200433, P. R. China.

*To whom correspondence should be addressed.

E-mail: zhang_fan@fudan.edu.cn, fanlglg@hotmail.com

**Part A: Supplementary Experimental Section**

**1. Materials and characterization.**

***Materials.*** All solvents used were of analytical grade without further purification. Gadolinium (III) chloride anhydrous (GdCl_3_, 99.99 %), neodymium (III) chloride hexahydrate (NdCl_3_, 99.9 %), sodium trifluoroacetate (Na-TFA, 98 %), 1-octadecene (ODE, 90 %), oleic acid (OA, 90 %) were purchased from Sigma-Aldrich. Follicle-stimulating hormone receptor was supplied by Shanghai Qiangyao Biotechnology Co., Ltd., the complementary DNA was provided from Shanghai Jieli Biotechnology Co., Ltd., (ethylene glycol)–distearoyl phosphatidylethanolamine, (DSPE-PEG_2000-_NH_2_) were purchased from Avanti Polar Lipids.

***Characterization.*** Transmission electron microscopy (TEM) measurements were carried out on a JEM 2100F microscope (Japan) operated at 200 kV. The samples were first dispersed in ethanol and then collected by using copper grids covered with carbon films for measurements. NIR-II fluorescence spectra were recorded on Edinburgh Fluorescence Spectrometer FLS980 instrument with excitation source were using external 808-nm semiconductor laser (Changchun New Industries Optoelectronics Tech. Co., Ltd.), unless otherwise specified, all spectra were collected under identical experimental conditions. NIR-II fluorescence imaging was carried by Nirvana InGaAs CCD camera under 808 nm irradiation (laser output power density = 0.2 W cm^-2^, fluence rate = 40 mW cm^-2^). Visible Fluorescence spectra were recorded on Edinburgh Fluorescence Spectrometer FLS980 instrument with Xenon lamp as excitation source.

**2. Lanthanide doped core-shell structured NaGdF_4_: 5%Nd@NaGdF_4_ downconversion nanoprobes synthesis.**

***Preparation of shell precursors for the synthesis of downconversion nanoparticles. Gd-OA (0.10 M) host precursor*:** A mixture of GdCl_3_ (2.50 mmol), OA (10.0 mL), and ODE (15.0 mL) was loaded in a reaction container and heated at 140 °C under vacuum with magnetic stirring for 30 min to remove residual water and oxygen. Then the colorless Gd-OA precursor solution (0.10 M) was obtained.

***Na-TFA-OA (0.40 M) precursor*:** A mixture of Na-TFA (4.00 mmol) and OA (10.0 mL) was loaded in a container at room temperature under vacuum with magnetic stirring to remove residual water and oxygen. Then the colorless Na-TFA-OA precursor solution (0.40 M) was obtained.

***Synthesis of NaGdF_4_*:*5 % Nd core nanocrystals***: The NaGdF_4_:5% Nd core nanoparticles were prepared by the thermolysis method. GdCl_3_ (0.95 mmol), NdCl_3_ (0.05 mmol), OA (6.0 mL) and ODE (15.0 mL) were mixed together and heated to 140 °C under vacuum until a clear solution formed, after that, the solution was cooled down to room temperature. A solution of NaOH (2.5 mmol) and NH_4_F (4.0 mmol) in methanol (10 mL) was added and the resultant mixture was stirred for 1 h. The reaction mixture was then heated to 70 °C and maintained for half an hour to remove the methanol. Afterward, the solution was heated to 300 °C and maintained for 90 min under a gentle argon flow. Then, the solution was cool down to room temperature and the nanoparticle products were centrifuged and washed twice with ethanol. The nanoparticles were finally dispersed in 10 mL of cyclohexane for further use.

***Synthesis of NaGdF_4_*:*5 %Nd@NaGdF_4_ core-shell*** ***structured nanocrystals***: The core-shell nanoparticles were fabricated by using the one-pot successive layer-by-layer (SILAR) protocol, which was developed by our group. 5 mL as prepared NaGdF_4_:5% Nd (~ 0.5 mmol) was mixed with 8.0 mL of OA and 12.0 mL of ODE. The flask was pumped down at 70 °C for 30 min to remove cyclohexane, any residual air. Subsequently, the system was switched to Ar flow and the reaction mixture was further heated to 280 °C at a rate of ~ 20 °C min^-1.^ Then Gd-OA (0.10 M, 1.0 mL) and Na-TFA-OA (0.40 M, 0.5 mL) shell precursors were alternately introduced by dropwise addition at 280 °C and the time interval between each injection was 15 min. There are two groups of Gd-OA (0.10 M, 1.0 mL) and Na-TFA-OA (0.40 M, 0.5 mL). Finally, the obtained NaGdF_4_: 5 %Nd@NaGdF_4_ core-shell nanoparticles were precipitated and washed and dispersed in cyclohexane.

**3. DNA and FSH_β_ modified DCNPs assembly *in vitro***

***TEM image of DCNPs assembly***: For TEM characterization, the obtained DCNPs-L_1_-FSH_β_ nanoprobes were incubated with 20 nmol DCNPs-L_2_-FSH_β_ in Tris−HCl buffer (20 mM, 100 mM NaCl, 2 mM KCl, 1 mM MgCl_2_, pH = 7.4) for 2 h. The resulted assembled DCNPs were redispersed in the buffer and stored at 4 °C for further use.

***Cell viability***: All cell lines were provided by American Type Culture Collection (ATCC, Manassas, VA, USA). The cytotoxicity was measured by using Cell Counting Kit-8 (CCK-8) assay in CaOV_3_ cells. The cells (1×10^4^) were incubated in each well of a 96-well plate for 24 h, then incubated with DCNPs-L_1_-FSH_β_ with different concentrations for 2 h, after being washed by PBS (1 X) for three times, DCNPs-L_2_-FSH_β_ was added for another 2 h incubation. Enzyme dehydrogenase in living cells was oxidized by this kit to orange carapace. The quality was assessed calorimetrically by using a multi-reader (TECAN, Infinite M200, Germany). The measurements were based on the absorbance values at 450 nm. Following formula was used to calculate the viability of cell growth:

Viability (%) = (mean absorbance value of treatment group/mean absorbance value of control group) × 100.

***Photostabilities of ICG and DCNPs***: The NIR-II fluorescence images of ICG and DCNPs were obtained under 808 nm laser irradiation (laser output power density = 0.2 W cm^-2^, fluence rate = 40 mW cm^-2^) for different time. Then the stability of DCNPs-L_1_-FSH_β_ in serum, PBS, water and blood was further evaluated by dynamic light scattering (DLS) and the size distribution of assembled nanoclusters after exposure with 808 nm laser (laser output power density = 0.2 W cm^-2^, fluence rate = 40 mW cm^-2^) for different time was also analyzed by DLS .

***Western blot***: CaOV_3_, HO8910 and A2780 cells (obtained from ATCC) were incubated with follicle-stimulating hormones receptor (FHSR) antibody (catalogue number, ab150557 and dilution ratio, 1:2000). Cell extracts were prepared using the RIPA kit (Bogoo). First, cell extracts and separate proteins were loaded on an SDS polyacrylamide gel. The electrophoresed proteins were transferred onto a 0.45 μm nitrocellulose (NC) filter membrane and incubated for 30 min at room temperature in 3 % BSA-TBST blocking solution. Next, the membrane was washed at room temperature by TBST for five times, followed by incubation overnight at 4 °C in blocking buffer containing primary antibodies. Then, the membrane was washed with TBST for five times, and incubated at room temperature for 40 min in blocking buffer containing a secondary reagent goat anti-mouse-IgG (H+L) HRP at 1:10000. Finally, after washing the membrane at room temperature for 6 times by TBST buffer, the biomarkers were detect with chemiluminescence reagents.

***Immunohistochemistry test***: CaOV_3_, HO8910 and A2780 cells were grown on glass coverslips in 24-well plate for 24 h. Then cells were fixed using 4 % paraformaldehyde in PBS (pH 7.4) for 30 min at room temperature. Remove parafomaldehyde solution and rinse 3 times with PBS. Permeabilize cells with 0.3 % Triton X-100 in PBS for 15 min. Remove Triton X-100 solution and rinse 3 times with PBS. Add 0.3 % H_2_O_2_-methanol solution and incubate for 10 min. Remove incubating solution and rinse 3 times with PBS. Block with 2 % BSA in PBS for 30 min at 37 °C to block non-specific hybridization. Add FSHR antibodies (catalogue number, ab137695 and dilution ratio, 1:200) separately to cells on cover slips, incubate 1 h at 37 °C. Remove incubating solution and rinse 3 times for 5 min with PBS. Incubate all slips with a dilution of HRP-labeled secondary antibody in PBS, 2 % BSA for 30 min at 37 °C. Remove incubating solution and rinse 4 times for 5 min with PBS. Immerse every cover slip in freshly prepared DAB solution. Counterstain cells in hematoxylin for 5 min, then rinse twice with tap water. Pick up cover slips with forceps and drain away excess buffer. Place cover slip cell layer down on a glass slide containing a drop of mounting medium. Then observe in microscopes.

***Quantitative polymerase chain reaction***: Real-time qPCR amplification was performed using MX3000P real time fluorescence quantitative PCR (Stratagene, U.S.) with the Bio-Rad CFX Manager 3.1 software (Bio-Rad Laboratories, Inc., Hercules, CA, USA). Each reaction (20 mL) was contained with 10 μL of 2X qPCR Bio SyGreen Mix Low-ROX (PCR Biosystems Ltd, London, UK), 0.8 mL of each forward and reverse primer, 5 mL of a DNA template (5 ng mL^-1^), and the volume made up to 20 mL with RNase-free water. RNase-free water was added instead of cDNA template in the non-template control. Thermocycler was set for 40 cycles of denaturing at 95 °C for 10 seconds following with the optimum annealing temperature of 61.4°C, 60 °C for 40 seconds and melting temperature of 82 °C, 80°C for 10 seconds for GAPDH, FSHR respectively with a gradient from 50 °C to 95 °C to investigate the gene expression.

**4. DNA and FSH_β_ modified DCNPs *in vivo* assembly**

***Förster resonance energy transfer (FRET) of complementary DNA modified DCNPs***: Cy5 labeled L_1_, and Cy7 modified L_2_ were anchored on DCNPs, respectively. Another noncomplementary group was prepared with Cy5 and Cy7 modified L_1_ were anchored on the nanoprobes, respectively. After FSH_β_ modification, the fluorescence bioimaging of the subcutaneous ovarian tumor was carried out. Two groups of (DCNPs-L_1_(Cy5)-FSH_β_ and DCNPs-L_2_(Cy7)-FSH_β_) and (DCNPs-L_1_(Cy5)-FSH_β_ + DCNPs-L_1_(Cy7)-FSH_β_) were studied. Time interval of the two injections was 6 h. The fluorescence bioimaging was operated under 548 nm laser excitation by collecting 664 nm fluorescence at 20 h post injection (PI) of 1^st^ injection.

***In vivo assembly of nanoprobes in subcutaneous human ovarian adenocarcinoma*:** The *in vivo* experiments were carried out when the tumor size was ~ 1 mm, 2 mm. 3 mm, 4 mm, 5 mm and 7 mm. The single injection of DCNPs-L_1_-FSH_β_ (1^st^ injection, 15 mg kg^-1^) was administrated by tail injection and NIR-II fluorescence bioimaging results were obtained from 0.5 h to 28 h PI. Then the organs and tumors were collected and weighted for biodistribution analysis by inductively coupled plasma mass spectrometer (ICP-MS). Meanwhile, the blood and urine was collected at different time point after 1^st^ injection and the biodistribution of nanoprobes in blood and urine were also analyzed by ICP-MS. For *ex vivo* NIR-II bioimaging, mice were sacrificed at different time point after 1^st^ inject. Organs (heart, lung, liver, spleen and kidney) and tumors were collected and NIR-II bioimaging results were obtained by InGaAs CCD camera under 808 nm laser irradiation (fluence rate = 40 mW cm^-2^, working distance = 30 cm).

For the first non-assembling group, 1^st^ injection was administrated by tail injection, another 1^st^ was injected at 8 h PI of 1^st^ injection and NIR-II fluorescence bioimaging results were obtained at 2 h, 8 h, 14 h, 22 h and 28 h PI. Another non-assembling group was carried out as following. A single injection of amine modified DCNPs was given by caudal vein injection and NIR-II fluorescence bioimaging results were obtained at 2 h, 8 h, 14 h, 22 h and 28 h PI.

For pre-assembly groups, (1^st^ + 2^nd^) and (DCNPs-L_1_ + DCNPs-L_2_) were pre-assembled, respectively. And then the two groups of assembled DCNPs were injected to subcutaneous human ovarian adenocarcinoma bearing mice. NIR-II fluorescence bioimaging results were obtained at 2 h, 8 h, 14 h, 22 h and 28 h PI.

For the passive targeting nanoprobes group, DCNPs-L_2_ was administrated at 8 h PI of DCNPs-L_1_. NIR-II fluorescence bioimaging results were obtained at 2 h, 8 h, 14 h, 22 h and 28 h PI.

For arginine-glycine-aspartic acid (RGD) targeting motifs, DCNPs-L_2_-RGD was injected by tail after 8 h PI of the DCNPs-L_1_-RGD and NIR-II fluorescence bioimaging results were obtained at 2 h, 8 h, 14 h, 22 h and 28 h PI. .

**5. Magnetic resonance imaging in optimal surgical time window**

*In vivo* MRI was carried out on a 3.0-T clinical MRI instrument (Siemens Magnetom Trio Tim 3.0T MRI). T_1_-weighted MR images of the whole body were acquired with the turbo spin echo (TSE) sequence: TR = 529 ms; TE = 14 ms; TD = 0.0 ms; Slice thickness = 1.5 mm; Field of view (Fov) read = 60 mm; Fov phase = 75 %; matrix = 384 × 384; Flip angle =120 °C; Fat suppress, None; Water Suppress, None. T_2_-weighted MR images of the brain sections were acquired with the turbo spin echo (TSE) sequence: TR = 2500 ms; TE = 76 ms; slice tn ickness = 1.5 mm; Field of view (Fov) read = 60 mm; Fov phase = 59.9%; matrix = 384 × 384; Flip angle = 117 °C; Fat suppress, None; Water Suppress, None.” MRI was taken after NIR-II fluorescence bioimaging from 20 h to 28 h PI of 1^st^ injection. The surgical resected tumors in the optimal surgical time window were evaluated by hematoxylin and eosin (H & E) staining. The peripheral tissue after the tumor surgical resected out of the optimal surgical time window was also analyzed by H & E staining.

**Part B: Supplementary Figures**


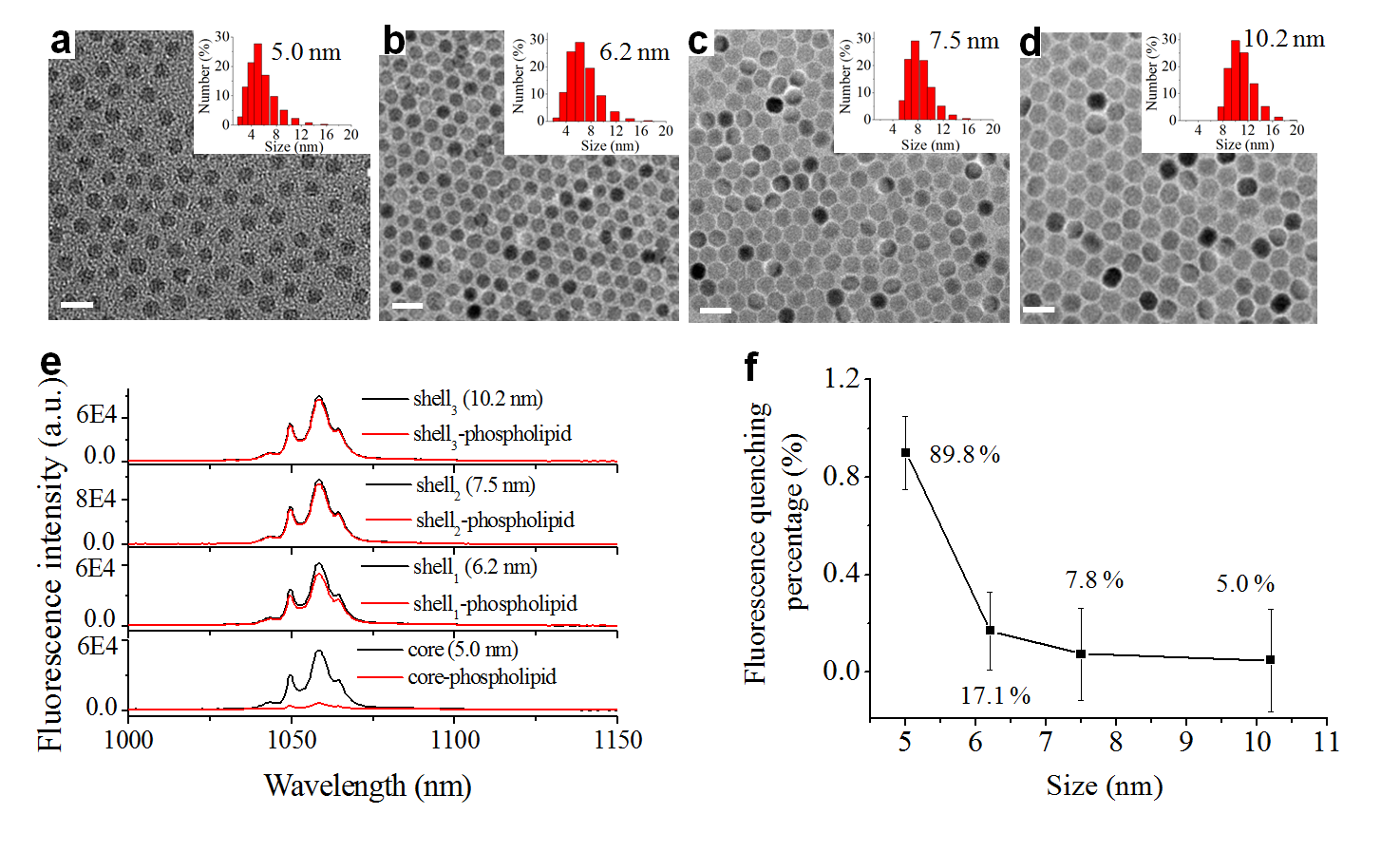


**Supplementary Figure 1.** TEM images of NaGdF_4_: 5 %Nd core nanocrystals (**a**) and NaGdF_4_: 5 %Nd@NaGdF_4_ core-shell structured nanocrystals with different shell thickness: 1.2 nm (**b**), 2.5 nm (**c**) and 5.5 nm (**d**). Insert, dynamic laser scattering (DLS) analysis of size distribution. (**e**) Fluorescence intensity of the NaGdF_4_: 5 %Nd core and NaGdF_4_: 5 %Nd@NaGdF_4_ core-shell structured nanocrystals with varied shell thickness in the aqueous solution. (**f**) Fluorescence quenching percentage of NaGdF_4_: 5 %Nd core and NaGdF_4_: 5 %Nd@NaGdF_4_ core-shell structured nanocrystals with varied shell thickness in the aqueous solution. Scale bars, 15 nm. Mean ± s.d. for n = 5.


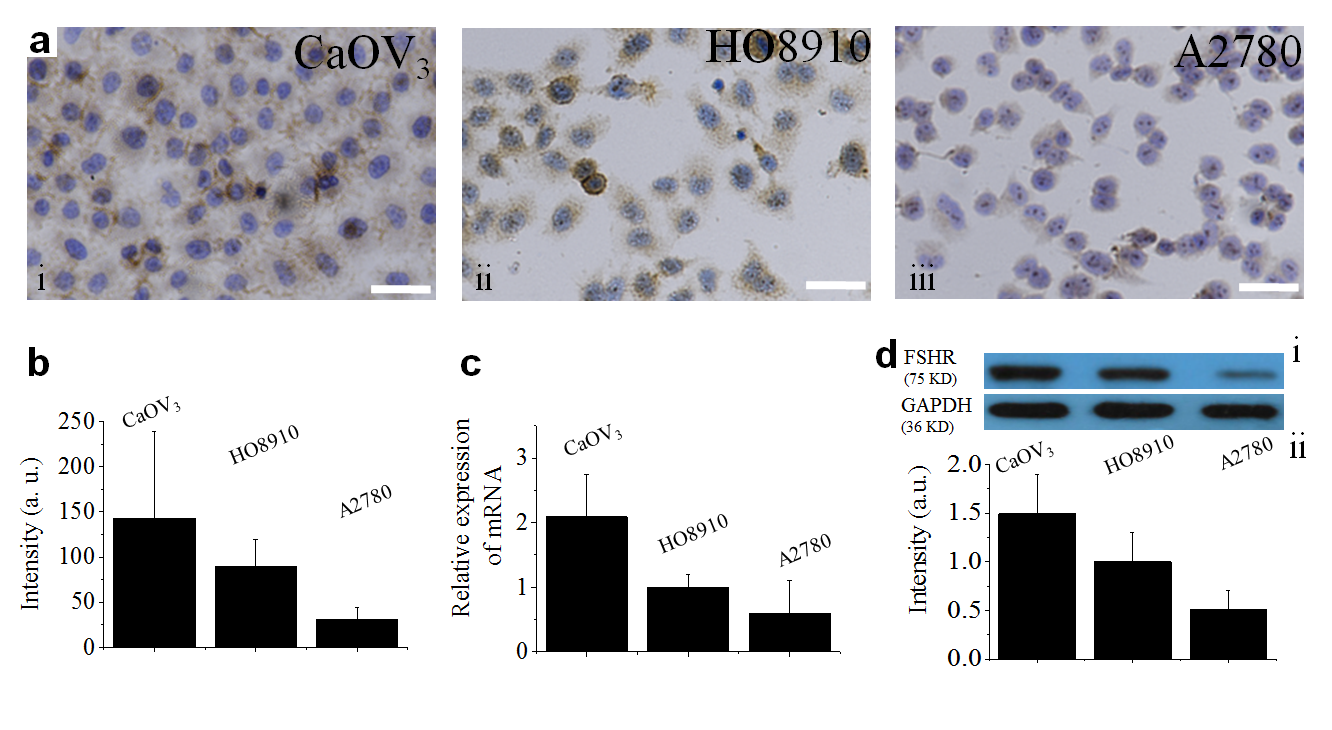


**Supplementary Figure 2.** (**a**) Expression of FSHR in the ovarian cancer cell lines CaOV_3_ (i), HO8910 (ii) and A2780 (iii) as estimated by immunohistochemical (IHC) method. (**b**) The statistical analysis of the IHC results. (**c**) mRNA relative expression of FSHR in three different cell lines. (**d**) FSHR expression in three different cell lines measured by WB method. (i) Expression of FSHR protein in three different cell lines and GAPDH (glyceraldehyde-3-phosphate dehydrogenase) is used as loading control. Statistical analysis of WB results (ii). Scale bars, 40 μm. Representative images are for n = 5 per group. Mean ± s.d. for n = 5.

**
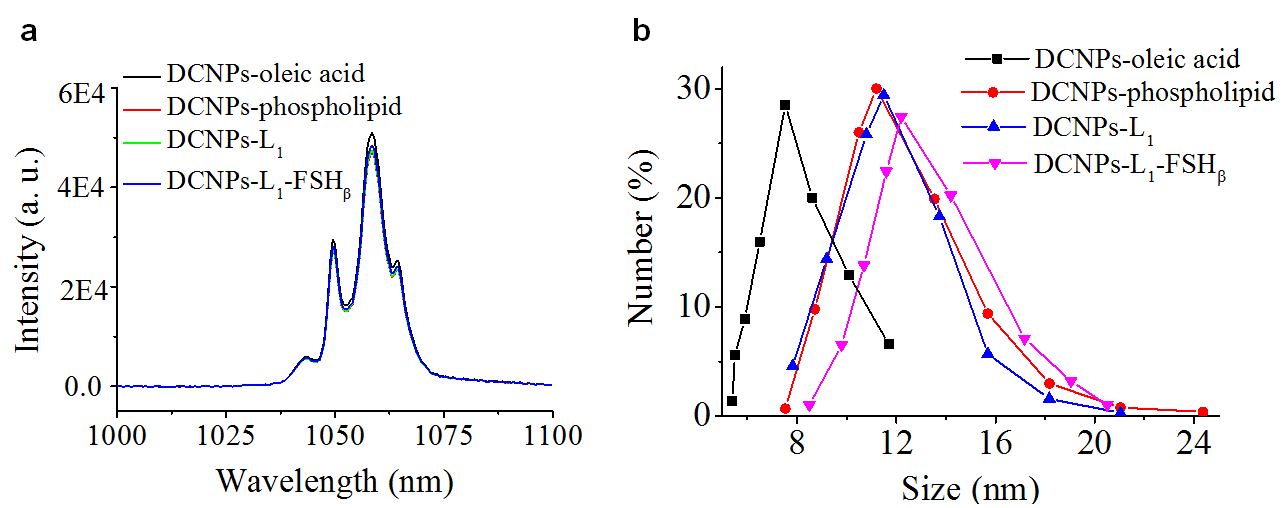
**

**Supplementary Figure 3.** (**a**) Fluorescence spectra of DCNPs-oleic acid, DCNPs-phospholipid, DCNPs-L_1_ and DCNPs-L_1_-FSH_β_. (**b)** Corresponding size distribution results measured by DLS.

**
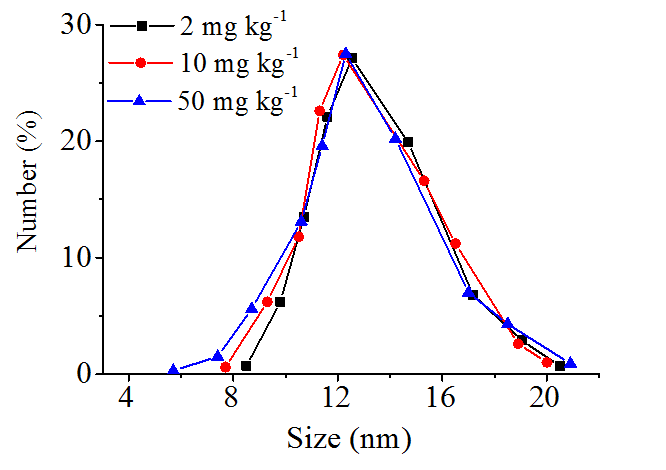
**

**Supplementary Figure 4.** DLS results for the size distribution of DCNPs-L_1_-FSH_β_ with the frequently used nanoprobes doses in aqueous solution.


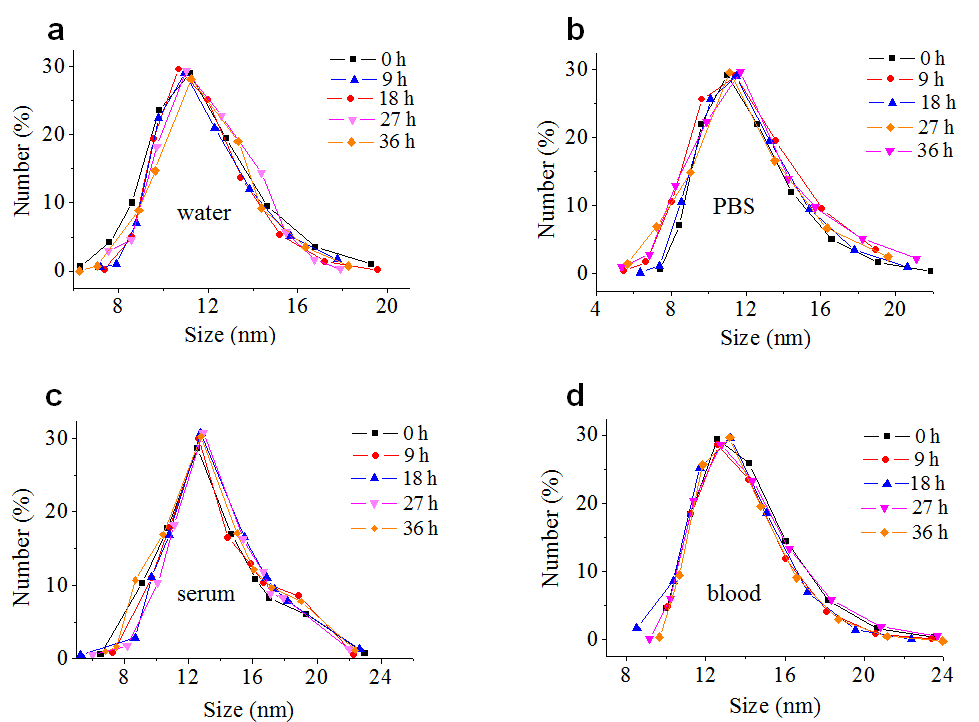


**Supplementary Figure 5.** Size distribution of DCNPs-L_1_-FSH_β_ in water (**a**), PBS (**b**), serum (**c**) and blood (**d**) at 37 °C for various time.


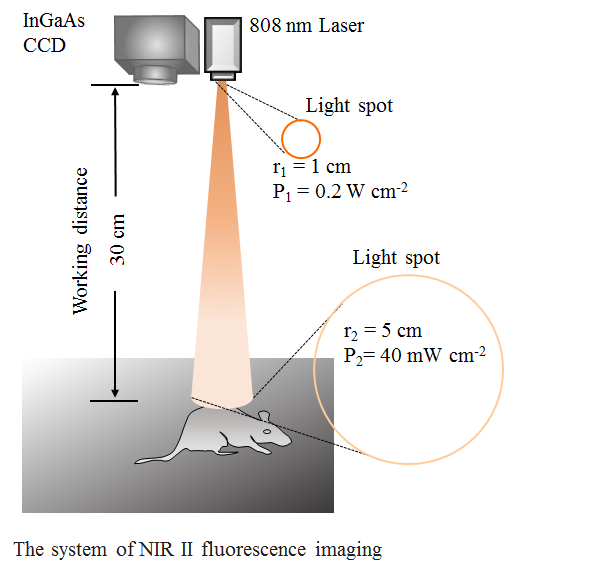


**Supplementary Figure 6.** Schematic illustration of the NIR-II fluorescence imaging system for tumor surgical guidance. ~ 30 cm working distance is used for the intraoperative imaging, which is agreement with FDA approved NIR bioimaging-guided tumor surgery with ICG. The output power density of 808 nm laser is 0.2 W cm^-2^ (P_1_) with light spot 3.14 cm^2^, and the fluence rate of the image-guided surgery platform is calculated to be 40 mW cm^-2^ with light spot 78.5 cm^2^.


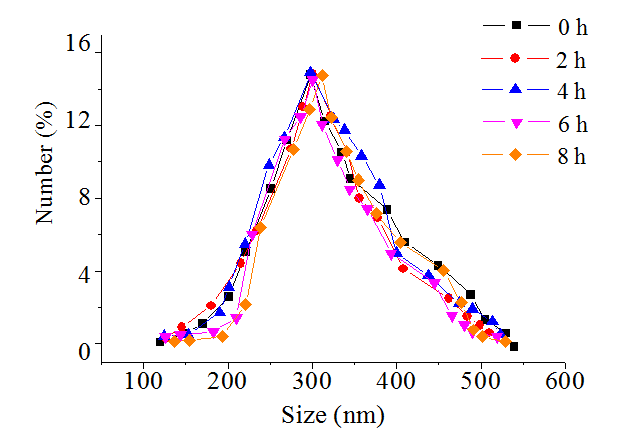


**Supplementary Figure 7.** Hydrodynamic diameters of nanoclusters in serum at 37 °C after 808 nm laser irradiation (laser output power density = 0.2 W cm^-2^, fluence rate = 40 mW cm^-2^) for various time for various hours.


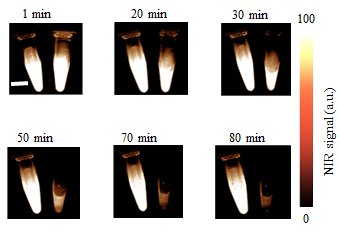


**Supplementary Figure 8.** Photostability comparison between DCNPs (left) and ICG (right) under continuous 808 nm laser irradiation for various minutes. Scale bar, 1cm. Representative images are for n = 5.


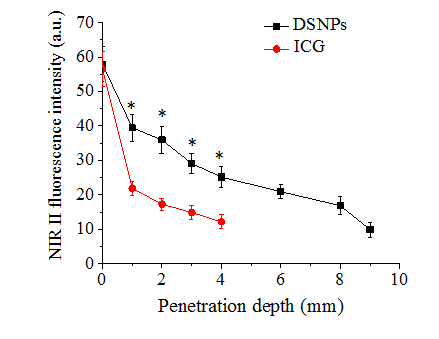


**Supplementary Figure 9.** The fluorescence intensity of ICG and DCNPs in Figure 2h. The fluorescence intensity of DCNPs was two-fold higher than ICG in 3 mm depth. Mean ± s.d. for n = 5 (*P < 0.05 versus ICG, two-sided Student^’^s t-test).


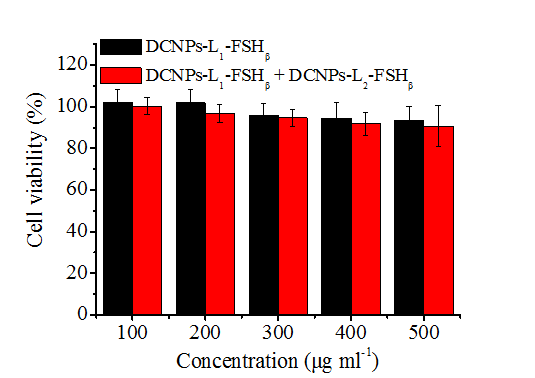


**Supplementary Figure 10.** Cell viabilities of DCNPs-L_1_-FSH_β_ and intracellular assembled DCNPs. Mean ± s.d. for n = 5.


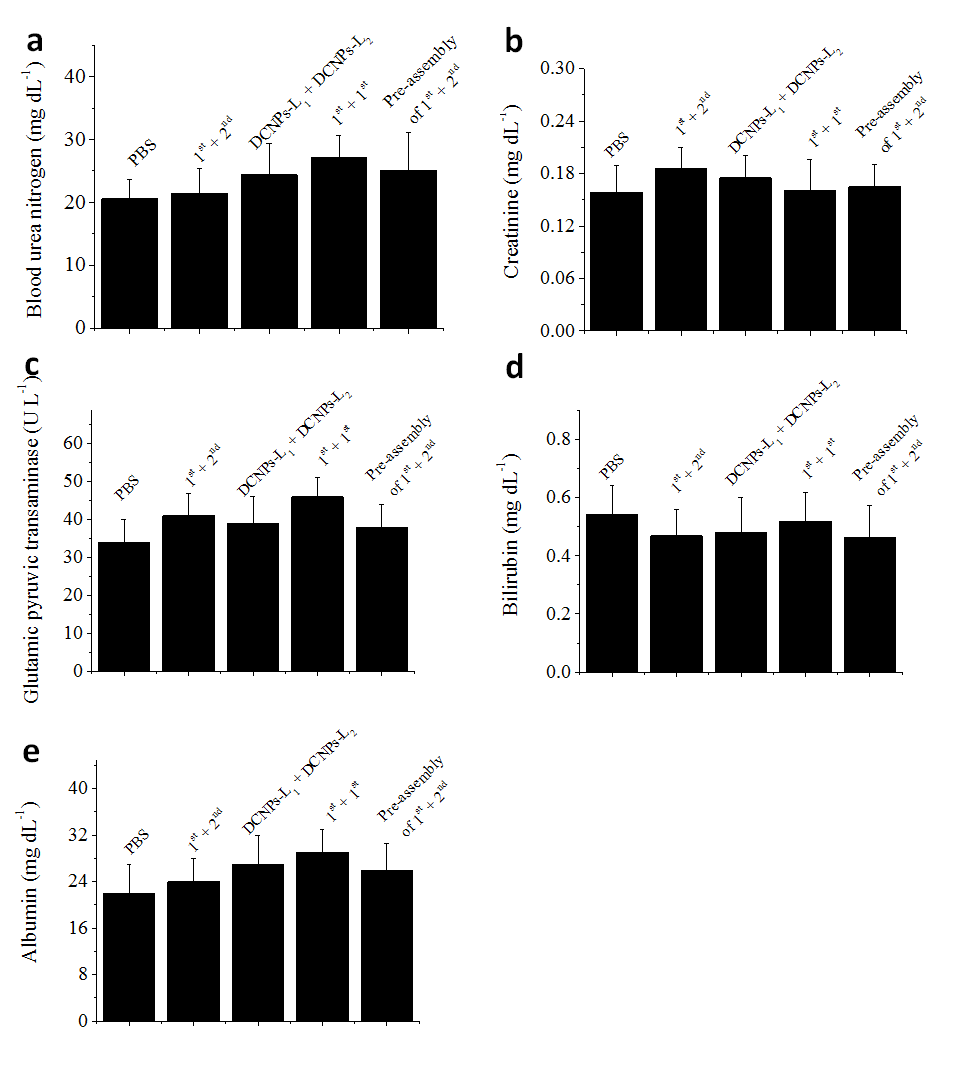


**Supplementary Figure 11.** Blood biochemistry data of blood urea nitrogen (**a**), creatinine (**b**), glutamine pyruvie transaminase (**c**), billrub (**d**) and albumin (**e**) obtained from mice after the various treatments or receiving no injection as control. Mean ± s.d. for n = 5.

**
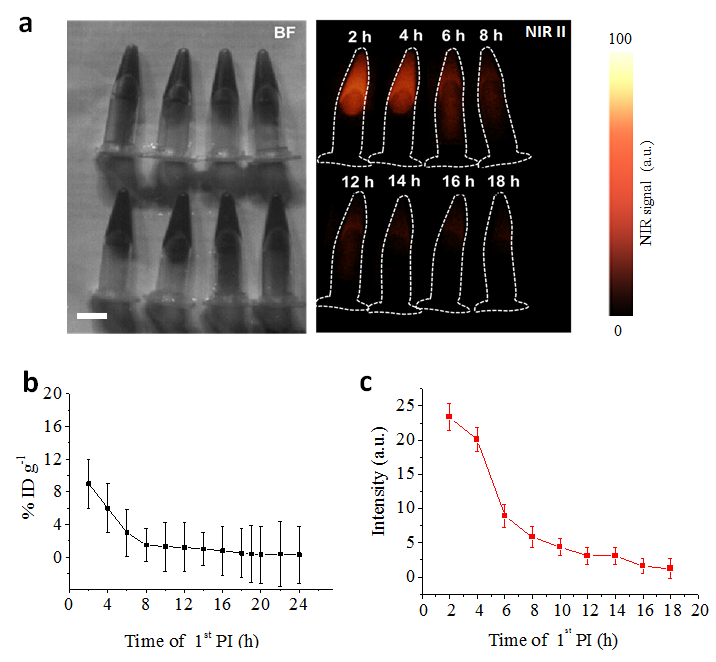
**

**Supplementary Figure 12.** (**a**) NIR-II fluorescence bioimaging results of blood samples after single injection of DCNPs-L_1_-FSH_β_. Scale bar, 1 cm. (**b**) The corresponding nanoprobes distribution in blood. (**c**) The corresponding NIR-II fluorescence intensity signals in a. Representative images are for n = 5 per group. Mean ± s.d. for n = 5.


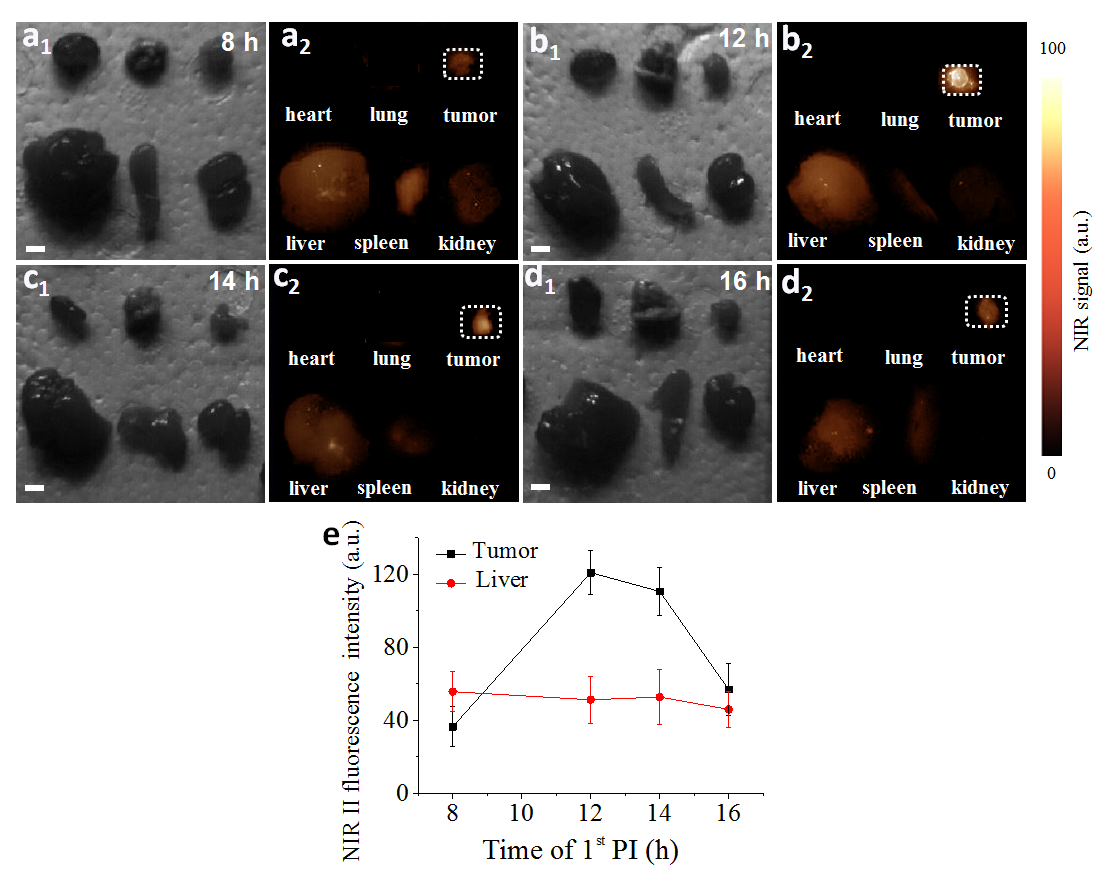


**Supplementary Figure 13.** *Ex-vivo* bright field (a_1_-d_1_) and NIR-II fluorescence images (a_2_-d_2_) of different organs and tumors which were collected after mice were sacrificed at 8 h (**a**), 12 h (**b**), 14 h (**c**) and 16 h (**d**) PI of single 1^st^ injection. Scale bars, 5 mm. (**e**) The corresponding NIR-II fluorescence intensity of liver and tumor from 8 h to 16 h PI of single 1^st^ injection. Representative images are for n = 5 per group. Mean ± s.d. for n = 5.

**
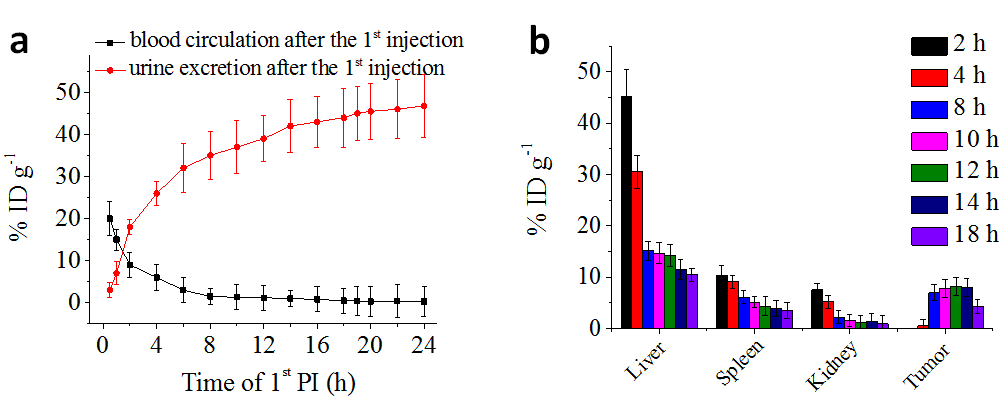
**

**Supplementary Figure 14.** (**a**) Blood circulation and urine excretion of nanoprobes after single 1^st^ injection. (**b**) RES system and tumor distribution of DCNPs-L_1_-FSH_β_. Mean ± s.d. for n = 5.

**
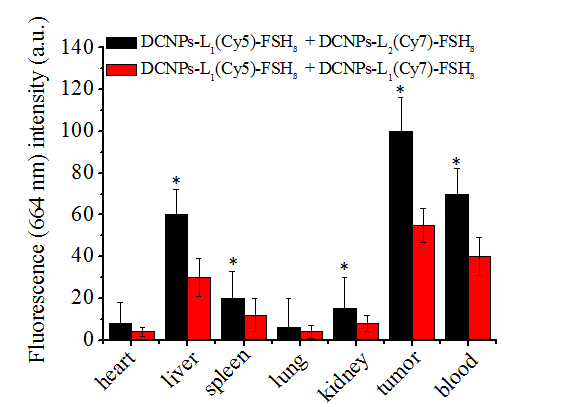
**

**Supplementary Figure 15.** Fluorescence (664 nm) intensity of different organs and tumors after two-staged in sequence injection of DCNPs-L_1_(Cy5)-FSH_β_ and DCNPs-L_2_(Cy7)-FSH_β_, DCNPs-L_1_(Cy5)-FSH_β_ and DCNPs-L_1_(Cy7)-FSH_β_, respectively. Mean ± s.d. for n = 5 (*P < 0.05 versus DCNPs-L_1_(Cy5)-FSH_β_ + DCNPs-L_1_(Cy7)-FSH_β_, two-sided Student^’^s t-test).


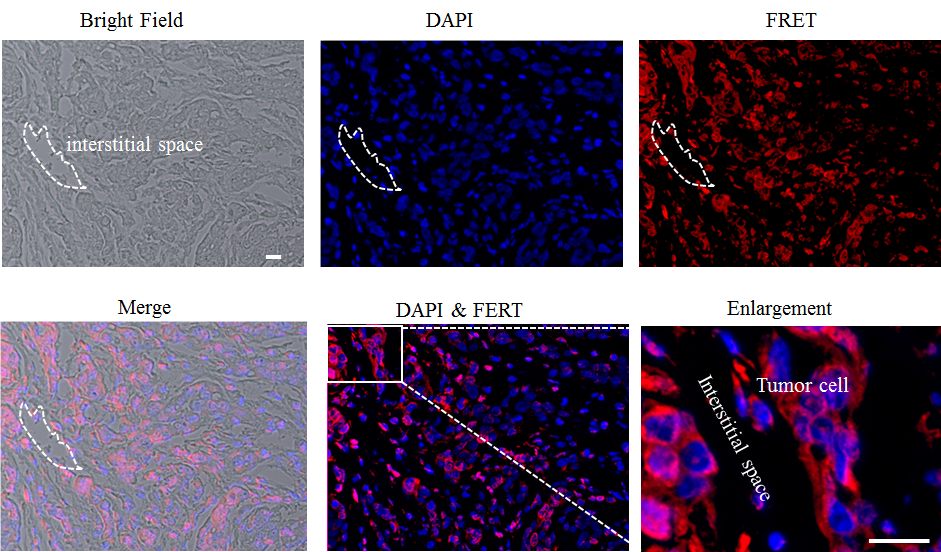


**Supplementary Figure 16.** Fluorescence images of the tumor frozen section (40 X). The dotted light refers to the interstitial space. The enlargement of the fluorescence image exhibited that the FRET signals came from the tumor cells. Scale bars, 25 μm. Representative images are for n = 5.

**
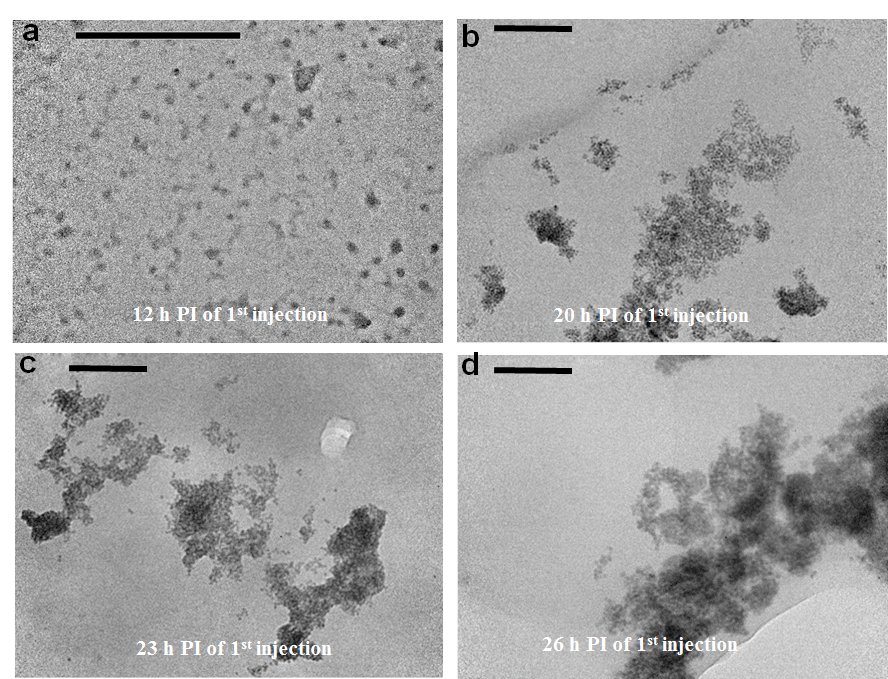
**

**Supplementary Figure 17.** TEM images of nanoprobes after 12 PI of single 1^st^ injection (**a**) (Scale bar, 50 nm) and assembled nanoprobes (**b-d**) in tumor frozen section in 20 h, 23 h and 26 h PI of 1^st^ injection (Scale bars, 200 nm). Representative images are for n = 5 per group.


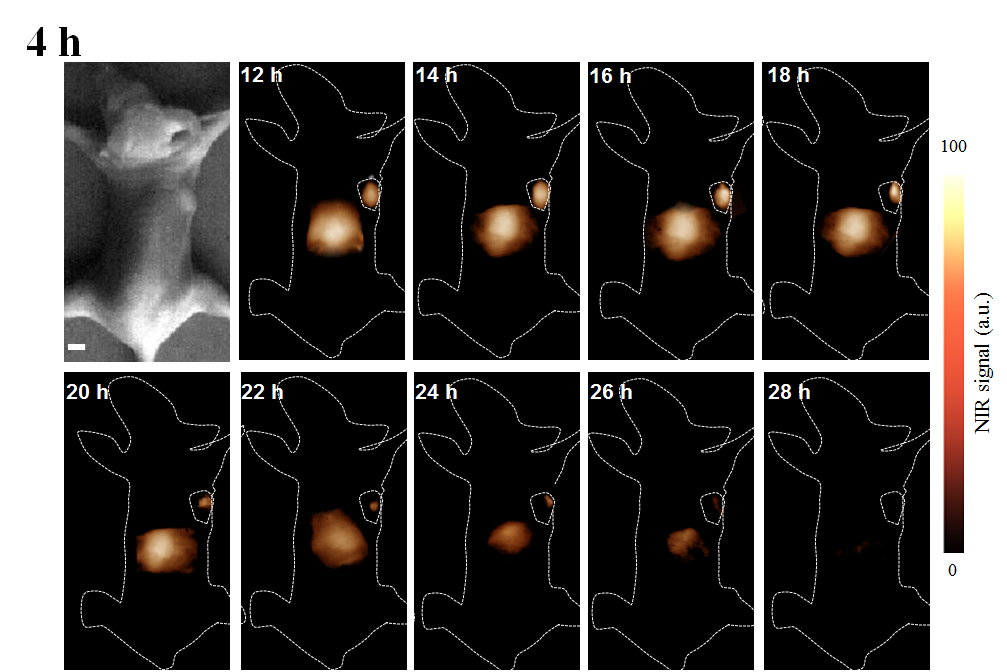


**Supplementary Figure 18.** NIR-II fluorescence bioimaging of human ovarian adenocarcinoma-bearing mice after the 2^nd^ was tail injected at 4 h PI of 1^st^ injection. Scale bar, 5 mm. Representative images are for n = 5.

**
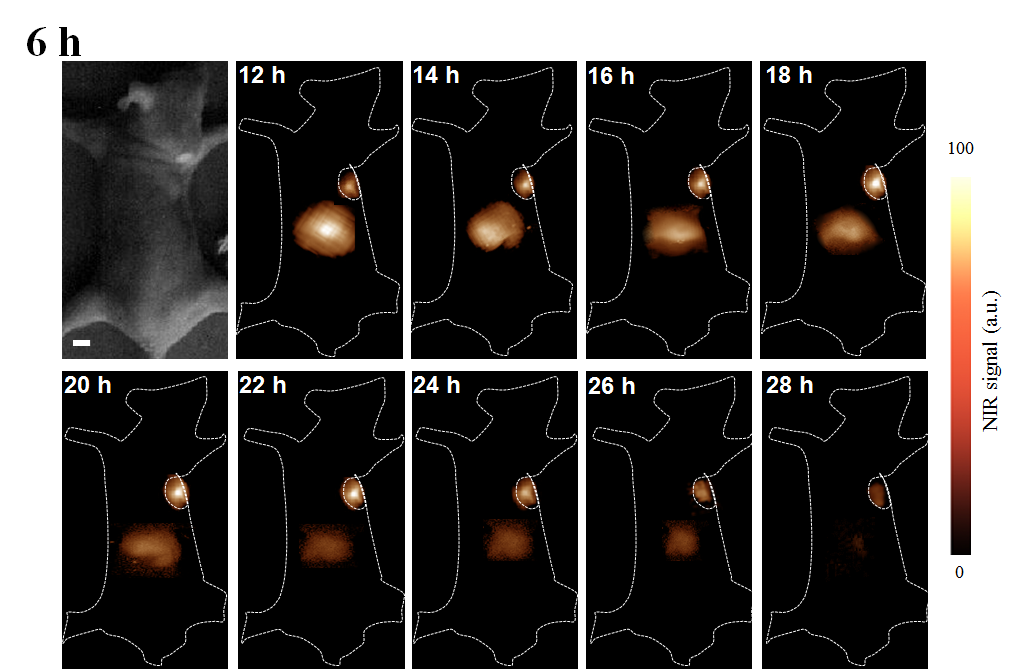
**

**Supplementary Figure 19.** NIR-II fluorescence bioimaging of human ovarian adenocarcinoma-bearing mice after the 2^nd^ was tail injected at 6 h PI of 1^st^ injection. Scale bar, 5 mm. Representative images are for n = 5.


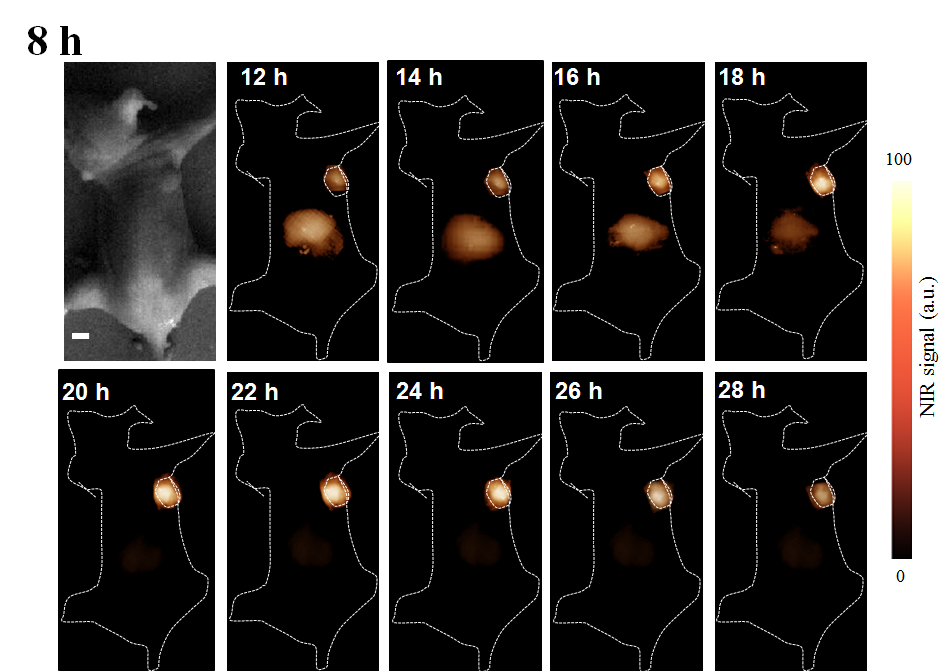


**Supplementary Figure 20.** NIR-II fluorescence bioimaging of human ovarian adenocarcinoma-bearing mice after the 2^nd^ was tail injected at 8 h PI of 1^st^ injection. Scale bar, 5 mm. Representative images are for n = 5.


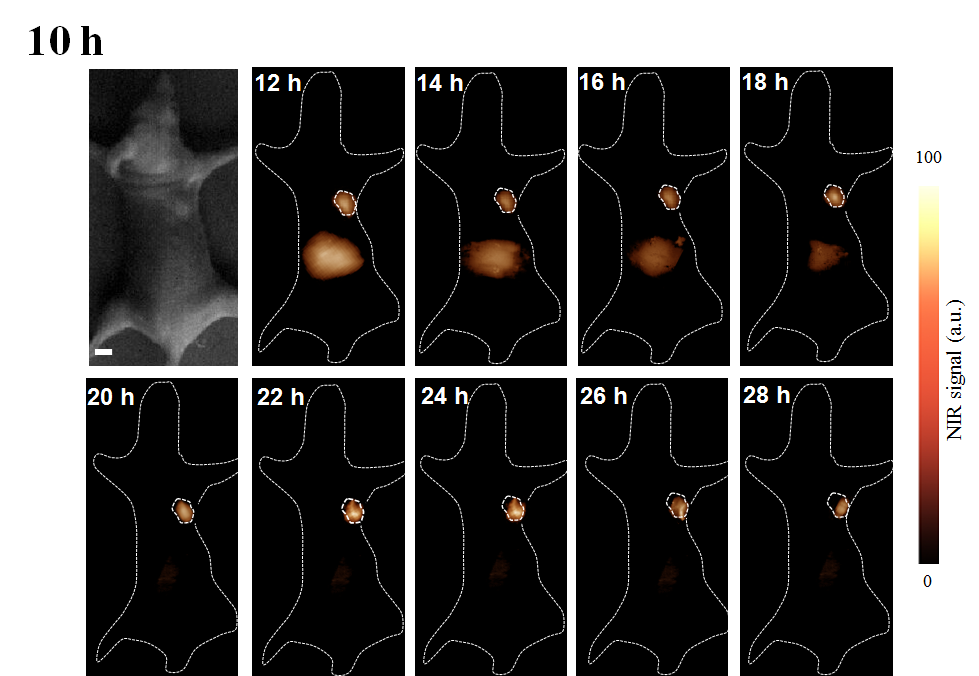


**Supplementary Figure 21.** NIR-II fluorescence bioimaging of human ovarian adenocarcinoma-bearing mice after the 2^nd^ was tail injected at 6 h PI of 1^st^ injection. Scale bar, 5 mm. Representative images are for n = 5.


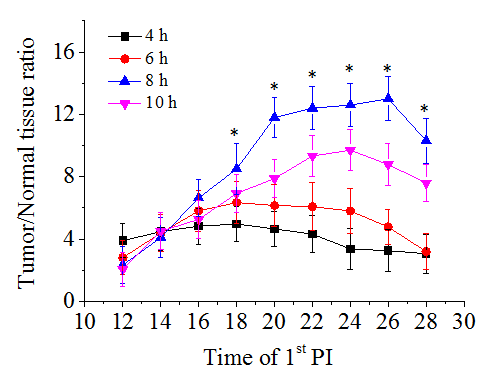


**Supplementary Figure 22.** The corresponding T/N ratios of NIR-II fluorescence imaging results obtained from Supplementary Figures 18-21 (the 2^nd^ injection was administrated at 4, 6, 8, 10 h PI of 1^st^ injection). The T/N ratio is tumor-to-liver ratio in this epidermal tumor model experiments. Mean ± s.d. for n = 5 (*P < 0.05 versus 10 h, two-sided Student^’^s t-test).


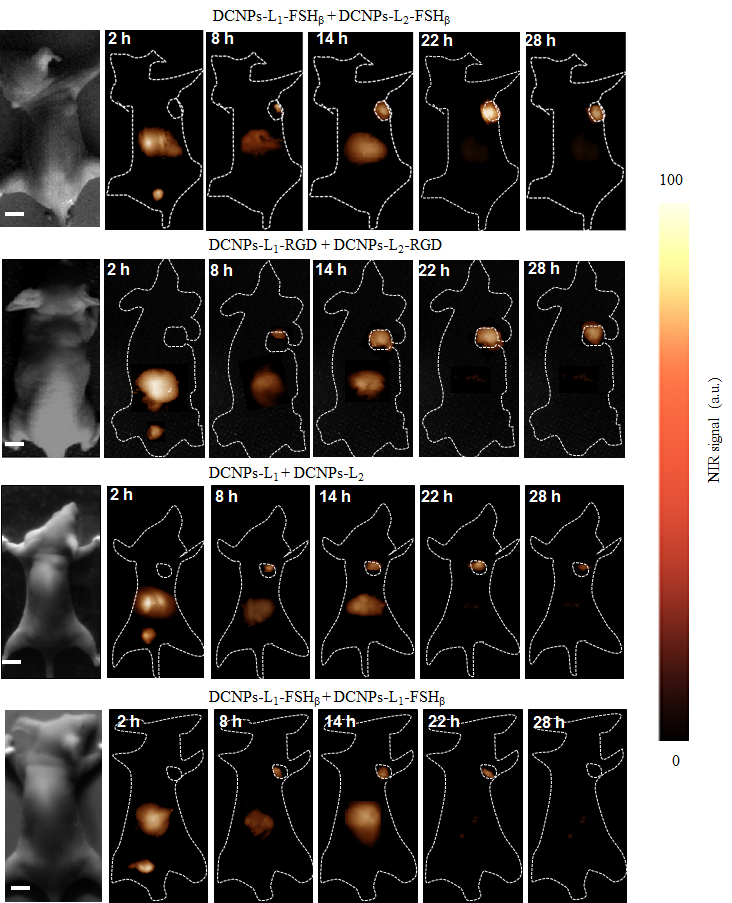


**Supplementary Figure 23.** NIR-II fluorescence bioimaging of active targeting (1^st^ + 2^nd^), RGD modified motifs (DCNPs-L_1_-RGD + DCNPs-L_2_-RGD), passive targeting (DCNPs-L_1_ + DCNPs-L_1_) and non-assembly group (1^st^ + 1^st^). Scale bars, 1 cm. Representative images are for n = 5 per group.

**
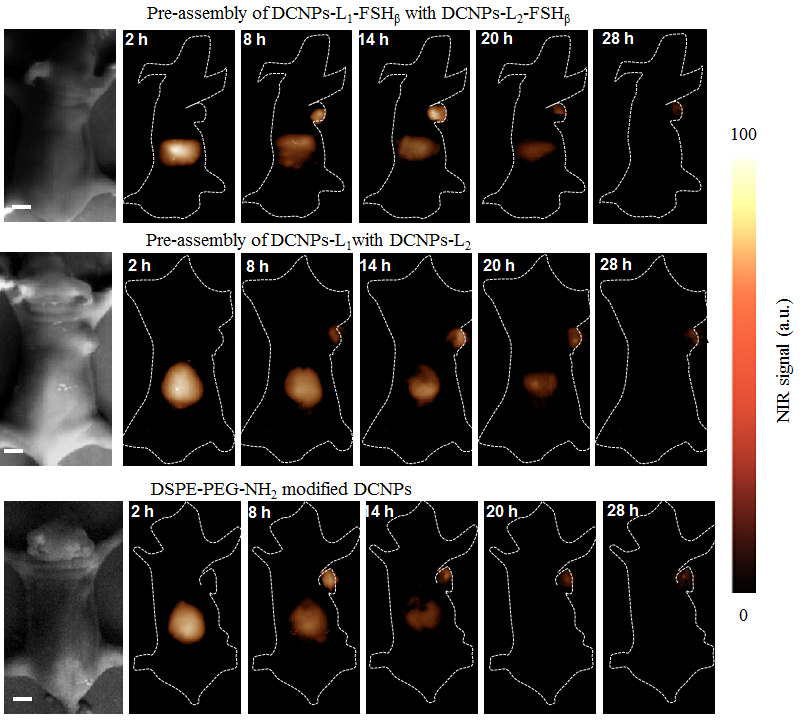
**

**Supplementary Figure 24.** NIR-II fluorescence bioimaging of pre-assembly (1^st^ + 2^nd^), passive targeting (DCNPs-L_1_ + and DCNPs-L_2_) and non-assembly group (DCNPs-DSPE-PEG_2000_-NH_2_). Scale bars, 1 m. Representative images are for n = 5 per group.

**
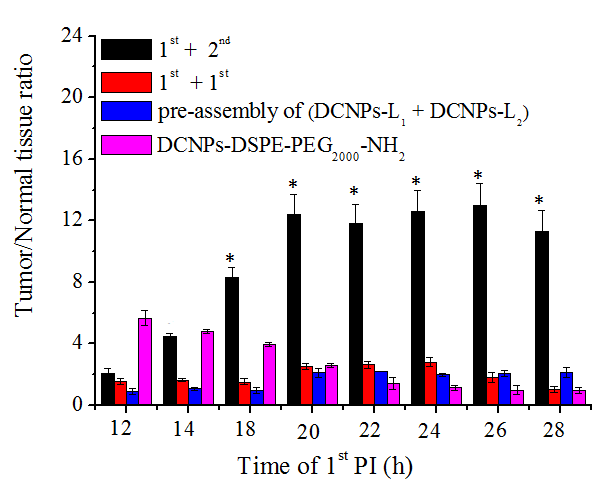
**

**Supplementary Figure 25.** T/N ratios of *in vivo* assembly (1^st^ + 2^nd^), non-assembly (1^st^ + 1^st^), pre-assembly (DCNPs-L_1_ + DCNPs-L_2_) and single injection (DCNPs-DSPE-PEG_2000_-NH_2_) groups. The T/N ratio is tumor-to-liver ratio in this epidermal tumor model experiments. The T/N ratio is tumor-to-liver ratio in this epidermal tumor model experiments. Mean ± s.d. for n = 5 (*P < 0.05, versus 1^st^ + 1^st^, two-sided Student^’^s t-test).

**
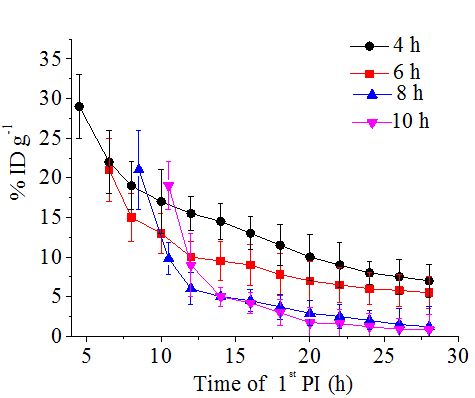
**

**Supplementary Figure 26.** The blood circulation results of human ovarian adenocarcinoma-bearing mice after the 2^nd^ injection was administrated at 4 h, 6 h, 8 h and 10 h PI of 1^st^ injection. The half-life in blood was 10 h, 6 h, 2 h and 2 h for 4 h, 6 h, 8 h and 10 h PI of 1^st^ injection, respectively. Mean ± s.d. for n = 5.

**
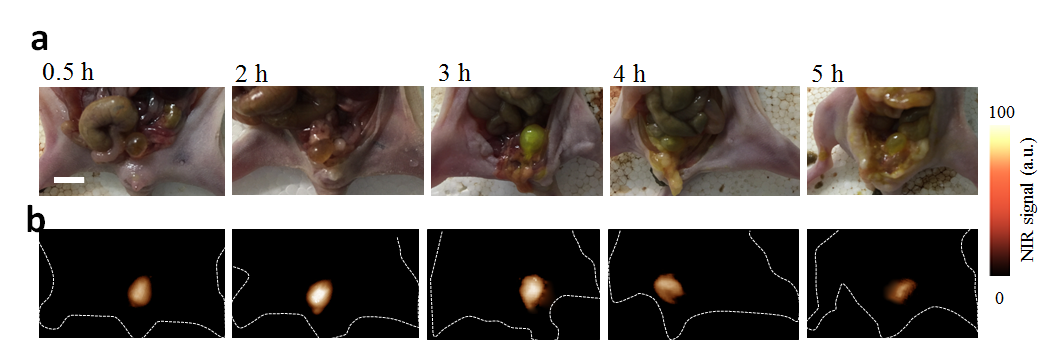
**

**Supplementary Figure 27.** Optical (**a**) and NIR-II fluorescence (**b**) images of bladder obtained at different time points after the 2^nd^ was injected. Scale bar, 1 cm. Representative images are for n = 5.

**
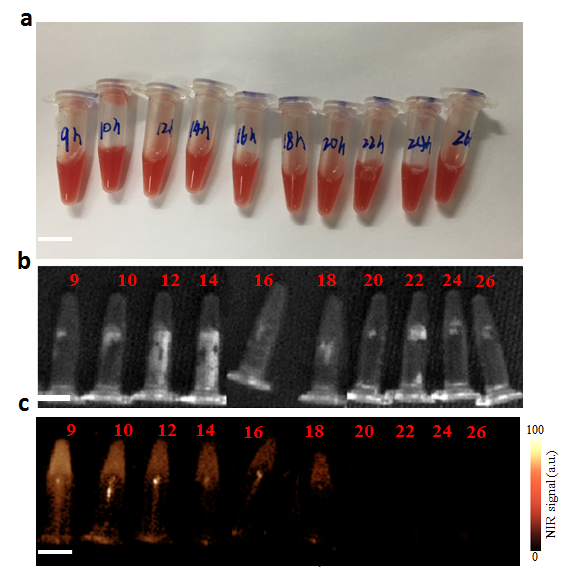
**

**Supplementary Figure 28.** (**a**) Optical photo of blood samples collected after the 2^nd^ was injected at 9 ~ 28 h PI of 1^st^ injection. The corresponding bright field (**b**) and NIR-II fluorescence images (**c**). Scale bars, 1 cm. Representative images are for n = 5.


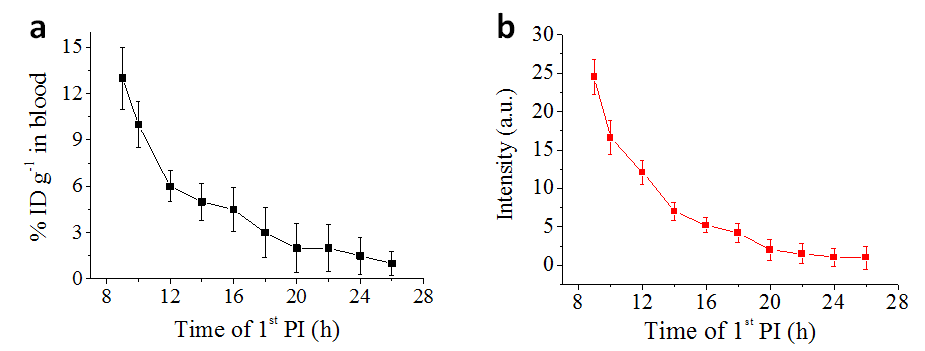


**Supplementary Figure 29.** (**a**) The nanoprobes distribution in blood collected after the 2^nd^ was injected at 9 ~ 26 h PI of 1^st^ injection. (**b**) The corresponding NIR-II fluorescence intensity in Supplementary Figure 28. Mean ± s.d. for n = 5.


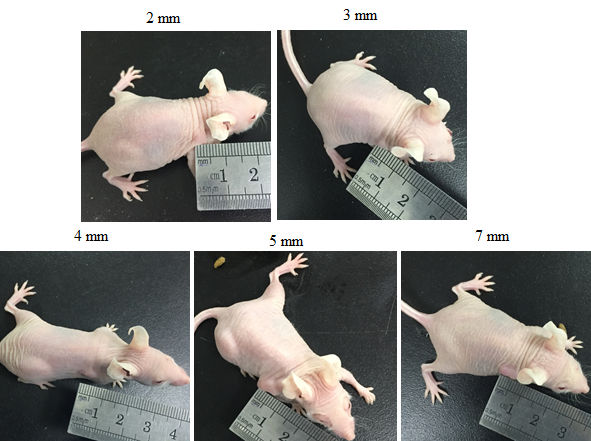


**Supplementary Figure 30.** Optical images of subcutaneous tumors-bearing nude mice with different tumor size from 2 - 7 mm.


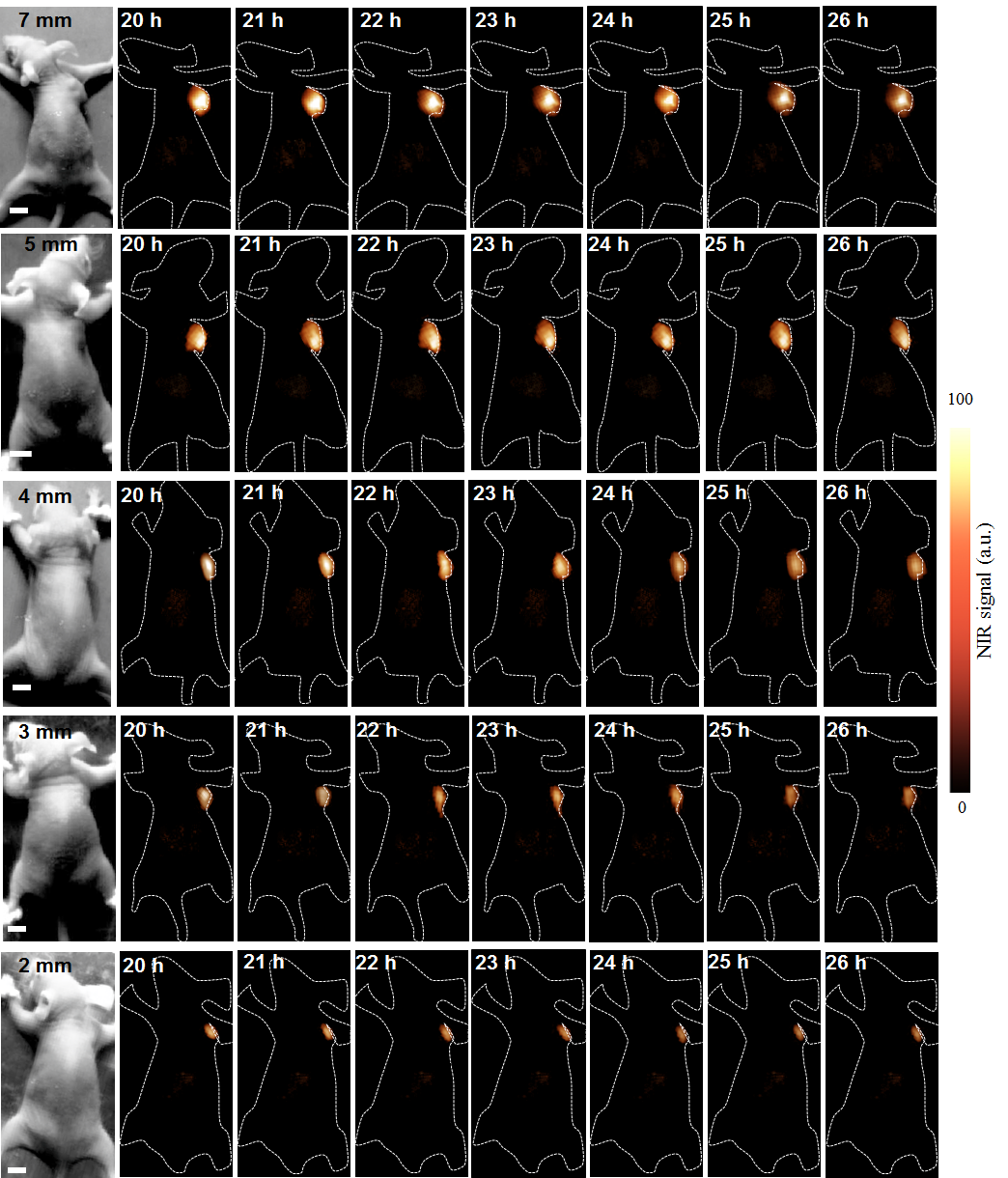


**Supplementary Figure 31.** NIR-II fluorescence bioimaging results of subcutaneous tumor-bearing mice (2 - 7 mm), which were obtained from 20 h ~ 26 h PI of 1^st^ injection. (2^nd^ was injected at 8 h PI of 1^st^ injection). All scale bars, 1 cm. Representative images are for n = 5 per group.


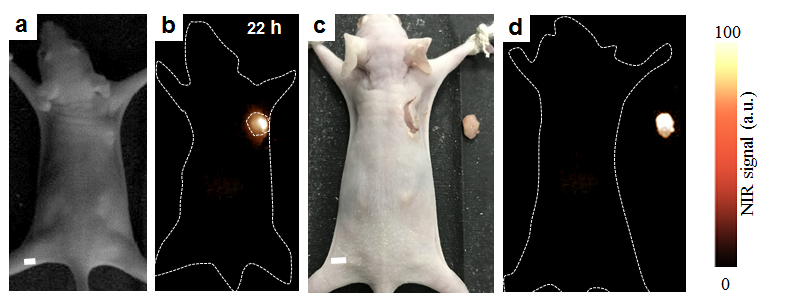


**Supplementary Figure 32.** NIR-II fluorescence bioimaging result (22 h PI of 1^st^ injection) of epidermal tumor (**a**, **b**), digital image (**c**) and NIR-II fluorescence bioimaging result (**d**) after the surgical resection. All scale bars, 5 mm. Representative images are for n = 5.


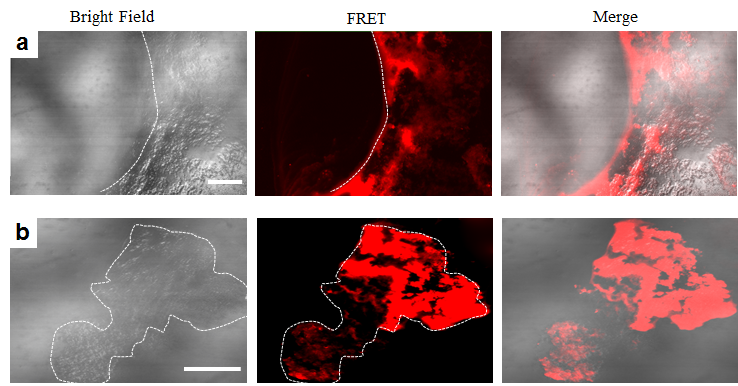


**Supplementary Figure 33.** Fluorescence images of the tumor frozen section (20 X). The subcutaneous tumors (**a**) and peritoneal metastasis lesion (**b**) were resected at 22 h PI of 1^st^ injection. The FRET fluorescence in the tumor tissues can be used to verify the distribution of DCNPs. Scale bars, 25 μm. Representative images are for n = 5.


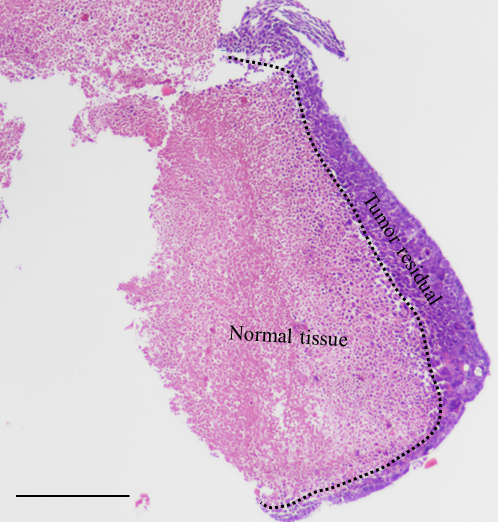


**Supplementary Figure 34.** H & E staining result of tissue residual on the normal tissue after tumor surgical resection was operated out of the optimal surgical time window (28 h PI of 1^st^ injection). Scale bar, 0.2 mm. Representative images are for n = 5.


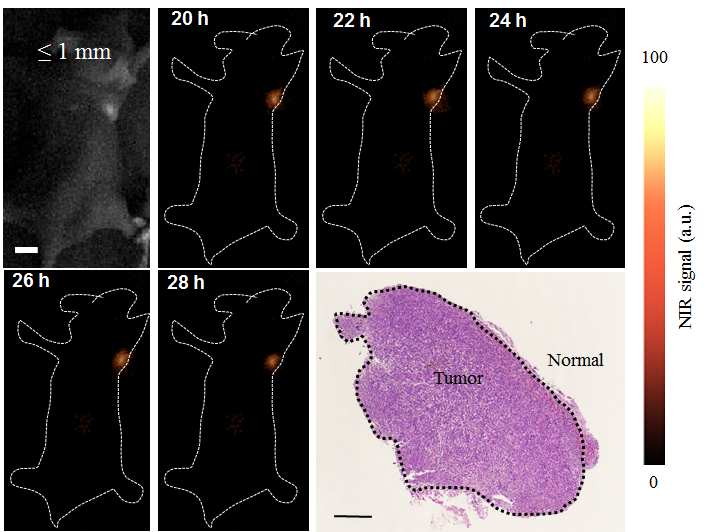


**Supplementary Figure 35.** NIR-II fluorescence bioimaging-guided epidermal tumor surgical resection (8 days after CaOV_3_ cells subcutaneous injection). The resection was operated in the optimal surgical time window (22 h PI of 1^st^ injection) (Scale bar, 5 mm). Tumor margin was confirmed by the H & E staining result and tumor size was ≤ 1 mm (Scale bar, 0.1 mm). Representative images are for n = 5.


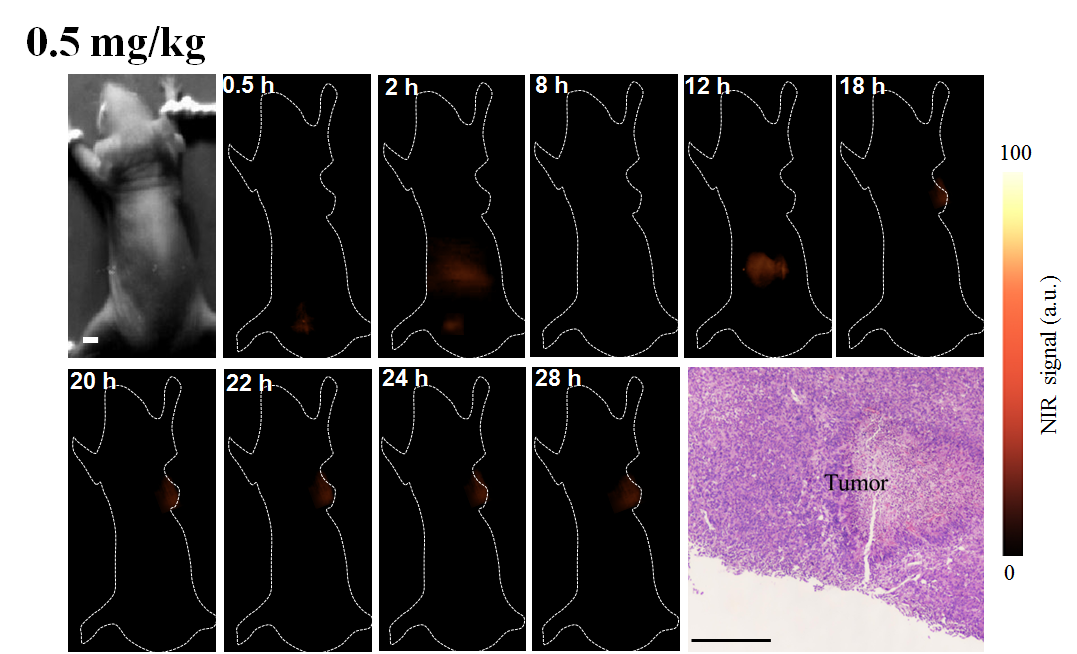


**Supplementary Figure 36.** NIR-II fluorescence image-guided epidermal tumor surgical resection with DCNPs-L_1_-FSH_β_ injection dose of 0.5 mg kg^-1^. The resection was operated in the optimal surgical time window (22 h PI of 1^st^ injection) (Scale bar, 5 mm). Tumor margin was confirmed by the H & E staining result (Scale bar, 0.2 mm). Representative images are for n = 5.


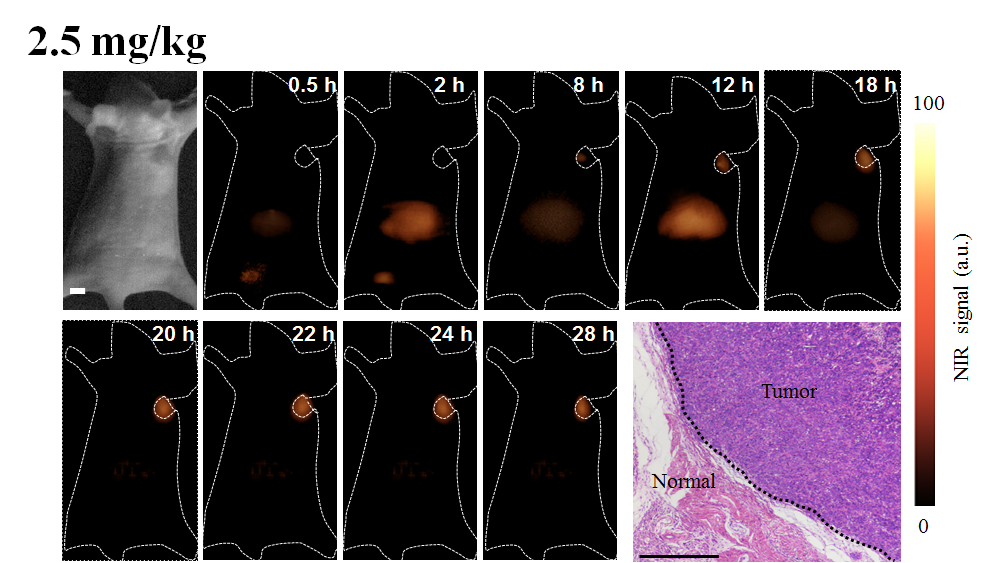


**Supplementary Figure 37.** NIR-II fluorescence image-guided epidermal tumor surgical resection with DCNPs-L_1_-FSH_β_ injection dose of 2.5 mg kg^-1^. The resection was operated in the optimal surgical time window (22 h PI of 1^st^ injection) (Scale bar, 5 mm). Tumor margin was confirmed by the H & E staining result (Scale bar, 0.2 mm). Representative images are for n = 5.


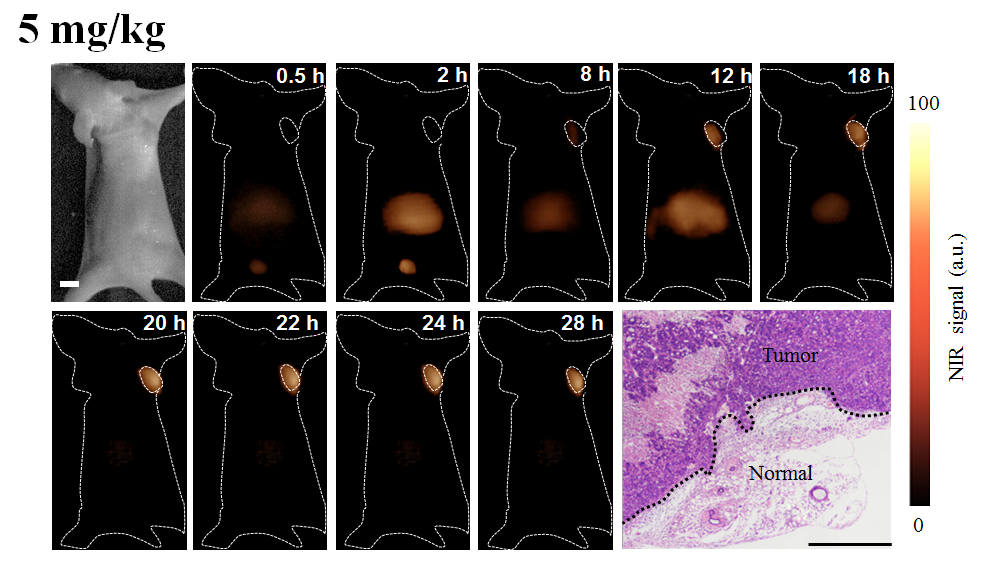


**Supplementary Figure 38.** NIR-II fluorescence image-guided epidermal tumor surgical resection with DCNPs-L_1_-FSH_β_ injection dose of 5 mg kg^-1^. The resection was operated in the optimal surgical time window (22 h PI of 1^st^ injection) (Scale bar, 5 mm). Tumor margin was confirmed by the H & E staining result (Scale bar, 0.2 mm). Representative images are for n = 5.


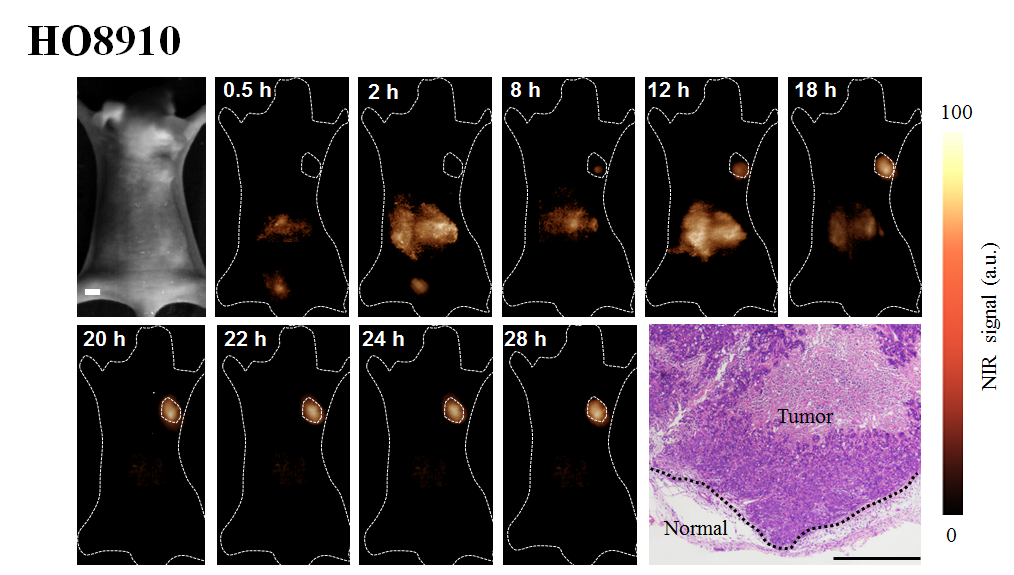


**Supplementary Figure 39.** NIR-II fluorescence image-guided surgery for HO8910 epidermal ovarian tumor model (21 days after HO8910 cells subcutaneous injection). The resection was operated in the optimal surgical time window (22 h PI of 1^st^ injection) (Scale bar, 5 mm). Tumor margin was confirmed by the H & E staining result (Scale bar, 0.2 mm). Representative images are for n = 5.


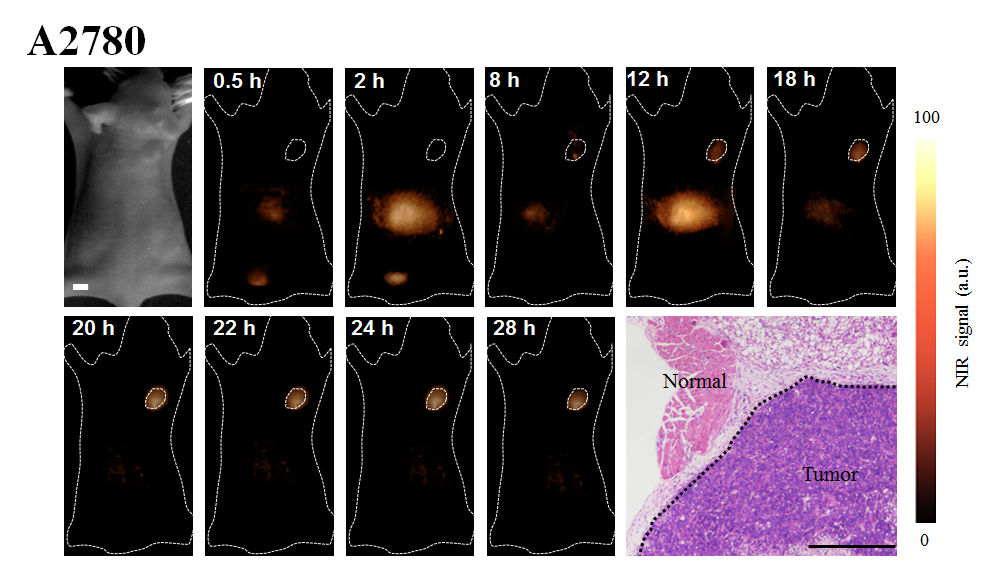


**Supplementary Figure 40.** NIR-II fluorescence image-guided surgery for A2780 epidermal ovarian tumor model (21 days after A2780 cells subcutaneous injection). The resection was operated in the optimal surgical time window (22 h PI of 1^st^ injection) (Scale bar, 5 mm). Tumor margin was confirmed by the H & E staining result (Scale bar, 0.2 mm). Representative images are for n = 5.


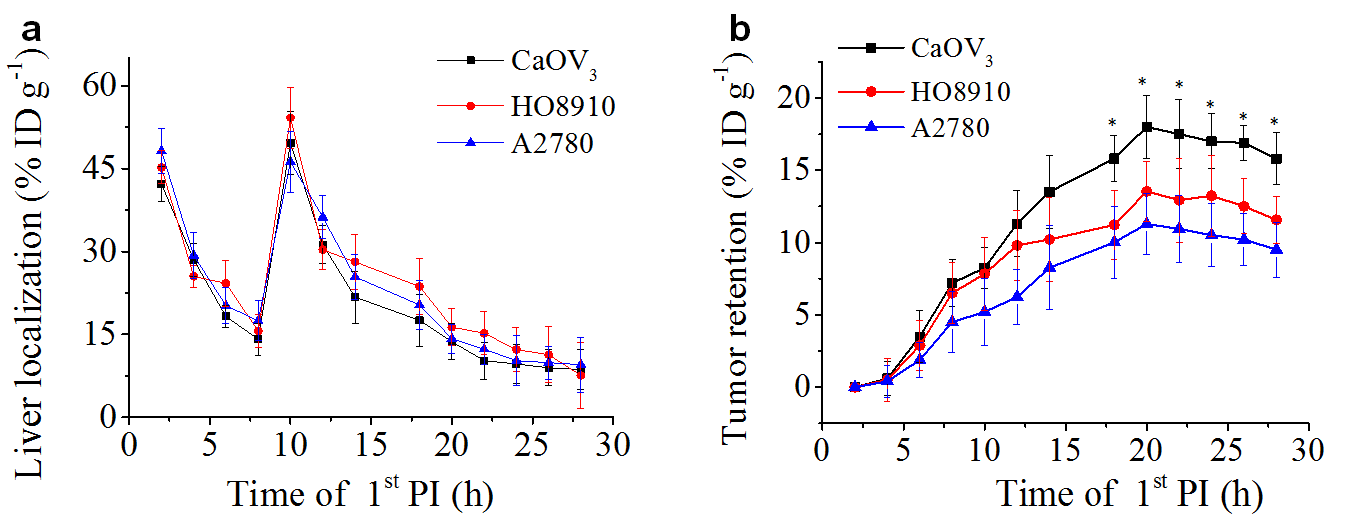


**Supplementary Figure 41.** Liver localization (**a**) and tumor retention (**b**) of nanorprobes in our epidermal tumor model (CaOV_3_ cell line) and other two kinds of epidermal ovarian tumors models (HO8910 and A2780 cell lines) which were analyzed by ICP-MS. The 2^nd^ injection of HO8910 and A2780 epidermal ovarian tumors-bearing mice were administrated by tail injection at 8 h PI of 1^st^ injection which was the same as the CaOV_3_ epidermal tumor. Mean ± s.d. for n = 5 (*P < 0.05 versus the HO8910 epidermal tumor model, two-sided Student^’^s t-test).


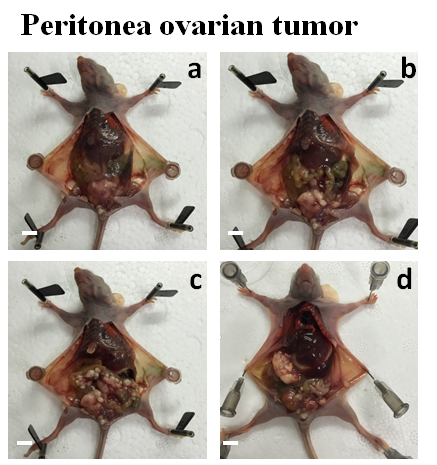


**Supplementary Figure 42.** Optical photos of peritoneal carcinomatosis. (**a**) No tumors can be observed before the peritoneum opening. (**b**) After the peritoneum was opened, several metastatic tumors can be observed. (**c**) Lots of metastatic lesions were observd under the intestines and reticuloendothelial system organs. (**d**) Another nude mice with peritoneal carcinomatosis. Scale bars, 1 cm.


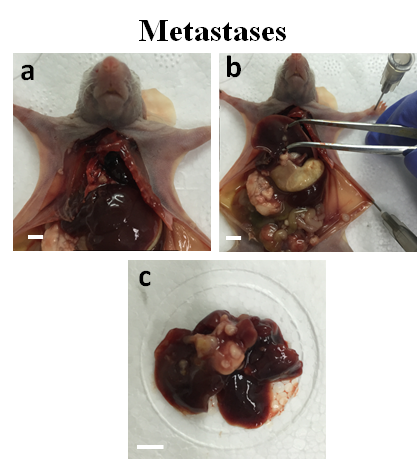


**Supplementary Figure 43.** Optical photos of metastatic ovarian tumor. (**a**) No tumors can be observed before the peritoneal opening. (**b**) Tumors were observed under the liver. (**c**) *Ex-vivo* optical image of tumors which hided under the liver. Scale bars, 5 mm.

**
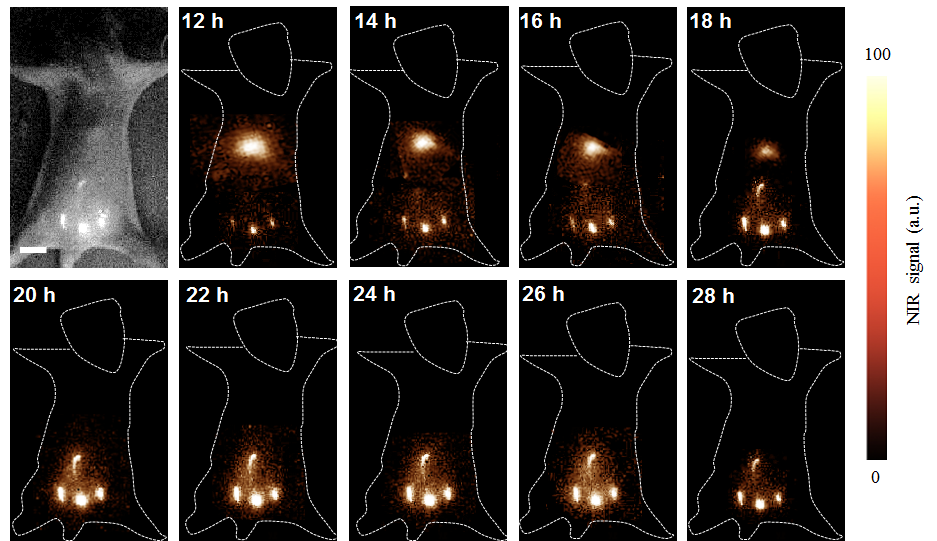
**

**Supplementary Figure 44.** NIR-II fluorescence bioimaging results in human ovarian adenocarcinoma peritoneal metastases model**.** The 2^nd^ injection was administrated by tail injection at 8 h PI of 1^st^ injection which was the same as the murine epidermal tumor. Scale bar, 1 cm. Representative images are for n = 5.


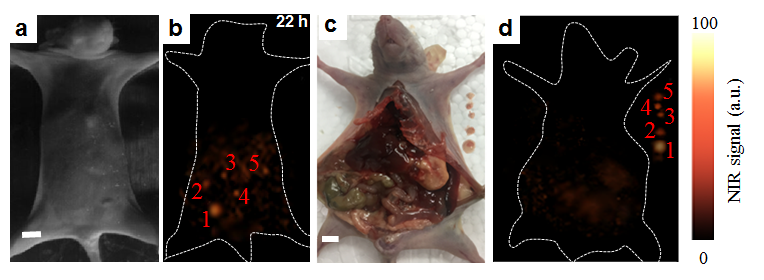


**Supplementary Figure 45.** The NIR-II fluorescence bioimaging result (22 h PI of 1^st^ injection) of peritoneal metastases (**a**, **b**), digital image (**c**) and NIR-II fluorescence bioimaging result (**d**) after the surgical resection. Fluence rates, b = 40 mW cm^-2^ and d = 25 mW cm^-2^. All scale bars, 5 mm. Representative images are for n = 5.


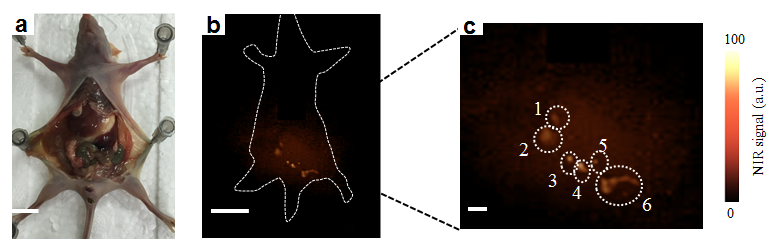


**Supplementary Figure 46.** Optical photo (**a**) and corresponding NIR-II fluorescence bioimaging result (**b**) of early abdominal tumor in the optimal surgical time window (at 22 h PI of 1^st^ injection). (**c**) Enlargement of NIR-II fluorescence image of early lesions in (b). Scale bars, 1 cm in a and b, 5 mm in c. Representative images are for n = 5.


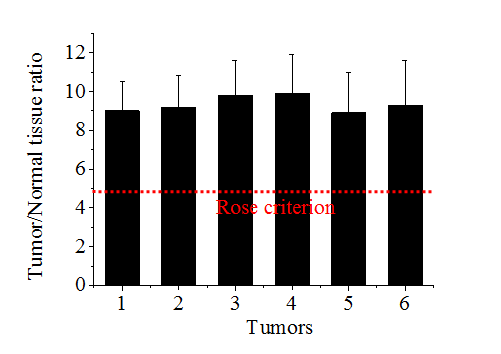


**Supplementary Figure 47.** T/L ratios of No. 1 ~ 6 early metastatic lesions obtained by NIR-II fluorescence bioimaging in Supplementary Figure 46c. The red dotted line refers to Rose criterion. All T/N ratios were higher than Rose criterion, indicating the efficient early metastatic lesions discrimination. The T/N ratio is tumor-to-normal peripheral tissue ratio in this peritoneal metastases model experiments. Mean ± s.d. for n = 5.


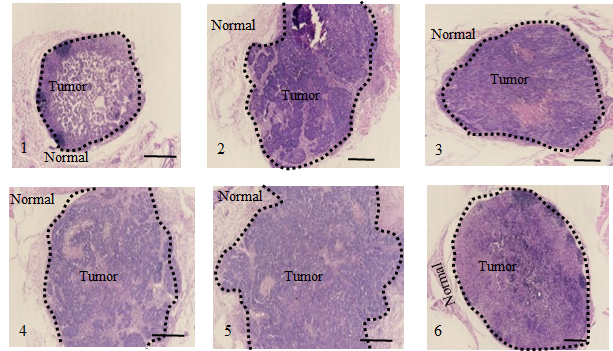


**Supplementary Figure 48.** H & E staining of Nos. 1 ~ 6 early lesions which were surgical resected under NIR-II fluorescence bioimaging guidance in Supplementary Figure 46c. All border lines between early metastatic lesions and normal tissues can be observed, suggesting subsequent effectively surgical resection in the optimal surgical time window. All the resected fluorescent metastases were confirmed to be malignant. All scale bars, 0.2 mm. Representative images are for n = 5.


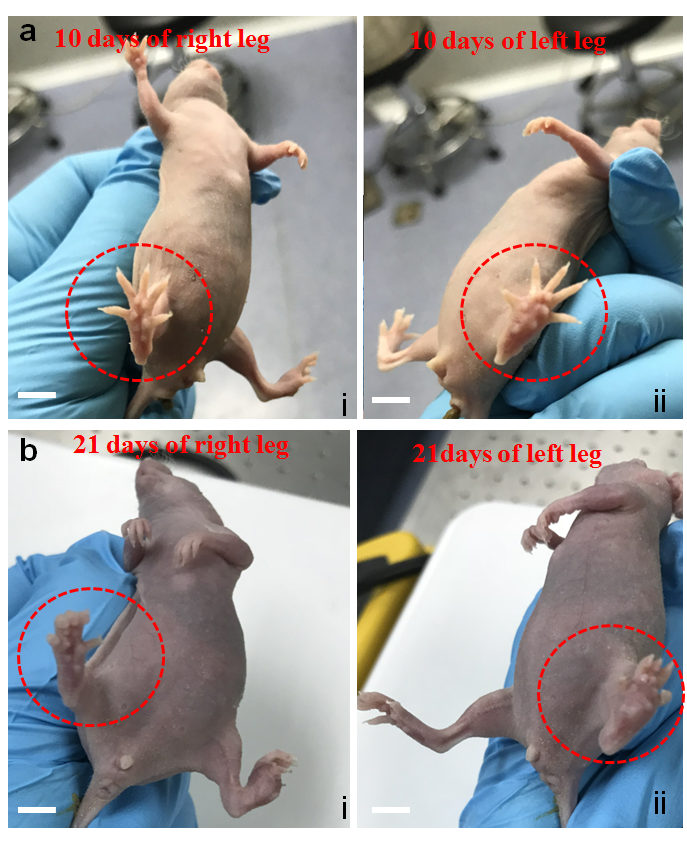


**Supplementary Figure 49.** A popliteal lymph node metastasis model by intradermal injected CaOV_3_ cell (5 *10^8^ cells mouse^-1^) to the hind paw of five-week-old mice for 3 weeks. Digital images of mice with normal right legs (**ai**, **bi**) and left legs with intradermal hind paw injection of CaOV_3_ cells after 10 days (**aii**) and 21 days (**bii**). Scale bars, 1 cm.


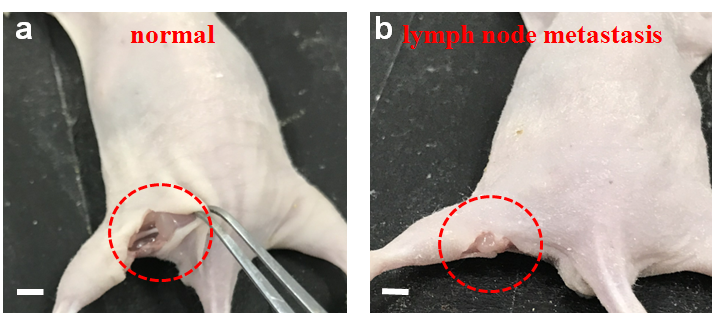


**Supplementary Figure 50.** Digital images of normal (**a**) and popliteal lymph node metastasis-bearing mice (**b**). Scale bars, 5 mm.

**
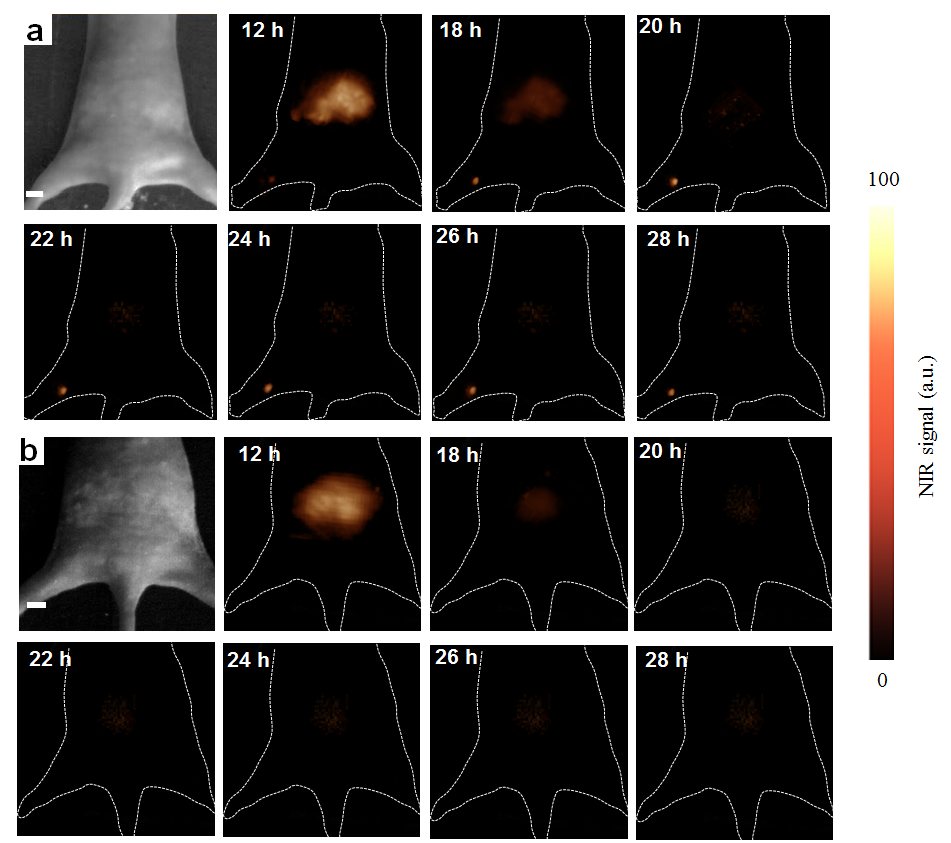
**

**Supplementary Figure 51.** NIR-II fluorescence bioimaging of popliteal lymph node metastasis model (**a**) and normal popliteal lymph node (**b**). The 2^nd^ injection was administrated by tail injection at 8 h PI of 1^st^ injection which was the same as the murine epidermal tumor. Compared with the normal lymph node, lymph node metastasis can be successfully delineated by NIR-II fluorescence bioimaging in the optimal surgical time window. Scale bars, 5 mm. Representative images are for n = 5 per group.


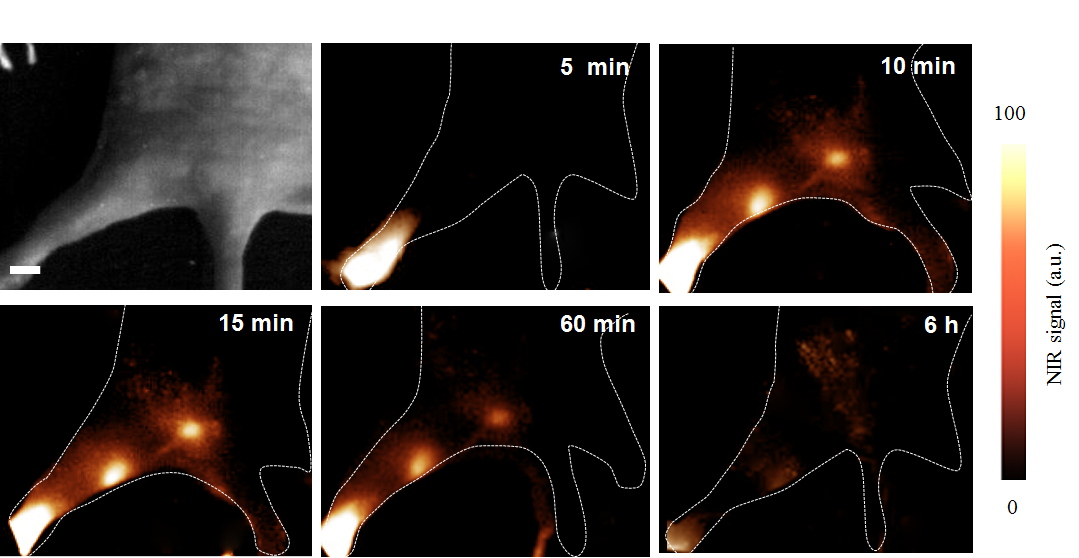


**Supplementary Figure 52.** NIR-II fluorescence bioimaging of normal lymph node after intradermal administrated the 1^st^ injection at the dorsal skin of the left hind paw. The popliteal lymph node metastasis cannot be observed by traditional intradermal hind paw injection in popliteal lymph node metastasis model. Scale bar, 5 mm. Representative images are for n = 5.


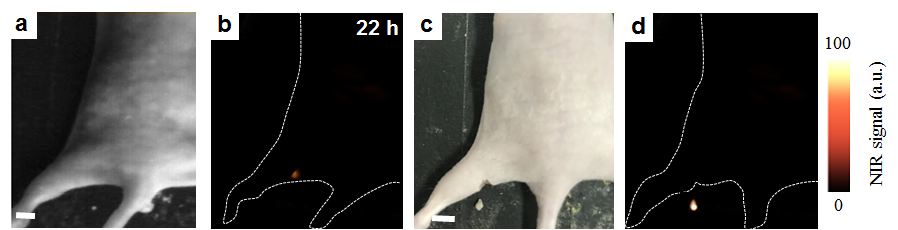


**Supplementary Figure 53.** The NIR-II bioimaging results (22 h PI of 1^st^ injection) of popliteal lymph node metastasis (**a**, **b**). Digital image (**c**) and NIR-II fluorescence bioimaging result (**d**) after surgical resection. All scale bars, 5 mm. Representative images are for n = 5.

**
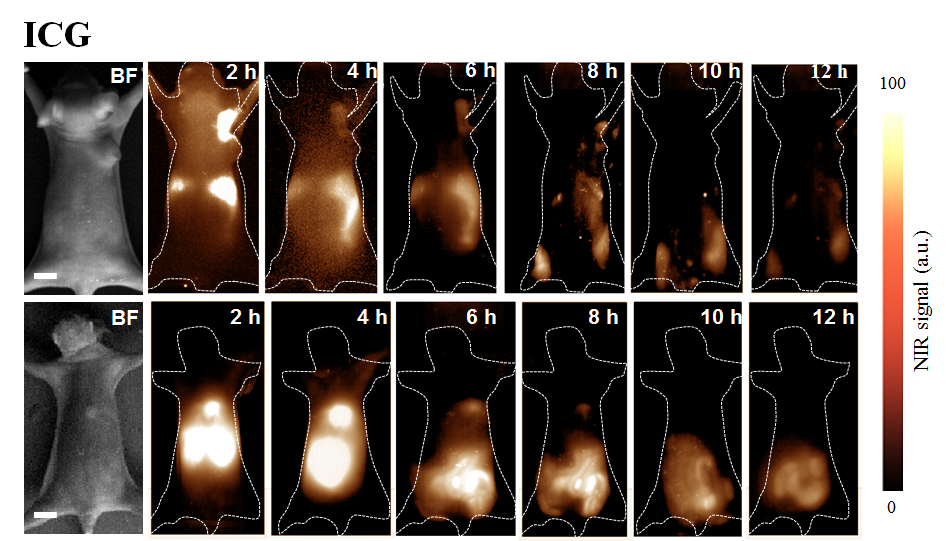
**

**Supplementary Figure 54.** NIR-II fluorescence bioimaging results of subcutaneous tumor bearing mice after tail injection with ICG probes for various hours. The upper and bottom rows refer to the backside and abdomen of the mice respectively. ICG were severely accumulated in organs of the reticuloendothelial system and intensities, resulting in high background signals. All scale bars, 1 cm. Representative images are for n = 5.


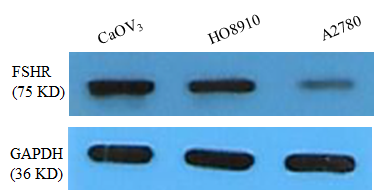


**Supplementary Figure 55.** The uncropped blots of FSHR expression in three different cell lines, CaOV_3_, HO8910 and A2780 measured by WB method.


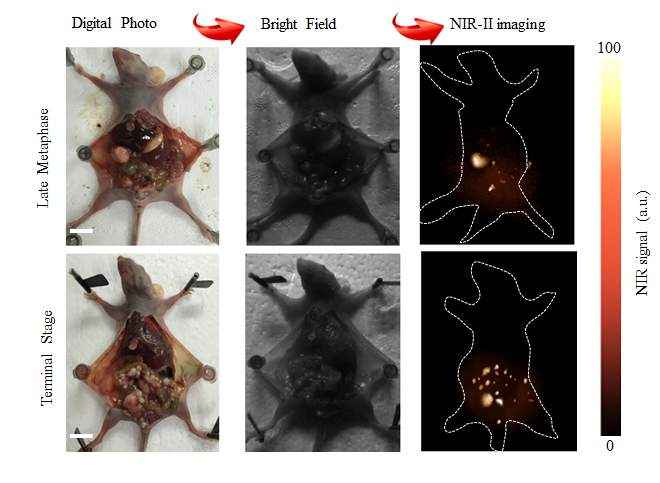


**Supplementary Figure 56.** Optical images, bright field images and NIR-II fluorescence bioimaging results of late metaphase and terminal stage of peritoneal ovarian metastases. The NIR-II bioimaging results were obtained at 22 h PI of 1^st^ injection (2^nd^ injection was tail injected at 8 h PI of the 1^st^ injection). The large tumor margins and eye-invisible lesions can be successful observed. All scale bars, 1 cm. Representative images are for n = 5.
