# Supplementary material for: NIR-II nanoprobes in-vivo assembly to improve image-guided surgery for metastatic ovarian cancer
Source: Nat Commun. 2018 Jul 24;9:2898. doi: 10.1038/s41467-018-05113-8 (PMC6057964; doi:10.1038/s41467-018-05113-8)
Supplement: Supplementary file 2 — Description of Additional Supplementary Files [file 41467_2018_5113_MOESM2_ESM.pdf]

Reviewers' comments:

Reviewer #1 (Remarks to the Author):

Real-time fluorescence imaging in the NIR II region offers noninvasive detection of tumors and their image-guided surgery. Especially, monitoring of metastatic cancers and residual small nodules is of significant importance for improving prognosis of cancer patients This is well designed and presented data are solid, but enthusiasm for acceptance is tempered by lacking some critical experimental data, as detailed below, which should be addressed prior to making a decision.

1) Dai et al. have already published many papers in this field (Refs. 23-29). This study should be compared with those published work to show the novelty of this study with the advantage of presented DSNPs.
2) The characterization of the nanoprobes in this study needs to be improved. Especially, the size analysis of nanoprobes needs to be conducted in serum containing media to predict their functional behaviors in cells and animals (Fig. S2). Optical properties also need to be measured in warm serum (Fig. 2). This is especially important since these physicochemical properties are important parameters mediating the internalization of nanocrystals into living cells and tissues.
3) Solubility of these nanoprobes in aqueous solutions is extremely important to translate them into the clinic. Authors need to describe current trials of surface modifications to improve water solubility and to avoid quenching in aqueous solutions.
4) What’s the working distance used for the intraoperative imaging? Fluence rate is 0.2 W/cm2, but the laser exposure to the FOV is dependent on the distance between the subject and excitation source.
5) Also authors need to address that the fluence rate under the guideline of FDA for clinical use.
6) Injection dose used in this study (7.5 mg/kg) is quite high, compared to the clinical dose of ICG. Dose optimization should be performed, and displayed in the main text.
7) Why two-step injections? This strategy improved tumor targeting efficacy, but increased clinical concerns in terms of immune responses and adverse effects.
8) Tumor uptake and retention were well quantified in this study. In many figures, tumor to normal tissue ratio was displayed, but it isn’t clear what’s the normal tissue used to compare. Tumor-to-liver ratio is an important indicator for tumor targeting, the comparison should be against the liver.
9) Scale bars are missing in Fig. 3a,e, Fig. 4a,b, and Fig. 5a.
10) Blood curve and urinary excretion of 1st injection is not displayed.
11) Show resected tumors with color and fluorescence images so that readers can see the correlated size and intensity of each tumor.
12) Histology in Fig 5c confirms the resected tumors. This should be also taken under the fluorescence microscope to confirm the NIR signals in the tumoral tissue.
13) Also if this is really in the tumor site, it should be confirmed whether the signals come from the tumor cell or interstitial space, which will clearly support the tumor targeting concept of this study.

Reviewer #2 (Remarks to the Author):

In this manuscript, the authors describe near-infrared II nanoparticles for detection of disseminated ovarian cancer. The particles were constructed using layer-by-layer methodology and included an FSH targeting peptide. An important part of these particles is in vivo assembly – the authors used a two-stage injection approach whereby particles were injected at separate time points (8h being the optimum separation), with the second injection containing DNA complementary to the first, allowing assembly via Watson-Crick base-pairing. This strategy was significantly better that using preformed nanoparticles. Similarly, FSH gave better results than an integrin-binding RGD motif.

The authors performed two in vivo surgical experiments – one with a subcutaneous deposit, the other with intraperitoneal deposits, reflecting the dissemination pattern of advanced high grade serous ovarian cancer, which is the critical target patient population in ovarian cancer management.

Overall, the experiments have been performed well, with suitable controls. This reviewer is an ovarian cancer researcher rather than a polymer chemist – for the non-specialist reader, the details of the nanoparticle construction were not entirely clear, especially the difference between DSNP-L1-FSH and DSNP-L2-FSH.

There are three main comments
1. Originality. In both the introduction and the discussion, the authors make almost no mention of previous work in this field. There are several recent publications both on layer-by-layer nanoparticle construction and the use of NIR-II fluorescence specifically in ovarian cancer (e.g. see PMID 28928826, 28482280, 27114520) – these should be mentioned and compared to the data in this manuscript. Although the two-stage procedure is novel, this reviewer cannot see that this work represents a major advance in this field.
2. The use of a single xenograft model. Although the work presented here is about proof-of-methodology, it would be more impressive if a second model were utilised. This also raised the issue of FSH as the target peptide. FSH is not commonly assessed as an immunohistochemistry marker in high grade serous carcinoma – what proportion of these tumours express FSH at appropriate levels?
3. Downstream application. Is advanced ovarian cancer the correct setting in which to apply this technology? Experienced ovarian cancer surgeons can already remove all visible disease through extensive surgical effort (stripping diaphragmatic peritoneum etc) – and yet 80+% of women with advanced high grade serous carcinoma still relapse. The recent LION study indicated that the additional resection of macroscopically normal nodes in addition to all visible macroscopic disease made no impact upon progression/recurrence. Thus, I am struggling somewhat to see how this technology might be used in ovarian cancer. Other uses (e.g. detection of sentinel LN, resection margins within the liver or brain) might be more immediately useful.

Minor issues
1. Line 107. “5 %Nd@NaGdF4” - what does this actually mean?
2. Figure 1 image: “Time interval with 8h” should be “Time interval of 8h”.
3. Fig. 2f – was this experiment conducted at 37oC?
4. Fig. S5 – I think that there is a mistake – think the red figure legend label should be DSNPs-L1-FSH + DSNPs-L2-FSH.
5. Fig. S6 – no error bars. How many mice were these experiments performed in? Please add error bars and state N in legend.
6. Lines 144 and 150 – the word ‘drastically’ is a little dramatic – ‘greatly’, ‘markedly’ or even ‘significantly’ are more appropriate.

Point-by-Point Response to Referees

Reviewers' comments:

Reviewer #1 (Remarks to the Author):

Real-time fluorescence imaging in the NIR-II region offers noninvasive detection of tumors and their image-guided surgery. Especially, monitoring of metastatic cancers and residual small nodules is of significant importance for improving prognosis of cancer patients. This is well designed and presented data are solid, but enthusiasm for acceptance is tempered by lacking some critical experimental data, as detailed below, which should be addressed prior to making a decision.

Comments:

1. ***Dai et al. have already published many papers in this field (Refs. 23-29). This study should be compared with those published work to show the novelty of this study with the advantage of presented DSNPs.***

Response: Thanks for the comments.

In order to make this point clearer for the readers, we have added the following sentences in the introduction part of the revised mauscript (on pages 3-4, lines 68-75):

"Second-window near-infrared fluorescence (NIR-II, 900–1700 nm) probes such as single-walled carbon nanotubes (SWNTs)[23,24], quantum dots (QDs)[25,26], lanthanide-based downshifting nanoparticles (DSNPs)[27-29] and organic dyes[30-33] are promising for *in vivo* fluorescence imaging due to sub-10-μm high-resolution imaging at a few centimeters tissue penetration depth and low tissue autofluorescence[23-33], which are promising candidates for both preoperative imaging and intraoperative reality[28]. Especially, DSNPs play a very important role in the NIR-II fluorescent bioimaging applications due to their distinct properties, such as highly controlled particle size, non-photobleaching, long lifetime and high efficiency optical properties[27-29]."

We have also added the following scentences in the disscusion part of the revised mauscript (on pages 13-14, lines 283-300):

"Thus far, NIR-II contrast agents including SWNTs[23,24], QDs[25,26], DSNPs[27-29] and organic dyes[30-33] have been established for *in vivo* imaging of vascular flow, lymphatic and tumors. Although the organic dyes shows great potentials to facilitate FDA approval and clinical translation, these small molecule probes typically experienced short time tumor retention because of their rapid clearance, which hindered the following precision imaging guided resection. For the inorganic SWNTs, QDs and DSNPs NIR-II probes, the long term cytotoxicity is still a major concern. One way to overcome these problems is to design nanoprobes with sizes smaller than 10 nm, i.e., below what is believed to be the threshold for renal clearance[56,57]. Since the well controllable particle size and superior optical properties including non-photobleaching, long lifetime and high efficiency, DSNPs NIR-II probes have attracted a great of attentions for the *in vivo* bioimaging recently. For example, early ovarian tumor detection was explored by using

lanthanide DSNPs (Er^{3+} and Ho^{3+} doped)[39,58] and even multi-organ cancer metastases could be successfully visualized (Er^{3+} doped)[59]. Unfortunately, all current in vivo bioimaging strategy for imaging-guided surgery with DSNPs NIR-II nanoprobes are still limited by the serious RES accumulation and short time tumor retention[28,29]. In the present work, we have developed a novel *in vivo* self-assembly bioimaging strategy to realize the rapid whole body clearance and long tumor retention nanoprobes and then utilized the NIR-II fluorescence bioimaging for efficient tumor surgery."

We have cited the following reference in the revised manuscript (on pages 23-24, lines 508-516, page 24, lines 533-535, page 26, lines 569-571, page 27, 582-592):

"(31) Li, B., Lu, L., Zhao, M., Lei, Z. & Zhang, F. Efficient 1064-nm NIR-II excitation fluorescent molecular dye for deep-tissue high-resolution dynamic bioimaging**.** *Angew. Chem. Int. Ed.* https://doi.org/10.1002/anie.201801226 (2018).

(32) Qi, J., Sun, C., Zebibula, A., Zhang, H., Kwok, R. T. K., Zhao, X., Xi, W., Lam, J. W. Y., Qian, J. & Tang, B. Z. Real-time and high-resolution bioimaging with bright aggregation-induced emission dots in short-wave infrared region. *Adv. Mater.* https://doi.org/10.1002/adma.201706856 (2018).

(33) Cosco, E. D., Caram, J. R., Bruns, O. T., Franke, D., Day, R. A., Farr, E. P., Bawendi, M. G. & Sletten, E. M. Flavylium polymethine fluorophores for imaging in the near- and shortwave infrared. *Angew. Chem. Int. Ed.* **56**, 13126-13129 (2017).

(39) Dang, X. N., Gu, L., Qi, J., Correa, S., Zhang, G., Belcher, A. M. & Hammond, P. T. Layer-by-layer assembled fluorescent probes in the second near-infrared window for systemic delivery and detection of ovarian cancer. *Proc. Natl. Acad. Sci. USA* **13**, 5179-5184 (2016).

(51) Pu, T., Xiong, L., Liu, Q., Zhang, M., Cai, Q., Liu, H., Sood, A. K., Li, G., Kang, Y. & Xu, C. Delineation of retroperitoneal metastatic lymph nodes in ovarian cancer with near-infrared fluorescence imaging. *Oncol. Lett.* **14**, 2869-2877 (2017).

(56) Peer, D., Karp, J. M., Hong, S., Farokhzad, O. C., Margalit, R. & Langer, R. Nanocarriers as an emerging platform for cancer therapy. *Nat. Nanotechnol.* **2**, 751-760 (2007).

(57) Ma, K., Sai, H. & Wiesner, U. Ultrasmall sub-10 nm near-infrared fluorescent mesoporous silica nanoparticles. *J. Am. Chem. Soc.* **134**, 13180-13183 (2012).

(58) Tao, Z., Dang, X., Huang, X., Muzumdar, M. D., Xu, E. S., Bardhan, N. M., Song, H., Wuckoff, J., Birrer, M. J., Belcher, A. M. & Ghoroghchian, P. P. Early tumor detection afforded by *in vivo* imaging of near-infrared II fluorescence. *Biomaterials*, **134**, 202-215 (2017).

(59) Kantanneni, H., Zevon, M., Donzanti, M. J., Zhao, X., Shen, Y., Barkund, S. R., McCabe, L. H., Petrosky, W. B., Higgins, L. M., Ganesan, S., Riman, R. E., Roth, C. M., Tan, M. C., Pierce, M. C., Ganapathy, V. & Moghe, P. V. Surveillance nanotechnology for multi-organ cancer metastases. *Nat. Biomed. Eng.* **1**, 993-1003 (2017)."

2. ***The characterization of the nanoprobes in this study needs to be improved. Especially, the size analysis of nanoprobes needs to be conducted in serum containing media to predict their functional behaviors in cells and animals (Fig. S2). Optical properties also need to be measured in warm serum (Fig. 2). This is especially important since these physicochemical properties are important parameters mediating the internalization of nanocrystals into living cells and tissues.***

Response: Thanks so much for the useful comments and suggestions. We have studied the

stabilities of the nanoprobes in water, PBS, blood and serum at 37 °C by the dynamic light scattering (DLS) and Fluorescence Spectrometry (FS). As shown in Fig. S5 in the revised supporting information and Fig.2f in the revised manuscript, the particle size distribution and fluorescent intensity were almost invariable in different solution at 37 °C, indicating their good stabilities in the physiological environment. Furthermore, we also studied the stability of the assembled nanoprobes in serum under the continuous irradiation with 808 nm laser (clinical approved fluence rate 40 mW/cm^2). As shown in the Fig.S7 in the revised supporting information, the dynamic light scattering results showed that the hydrodynamic diameters of nanoclusters were almost invariable under constant laser irradiation, suggesting that the assembled DSNPs were very stable in tumor site during the bioimaging process.

We have added the following contents in the revised manuscript (on page 6, lines 118-120): "These nanoprobes possessed superior aqueous solubility, stability and sustainable emission fluorescence in water and different biological buffers at 37 °C (Figs. 2f and S3-5)."

We have added the following sentence in the revised manuscript (on page 6, lines 124-128): "Due to the huge potentials of complementary nature of DNA hybridization, the observed hydrodynamic diameters of nanoclusters in serum at 37 °C were stable over long periods of study (~ 8 h) under a continuous-wave 808 nm laser irradiation (laser output power density = 0.2 W/cm^2, fluence rate 40 mW/cm^2, working distance = 30 cm[46]) (Fig. S6, S7), suggesting the assembled DSNPs were very stable in tumor site during the bioimaging process."

We have revised Fig. 2f in the revised manuscript as following:

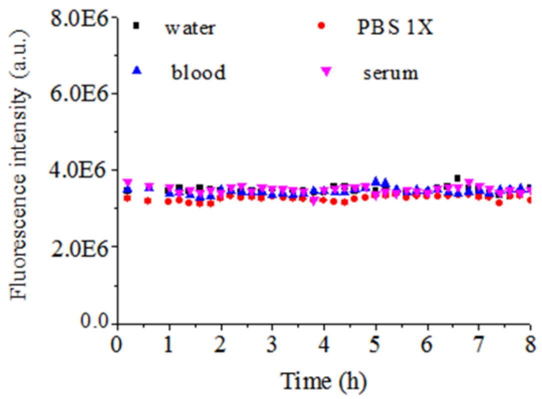

Fig. 2f Photostability of DSNPs in a variety of biological buffers at 37 °C under continuous 808 nm laser exposure at a power density of 0.2 W/cm^2.

We have added Fig. S5 in the revised supporting information as following:

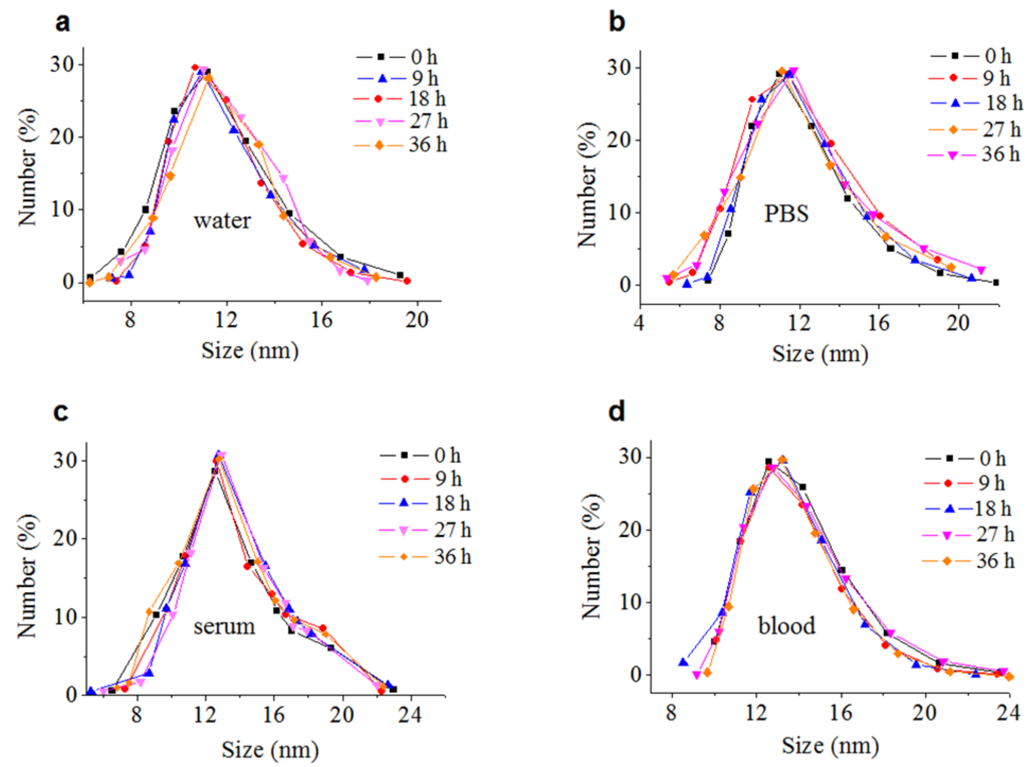

Fig. S5. Size distribution of DSNPs-L_1-FSH_β in water (**a**), PBS (**b**), serum (**c**) and blood (**d**) at 37 °C for various time.

We have added Fig. S7 in the revised supporting information as following:

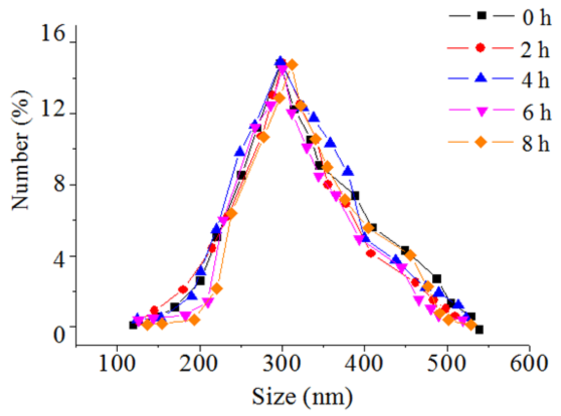

Fig. S7. Hydrodynamic diameters of nanoclusters in serum at 37 °C after 808 nm laser irradiation (laser output power density = 0.2 W/cm^2, fluence rate = 40 mW/cm^2) for various time.

3. ***Solubility of these nanoprobes in aqueous solutions is extremely important to translate them into the clinic. Authors need to describe current trials of surface modifications to improve water solubility and to avoid quenching in aqueous solutions.***

Response: We appreciate the reviewer for the useful suggestion.

To facilitate the bioapplication, the oleic acid capped DSNPs were transferred to the aqueous phase by using mono-layer of amine-phospholipids, and then DNA was modified on the surface. Finally, the targeting peptides (FSH_{β}) were linked on DSNPs by EDC/NHS reaction. The detailed experimental protocols have been listed in the revised supporting information. Actually, this surface modification approach with phospholipids has been used extensively for the quantum dots and other inorganic nanocrystals to realize superior aqueous solubility (*Science*, **1998**, *281* (5385), 2016-2018, *J. Am. Chem. Soc.*, **2011**, *133* (24), 9480−9496, *ACS Nano*, **2009**, *3* (9), 2573–2580). According to the Fluorescence Spectrometry (FS) and dynamic light scattering (DLS) results, the particle size distribution and fluorescent intensity of DSNPs were almost invariable during the surface modification process (Fig. S3 in the revised supporting information). Furthermore, since the frequently used nanoprobes doses for bioimaging is from 2.0 ~ 50 mg/kg (*Mol. Ther.*, **2009**, *17* (9), 1555-1562, *Biomaterials*, **2012**, *33* (7), 2215-2222), we studied the aqueous solubility and stability of the DSNPs with varied concentrations by DLS. The particle size distribution was almost invariable for the 2.0, 10 and 50 mg/kg DSNPs aqueous solution (Fig. S4 in the revised supporting information), further suggesting the phospholipids, DNA and peptides modifications had little effect on the solubility and stability of the nanoprobes.

In order to avoid the quenching effect for the lanthanide DSNPs nanoprobes, it's necessary to fabricate the core/shell structured nanoparticles to protect the luminescent core by coating an inert shell (*Nano Lett.*, **2014**, *14* (6), 3634–3639). To make this point clearer for the readers, we have added Fig. S1 in the revised supporting information to compare the fluorescence intensity of the $NaGdF_4$: 5 %Nd core nanocrystals and $NaGdF_4$: 5 %Nd@$NaGdF_4$ core/shell structured nanocrystals with varied shell thickness in the aqueous solution. The results showed that the quenching effect of water can be avoided after gradually coating a 2.5 nm shell.

We have added the following sentence in the revised manuscript (on page 5, lines 112-114): "The fluorescence quenching effect of water can be avoided after gradually coating a 2.5 nm inert $NaGdF_4$ shell on the 5.0 nm luminescent $NaGdF_4$: 5 %Nd cores (Fig. S1)."

We have added the following sentence in the revised manuscript (on page 6 lines 118-120): "These nanoprobes possessed superior aqueous solubility, stability and sustainable emission fluorescence in water and different biological buffers at 37 °C (Figs. 2f and S3-5)."

We have added Fig. S1 in the revised supporting information as following:

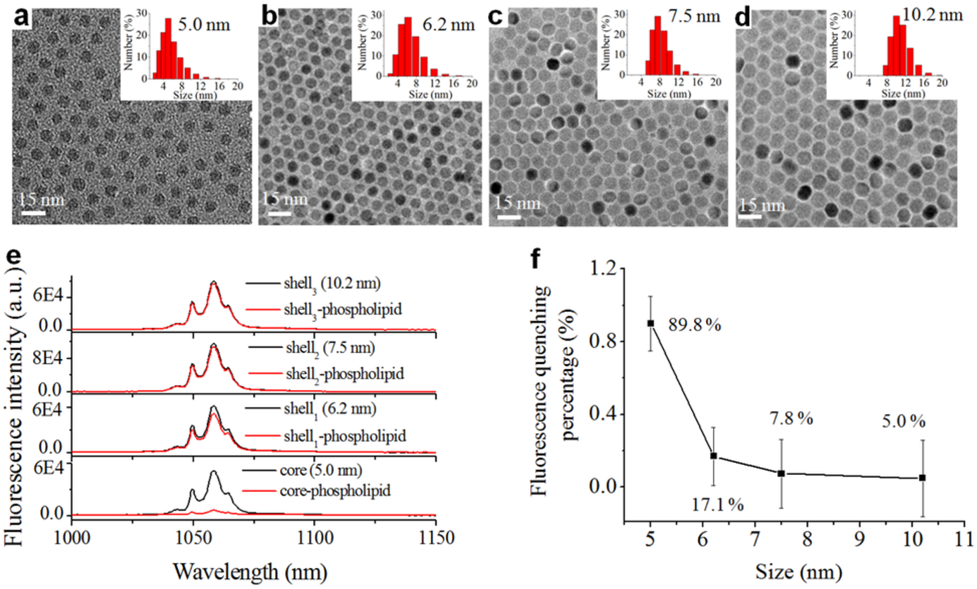

Fig. S1. TEM images of $NaGdF_4$: 5 %Nd core nanocrystals (**a**) and $NaGdF_4$: 5 %Nd@$NaGdF_4$ core-shell structured nanocrystals with different shell thickness: 1.2 nm (**b**), 2.5 nm (**c**) and 5.5 nm (**d**). Insert, dynamic laser scattering (DLS) analysis of size distribution. (**e**) Fluorescence intensity of the $NaGdF_4$: 5 %Nd core and $NaGdF_4$: 5 %Nd@$NaGdF_4$ core-shell structured nanocrystals with varied shell thickness in the aqueous solution. (**f**) Fluorescence quenching percentage of $NaGdF_4$: 5 %Nd core and $NaGdF_4$: 5 %Nd@$NaGdF_4$ core-shell structured nanocrystals with varied shell thickness in the aqueous solution. Mean ± s.d. for n = 5.

We have added Fig. S3 in the revised supporting information as following:

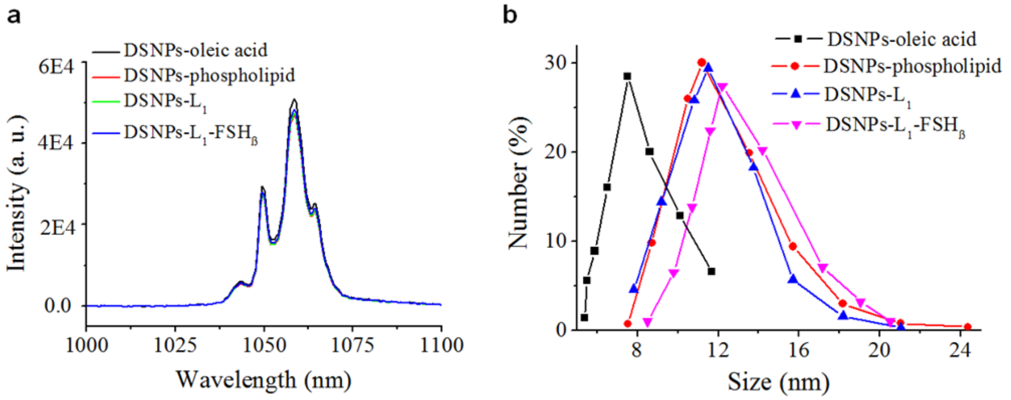

Fig. S3. (**a**) Fluorescence spectra of DSNPs-oleic acid, DSNPs-phospholipid, DSNPs-L_1 and DSNPs-L_1-$FSH_β$. **(b)** Corresponding size distribution results measured by DLS.

We have added Fig. S4 in the revised supporting information as following:

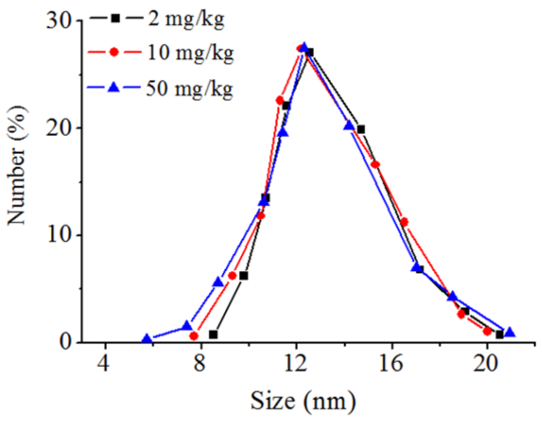

Fig. S4. DLS results for the size distribution of DSNPs-L_1-FSH_β with the frequently used nanoprobes doses in aqueous solution.

4. ***What's the working distance used for the intraoperative imaging? Fluence rate is 0.2 W/cm^2, but the laser exposure to the FOV is dependent on the distance between the subject and excitation source.***

Response: Thanks so much for the comment. In order to make this point clearer for readers, we have added the illustration (Fig. S6) of the NIR-II fluorescence bioimaging system for image-guided surgery in the revised supporting information. ~ 30 cm working distance is used for the intraoperative imaging, which is agreement with FDA approved NIR bioimaging-guided tumor surgery with ICG (*J. Surg. Oncol.*, **2011**, *104*, 323-332). The output power density of 808 nm laser is 0.2 W/cm^2 (P_1) with light spot 3.14 cm^2, and the fluence rate of the image-guided surgery platform is calculated to be 40 mW/cm^2 with light spot 78.5 cm^2.

We have added the following sentence in the revised manuscript (on page 6, lines124-128): "Due to the huge potentials of complementary nature of DNA hybridization, the observed hydrodynamic diameters of nanoclusters in serum at 37 °C were stable over long periods of study (~ 8 h) under a continuous-wave 808 nm laser irradiation (laser output power density = 0.2 W/cm^2, fluence rate 40 mW/cm^2, working distance = 30 cm[46]) (Fig. S6, S7), suggesting the assembled DSNPs were very stable in tumor site during the bioimaging process."

We have cited the following reference in the reivsed manuscript (on page 25, lines 553-557): "(46) Schaafsma, B. E., Mieog, J. S. D., Msc, M. H., van der Vorst, J. R., Kuppen, P. J. K., Löwik, C. W. G. M., Frangioni, J. V., van de Velde, C. J. H. & Vahrmeijer, A. L. The clinical use of indocyanine green as a near-infrared fluorescent contrast agent for image-guided oncologic surgery. *J. Surg. Oncol.* **104**, 323-332 (2011)."

We have added Fig. S6 in the revised supporting information as following:

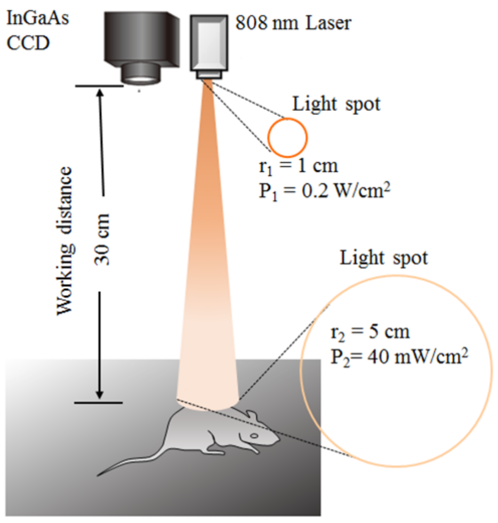

Fig. S6. Schematic illustration of the NIR-II fluorescence imaging system for tumor surgical guidance. ~ 30 cm working distance is used for the intraoperative imaging, which is agreement with FDA approved NIR bioimaging-guided tumor surgery with ICG (*J. Surg. Oncol.,* **2011**, *104*, 323-332). The output power density of 808 nm laser is 0.2 W/cm^2 (P_1) with light spot 3.14 cm^2, and the fluence rate of the image-guided surgery platform is calculated to be 40 mW/cm^2 with light spot 78.5 cm^2.

5. ***Also authors need to address that the fluence rate under the guideline of FDA for clinical use.***

Response: Thanks for the comments. The fluence rate of Clinical approval for bioimaging is ≤ 50 mW/cm^2 that tissue damage cannot occur at these fluence rates (*Cur. Opin. Chem. Biol.* **2003**, *7*, 626–634). All fluence rates in our study are 40 mW/cm^2, suggesting that the potential clinical application of the *in vivo* assembly strategy.

In order to make this point clearer for the readers, we have added the following sentences in the revised manuscript (on page 11, lines 237-243): "Finally, it's worth to mention that NIR-II fluorescence image-guided surgery was carried out with the clinical approved dose (1.5 mg/kg)[49] and fluence rate (40 mW/cm^2)[50] (Figs. 4e, S37-39). Tumor can be successfully removed under NIR-II bioimaging guidance, suggesting potential clinical application of the *in vivo* assembly strategy."

We have cited the following reference in the revised manuscript (on page 26,lines 567-568): "(50) Frangioni, J. V. *In vivo* near-infrared fluorescence imaging. *Cur. Opin. Chem. Biol.* **7**, 626–634 (2003)."

6. ***Injection dose used in this study (7.5 mg/kg) is quite high, compared to the clinical dose of ICG. Dose optimization should be performed, and displayed in the main text.***

Response: We appreciate the reviewer for the useful suggestion. The clinical guided dose of FDA approved ICG probe is 1.5 mg/kg (*Sci. Transl. Med.*, **2011**, *3* (84), 84-85). In order to optimize the

dose of nanoprobes, 0.5, 1.5, 2.5 and 5 mg/kg of nanoprobes were investigated for image-guided surgery. As shown in Fig. 4e in the revised manuscript and Figs. S37-39 in the revised supporting information, we demonstrated that the tumors can be completely resected under the guide of NIR-II bioimaging with the DSNPs dose of 1.5 mg/kg.

In order to make this point clearer for the readers, we have added the following sentences in the revised manuscript (on page 11, lines 237-243): "Finally, it's worth to mention that NIR-II fluorescence image-guided surgery was carried out with the clinical approved dose (1.5 mg/kg)[49] and fluence rate (40 mW/cm^2)[50] (Figs. 4e, S37-39). Tumor can be successfully removed under NIR-II bioimaging guidance, suggesting potential clinical application of the *in vivo* assembly strategy."

We have cited the following reference in the reivsed manuscript (on page 26, lines 564-566): "(49) Vinegoni, C., Botnaru, I., Aikawa, E., Calfon, M. A., Iwamoto, Y., Folco, E. J., Ntziachristos, V., Weissleder, R., Libby, P. & Jaffer, F. A. Indocyanine green enables near-infrared fluorescence imaging of lipid-rich, inflamed atherosclerotic plaques. *Sci. Transl. Med.* **3**, 84-85 (2011)."

We have added Fig. 4e in the revised manuscript as following:

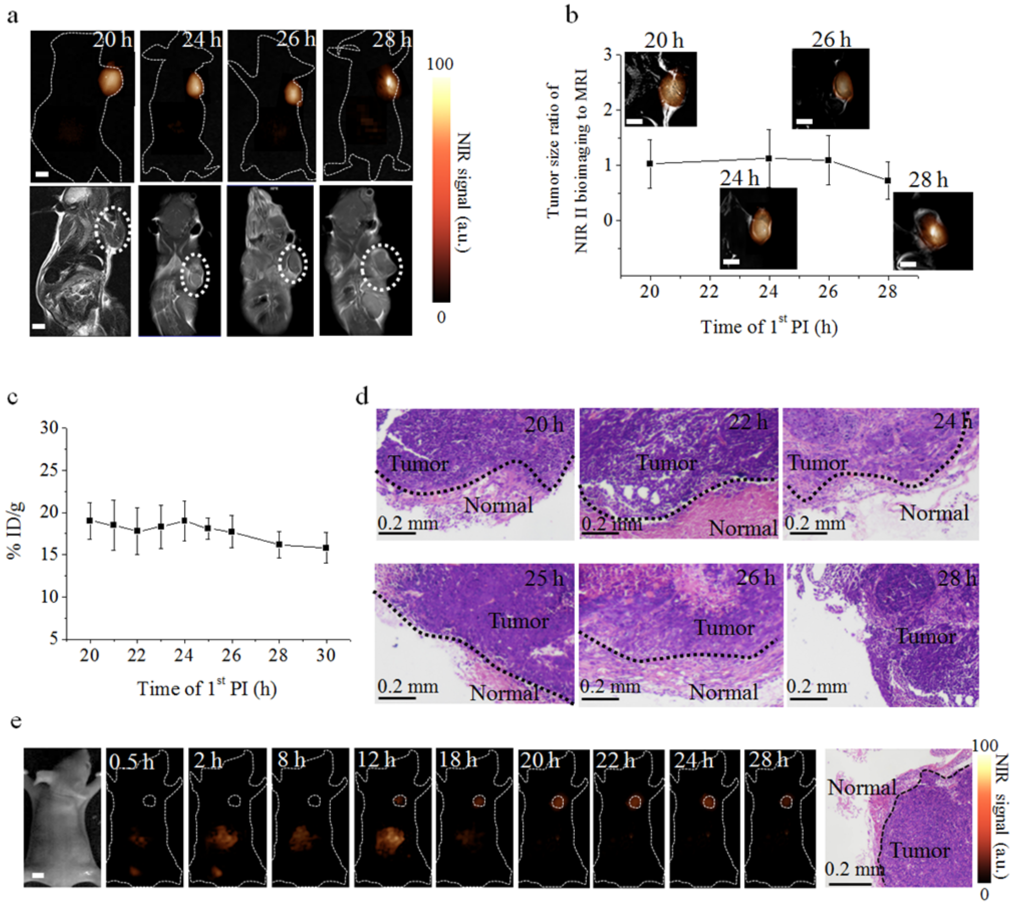

Fig. 4.| Optimal surgery time window of *in vivo* assembly. (**a**) Comparison of MRI and NIR-II fluorescence imaging (1000 nm long-pass filter) of subcutaneous human ovarian adenocarcinoma.

(**b**) Correlation for the tumor size ratio between MRI and NIR-II fluorescence bioimaging method. (**c**) Tumor retention of assembled DSNPs from 20 h to 30 h PI. (**d**) H & E staining results of the tumors resected in 20 ~ 28 h PI under NIR-II fluorescence bioimaging guidance. (**e**) NIR-II fluorescence image-guided tumor surgery with the guided dose of clinic approved ICG probe (1.5 mg/kg). Tumor margin was confirmed by the H & E staining result. All scale bars in NIR-II bioimaging and MRI results represent 5 mm. Representative images are for n = 5 per group. Mean ± s.d. for n = 5.

We have added Figs. S37-39 in the revised supporting information as following:

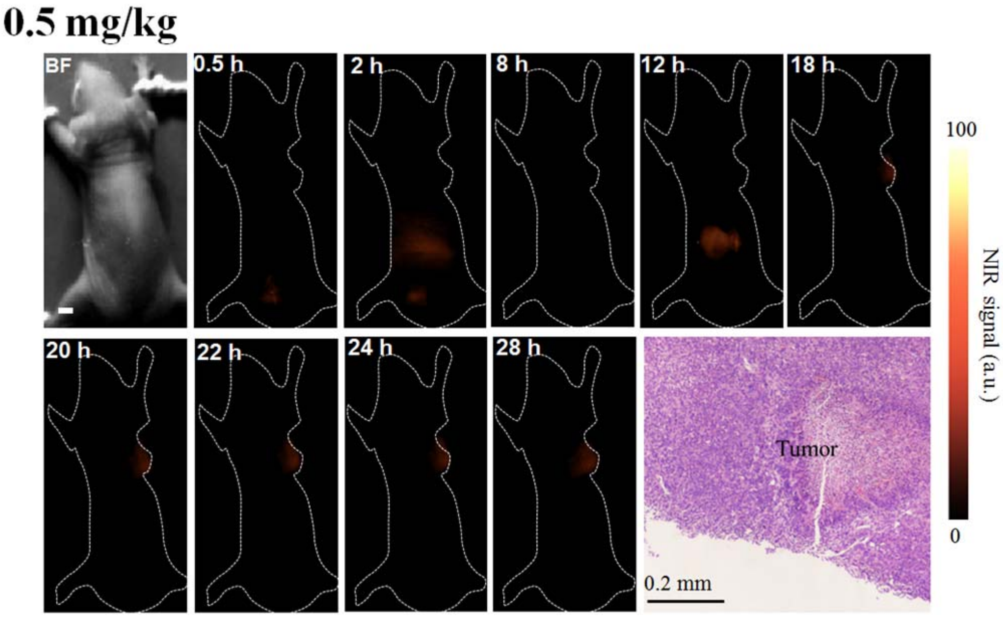

Fig. S37. NIR-II fluorescence image-guided epidermal tumor surgical resection with DSNPs-L_1-FSH_β injection dose of 0.5 mg/kg. The resection was operated in the optimal surgical time window (22 h PI of 1st injection). Tumor margin was confirmed by the H & E staining result. Scale bar represents 5 mm for the NIR-II fluorescence bioimaging results. Representative images are for n = 5.

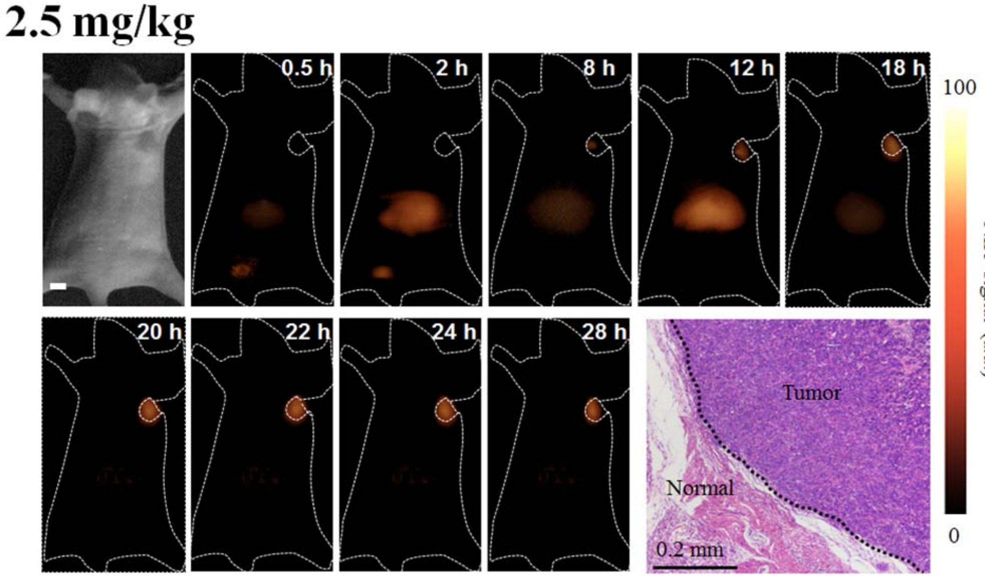

Fig. S38. NIR-II fluorescence image-guided epidermal tumor surgical resection with DSNPs-L_1-FSH_β injection dose of 2.5 mg/kg. The resection was operated in the optimal surgical time window (22 h PI of 1st injection). Tumor margin was confirmed by the H & E staining result. Scale bar represents 5 mm for the NIR-II fluorescence bioimaging results. Representative images are for n = 5.

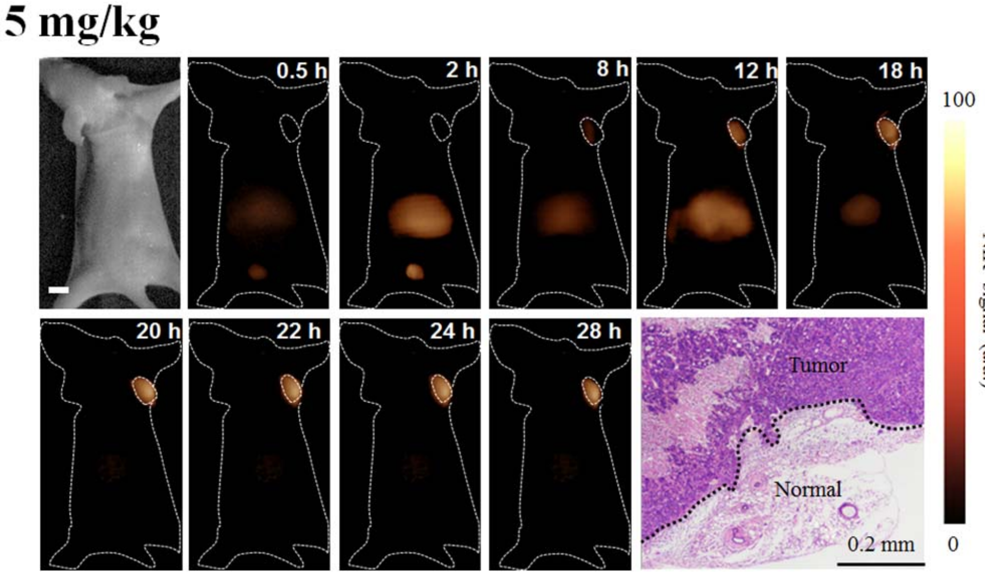

Fig. S39. NIR-II fluorescence image-guided epidermal tumor surgical resection with DSNPs-L_1-FSH_β injection dose of 5 mg/kg. The resection was operated in the optimal surgical time window (22 h PI of 1st injection). Tumor margin was confirmed by the H & E staining result. Scale bar represents 5 mm for the NIR-II fluorescence bioimaging results. Representative images are for n = 5.

7. ***Why two-step injections? This strategy improved tumor targeting efficacy, but increased clinical concerns in terms of immune responses and adverse effects.***

Response: Thanks so much for the reviewer's comments. As shown in Figure 3, with the two-step injections strategy, both efficient tumor retention and rapid RES clearance can be achieved. Furthermore, compared with non-assembling (1st + 1st, T/N = 2.5) and pre-assembly (1st + 2nd, T/N = 2.7) groups, the *in vivo* assembly strategy exhibited highest T/N ratio of 12.5 (Figs. 3f, S24-26). According to the previous work (*Nat. Nanotechnol.*, **2012**, *7*, 389-393), the immune response of nanoparticles can be monitored by measuring the interferon-α (IFN-α) levels in blood samples We have investigated the IFN-α in blood which was collected after 12 h, 24 h, 36 h PI of the 1st injection respectively. There was no significant increase in IFN-α compared to the blood samples in untreated mice (Fig. S12 in the revised supporting information), indicating immune response could be excluded for the *in vivo* bioimaging with the two-step injection strategy.

In order to make this point clearer for the readers, we have added the following sentences in the revised manuscript (on page 7, lines 140-143): "Finally, the immune response of assembled DSNPs was monitored by measuring the interferon-α (IFN-α) levels in blood samples.[43] There was no significant increase in IFN-a compared to levels in untreated mice (Fig. S12), indicating immune response could be excluded for the *in vivo* bioimaging with the two-step injection strategy."

We have added Fig. S12 in the revised supporting information as following:

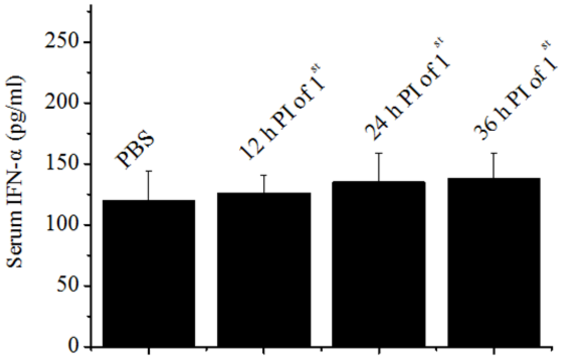

Fig. S12. Analysis of serum immune response of PBS (1X) and DSNPs after 12, 24, 36 h PI of the 1st injection. Serum IFN-α release was not detected after injection of DSNPs, indicating immune response could be excluded for the *in vivo* bioimaging with the two-step injection strategy. Error bars, mean ± s.d. (*n* = 5). Mean ± s.d. for n = 5.

8. ***Tumor uptake and retention were well quantified in this study. In many figures, tumor to normal tissue ratio was displayed, but it isn't clear what's the normal tissue used to compare. Tumor-to-liver ratio is an important indicator for tumor targeting, the comparison should be against the liver.***

Response: We accept this comment. In the subcutaneous tumor model (Fig. 3, Fig. 4 in the revised manuscript and Fig. S23, Fig. S26 in the revised supporting information), the normal tissue in our studies refers to the liver. In order to make this point clearer for readers, **we have**

added the "The T/N ratio is tumor-to-liver ratio in this epidermal tumor model experiments." in the figure captions of Figs. 3-4 in the revised manuscript and Fig. S23, Fig. S26 in the revised supporting information. In the human ovarian adenocarcinoma peritoneal metastases model (Fig. 5 in the revised manuscript and Fig. S48 in the revised supporting information) and popliteal lymph node metastasis model, since the bioimaging signals in the liver were very weak, the normal tissue refers to the normal peripheral tissue of the metastasis. In order to make this point clearer for readers, **we have added the "The T/N ratio is tumor-to-normal peripheral tissue ratio in this peritoneal metastases model and popliteal lymph node metastasis model experiments." in the figure caption of Fig. 5 and "The T/N ratio is tumor-to-normal peripheral tissue ratio in this peritoneal metastases model experiments" in the figure caption of Fig. S48 in the revised supporting information.**

9. ***Scale bars are missing in Fig. 3a,e, Fig. 4a,b, and Fig. 5a.***

Response: We accept this comment.

We have added scale bars in Fig. 3a.and Fig. 3e. We have added "All scale bars, 1cm." in the figure caption of Fig. 3.

We have added scale bars in Fig. 4a and Fig. 4b. We have added "All scale bars in NIR-II bioimaging and MRI results represent 5 mm." in the figure caption of Fig. 4.

We have added scale bars in Fig. 5a. We have added "All scale bars, 1cm." in the figure caption of Fig. 5.

10. ***Blood curve and urinary excretion of 1st injection is not displayed.***

Response: Thanks for the comment. The blood curve and urinary excretion of 1st injection are added in the revised supporting information as Fig. S15a.

We have added the following description in the revised manuscript (on page 7, lines 153-154): "Moreover, according to biodistribution results, the half-life of the nanoprobes in blood is ~ 2 h and 45.1 % ID/g is excreted from urine (Fig. S15a)."

We have added Fig. S15a in the revised supporting information as following:

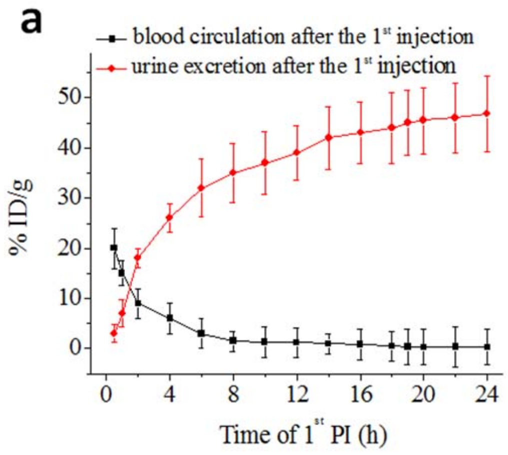

Fig. S15. (**a**) Blood circulation and urine excretion of nanoprobes after single 1st injection. Mean ±

s.d. for n = 5.

11. ***Show resected tumors with color and fluorescence images so that readers can see the correlated size and intensity of each tumor.***

Response: We appreciate this suggestion. We have added both color and fluorescence images of subcutaneous tumor model and human ovarian adenocarcinoma peritoneal metastatic model in the revised supporting information Figs. S33 and S46.

We have added the following description in the revised manuscript (on page 11, lines 228-230): "On the other hand, tumors were removed in this surgical time window (supplementary video 1, Fig. S33) and then evaluated by H & E staining."

We have added the following description in the revised manuscript (on page 11, lines 246-248): "Unexpectedly, all the large tumor boundary (No.1 - 8) and invisible small metastatic lesion (No. 9 - 13) are capable of being identified by NIR-II fluorescence bioimaging in the optimal surgical time window (Fig. 5a, S46, supplementary video 2)."

We have added Fig. S33 in the revised supporting information as following:

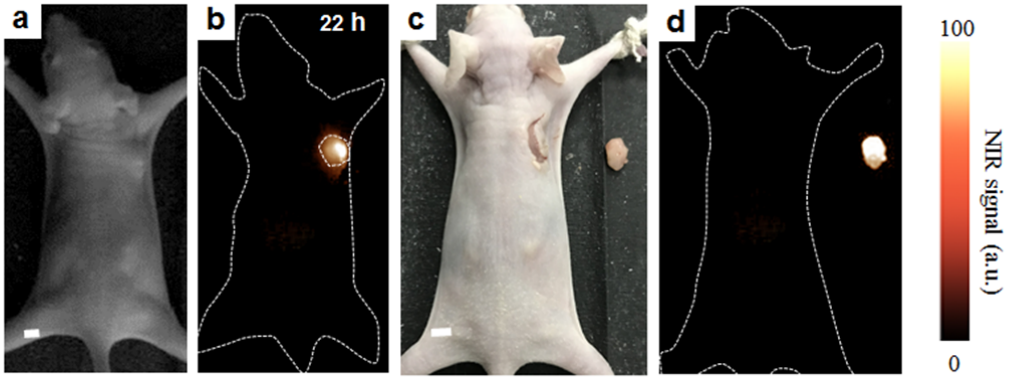

Fig. S33. NIR-II fluorescence bioimaging result (22 h PI of 1st injection) of epidermal tumor (**a**, **b**), digital image (**c**) and NIR-II fluorescence bioimaging result (d) after the surgical resection. All scale bars, 5 mm. Representative images are for n = 5.

We have added Fig. S46 in the revised supporting information as following:

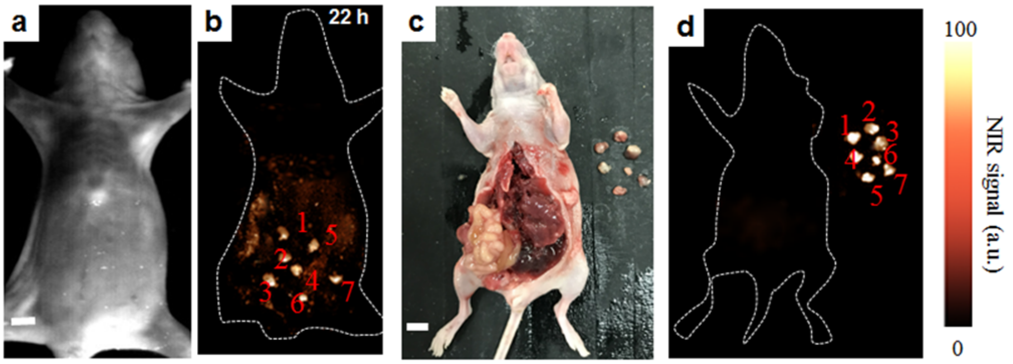

Fig. S46. The NIR-II fluorescence bioimaging result (22 h PI of 1st injection) of peritoneal metastases (**a, b**), digital image (**c**) and NIR-II fluorescence bioimaging result (**d**) after the surgical resection. All scale bars, 5 mm. Representative images are for n = 5.

12. *Histology in Fig 5c confirms the resected tumors. This should be also taken under the fluorescence microscope to confirm the NIR signals in the tumoral tissue.*

Response: Thanks so much for the comment. Since there are not commercialized confocal laser scanning microscope for the NIR-II bioimaging (900-1700 nm), we tried to confirm the nanoprobe signals in the tumoral tissue with a Cy5-Cy7 FRET experiment by using the regular confocal laser scanning microscope (CLSM) (400-800 nm). Nanoprobes grafted with Cy5 fluorescent dyes labeled DNA (DSNPs-L_1(Cy5)-FSH_β) and nanoprobes linked with Cy7 labeled complementary DNA (DSNPs-L_2(Cy7)-FSH_β) were injected into the epidermal tumor model mice and human ovarian adenocarcinoma peritoneal metastases model mice respectively for *in vivo* self-assembly by the two-stage strategy. The location of the assembled nanoprobes can be confirmed by the Förster resonance energy transfer (FRET) signals between the Cy5 and Cy7 dyes while bringing them within close proximity. As shown in Fig. S34 in the revised supporting information, the border regions of both subcutaneous tumors and peritoneal metastasis between normal and tumor tissues can be observed through the FERT signals, further indicating the efficient tumor resection of the *in vivo* assembly strategy.

In order to make this point clearer for the readers, we have added the following description in the revised manuscript (on page 11, lines 231-233): “Meanwhile, the resected tumor in 28 h PI has no borderline between the tumor and normal tissues in comparison to those operated in 20 ~ 26 h PI (Fig. 4d, S34).”

We have added Fig. S34 in the revised supporting information as following:

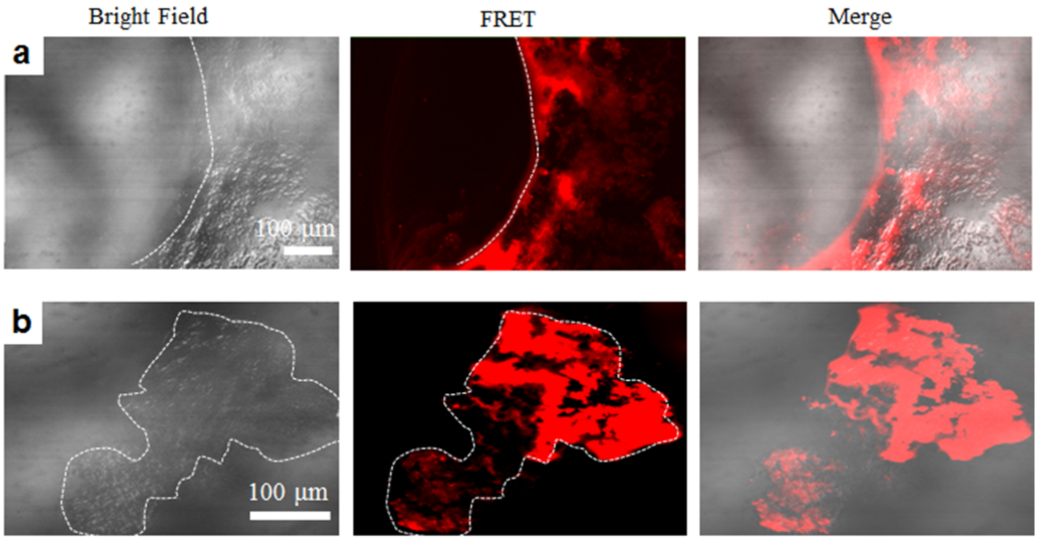

Fig. S34. Fluorescence images of the tumor frozen section (20 X). The subcutaneous tumors (**a**) and peritoneal metastasis lesion (**b**) were resected at 22 h PI of 1st injection. The FRET fluorescence in the tumor tissues can be used to verify the distribution of DSNPs. Representative images are for n = 5.

13. Also if this is really in the tumor site, it should be confirmed whether the signals come from the tumor cell or interstitial space, which will clearly support the tumor targeting concept of this study.

Response: Thanks so much for the suggestion. Similar to the above comment 12, we also used the Cy5-Cy7 FRET signals to investigate whether DSNPs assembled in tumor cell or interstitial space by using the regular confocal laser scanning microscope (CLSM) (400-800 nm). As shown in the CLSM results of tumor frozen section, FRET signals can be observed around the nuclei of tumor cells and few signals can be detected in interstitial space (Fig. S17 in the revised supporting information).

In order to make this point clearer for the readers, we have added the following description in the revised manuscript (on page 8, lines 174-176): "Meanwhile, as shown in the confocal laser scanning microscope (CLSM) results of tumor frozen section (Fig. S17), FRET signals can be observed around the nuclei of tumor cells and few signals can be detected in interstitial space."

We have added Fig. S17 in the revised supporting information as following:

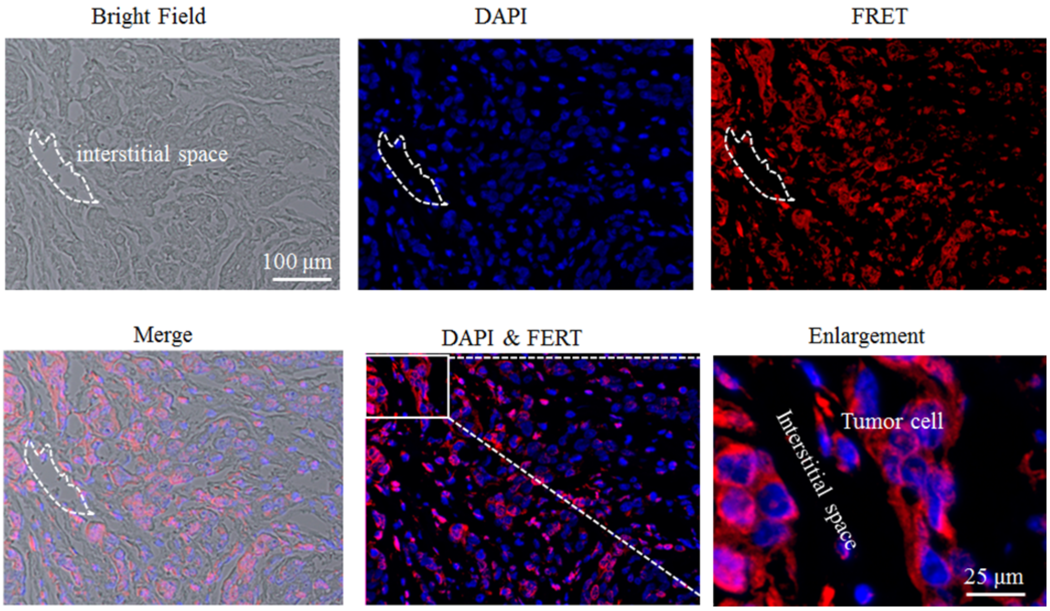

Fig. S17. Fluorescence images of the tumor frozen section (40 X). The dotted light refers to the interstitial space. The enlargement of the fluorescence image exhibited that the FRET signals came from the tumor cells. Representative images are for n = 5.

Reviewer #2 (Remarks to the Author):

In this manuscript, the authors describe near-infrared II nanoparticles for detection of disseminated ovarian cancer. The particles were constructed using layer-by-layer methodology and included an FSH targeting peptide. An important part of these particles is in vivo assembly – the authors used a two-stage injection approach whereby particles were injected at separate time points (8h being the optimum separation), with the second injection containing DNA complementary to the first, allowing assembly via Watson-Crick base-pairing. This strategy was significantly better that using preformed nanoparticles. Similarly, FSH gave better results than an integrin-binding RGD motif.

The authors performed two in vivo surgical experiments – one with a subcutaneous deposit, the other with intraperitoneal deposits, reflecting the dissemination pattern of advanced high grade serous ovarian cancer, which is the critical target patient population in ovarian cancer management.

Overall, the experiments have been performed well, with suitable controls. This reviewer is an ovarian cancer researcher rather than a polymer chemist – for the non-specialist reader, the details of the nanoparticle construction were not entirely clear, especially the difference between DSNP-L1-FSH and DSNP-L2-FSH.

Response: Thanks for these positive comments. The detailed protocols to prepare the DNA and Follicle-Stimulating Hormone ($FSH_β$) peptide modified DSNPs nanoprobes (DSNPs-L_1-$FSH_β$ and DSNPs-L_2-$FSH_β$) has been added in the Methods part of the revised manuscript (on pages 15-16,

lines 329-359) as following:

"***Coating DSPE-PEG$_{2000}$-NH$_2$ onto DSNPs*:** The oleic acid capped DSNPs (0.1 mmol) in 5 mL of chloroform was mixed with a chloroform solution (1 mL) containing 25 mg DSPE-PEG$_{2000}$-NH$_2$ in a round bottom flask. Chloroform was then removed by evaporating slowly under argon atmosphere for 24 h at room temperature. Then, the obtained mixed film was hydrated with MilliQ water (5 mL), and the obtained hydrophilic DSNPs modified with amino phospholipids could be dispersed greatly after vigorously sonication. Excess lipids were purified from amino groups modified DSNPs by centrifugation (50000 rpm, 30 min) at least 3 times. The solution was filtered with 0.22-μm filers to remove possible large aggregates.

***Coating L_1* or *L_2 onto amino modified DSNPs*:** The synthesis of DNA functionalized DSNPs was carried out according to a published ligand-exchange method[41]. Briefly, the DSPE-PEG$_{2000}$-NH$_2$ capped DSNPs (20 μmol) in 0.8 mL of aqueous solution were carefully added to a water solution (2 mL) containing 200 nmol DNA (L_1), and the solution was vigorously stirred overnight. Afterward, the solution of DSNPs could be clearer due to the L_1 attachment. Then the water solution was transferred to a microtube. After vigorous sonication for several minutes, excess L_1 was removed from DNA and amino modified DSNPs by centrifugation and washing. The large aggregates in the dispersion were removed via filtration through a 0.22 μm membrane filter. Coating L_2 onto amino modified DSNPs was carried out all the same as that of L_1 except 200 nmol L_2 were used instead of 200 nmol L_1. Finally, the two kinds of samples were dispersed in PBS (1 X).

***Fabrication of DSNPs-L_1-FSH$_\beta$ and DSNPs-L_2-FSH$_\beta$*:** Then the carboxylic acid groups on FSH$_\beta$ were activated by EDC (N-(3-Dimethylaminopropyl)-N'-ethylcarbodiimide hydrochloride) and NHS (N-Hydroxysuccinimide). In a typical procedure, 1 mg of FSH$_\beta$ was first dissolved in 5 mL 2-(N-Morpholino) ethanesulfonic acid (MES) buffer (0.1 M, pH 5), 8 mg EDC was added to above solution under stirring, then 8 mg NHS was added, the resulting mixture was stirred for 30 min at room temperature. Then 0.1 mmol of amino and L_1 functionalized DSNPs was added into the solution and stirred vigorously for 24 h. The resulting L_1 and FSH$_\beta$ modified DSNPs (DSNPs-L_1-FSH$_\beta$) were finally centrifuged at 50000 rpm, washed several times with MES buffer to remove unreacted FSH$_\beta$ followed by lyophilizing. DSNPs-L_2-FSH$_\beta$ was carried out all the same as that of L_1 except 0.1 mmol of amino and L_2 functionalized DSNPs were used instead of 0.1 mmol of amino and L_1 functionalized DSNPs. Finally, the two kinds of samples ((DSNPs-L_1-FSH$_\beta$, DSNPs-L_2-FSH$_\beta$) were dispersed in PBS (1 X)."

There are three main comments

Comments:

1. Originality. In both the introduction and the discussion, the authors make almost no mention of previous work in this field. There are several recent publications both on layer-by-layer nanoparticle construction and the use of NIR-II fluorescence specifically in ovarian cancer (e.g. see PMID 28928826, 28482280, 27114520) – these should be mentioned and compared to the data in this manuscript. Although the two-stage procedure is novel, this reviewer cannot see that this work represents a major advance in this field.

Response: We accept these comments. Dang, *et al* carries the comparison of currently available

NIR-II probes (down-conversion nanoparticles (DCNPs), quantum dots (QDs), single-walled carbon nanotubes (SWNTs), and small organic molecules) with identical layer-by-layer coatings with regard to their biodistribution, pharmacokinetics, and toxicities. Overall, rare-earth-based downshifting nanoparticles demonstrate optimal biological and optical performance and are evaluated as a diagnostic probe for high-grade serous ovarian cancer, typically diagnosed at late stage (*Proc. Natl. Acad. Sci. USA*, **2016**, *112*, 5179-184). Based on this research, in our study, we also have carried the downshifting nanoparticles with NIR-II fluorescence for ovarin tumor imaging, we made further efforts on tumor targeting and retention of nanoprobes and then utilized the NIR-II fluorescence bioimaging for efficient tumor surgery.

In order to accurately identify tiny and early tumor deposits that were otherwise missed by detection of exogenous NIR-I emissive agents, Tao, *et al* have developed a direct comparison of imaging with NIR-II vs. NIR-I signals (*Biomaterials*, **2017**, *134*, 202-215). They discovered that *in vivo* optical imaging at NIR-II wavelengths (Er^{3+} and Ho^{3+}) facilitates more accurate detection of smaller and earlier tumor deposits, offering enhanced sensitivity, improved spatial contrast, and increased depths of tissue penetration as compared to imaging with visible or NIR-I fluorescent agents. Inspired by this work, we have also explored the NIR-II fluorescence (Nd^{3+}) for eye invisible early tumor detection (Figs. S47-49) in the revised supoprting information), meanwhile, these nanoprobes have long tumor retention with T/N ratios are higher than 10.0 and H & E staining further illustrated the subsequent effective surgical resection with size $\leq$ 1 mm (Fig. S49) that all the resected fluorescent metastases were confirmed to be malignant.

For delineation of metastatic lymph nodes in ovarian cancer, Pu, *et al* have demonstrated the successful identification of metastatic retroperitoneal lymph nodes by co-localization with lymph nodes labeled by NIR-I fluorescence nanoparticles *in vivo* (*Oncol. Lett.,* **2017**, *14*, 2869-2877). In the reivesed manuscript we also investigated the NIR-II nanoprobes *in vivo* assembly for NIR-II imaged-guided lymph node metastatsis resection (Figs. 5d-e, S50-54). Long tumor retention time and higher T/N ratios (~ 10) can successfully facilitate lymphatic metastasis intra-operative identification and complete resection.

In order to make this point clearer for the readers, we have added the following sentences in the introduction part of the revised mauscript (on pages 3-4, lines 68-75):

"Second-window near-infrared fluorescence (NIR-II, 900–1700 nm) probes such as single-walled carbon nanotubes (SWNTs)[23,24], quantum dots (QDs)[25,26], lanthanide-based downshifting nanoparticles (DSNPs)[27-29] and organic dyes[30-33] are promising for *in vivo* fluorescence imaging due to sub-10-μm high-resolution imaging at a few centimeters tissue penetration depth and low tissue autofluorescence[23-33], which are promising candidates for both preoperative imaging and intraoperative reality[28]. Especially, DSNPs play a very important role in the NIR-II fluorescent bioimaging applications due to their distinct properties, such as highly controlled particle size, non-photobleaching, long lifetime and high efficiency optical properties[27-29]."

We have also added the following scentences in the disscusion part of the revised mauscript (on pages 13-14, lines 283-300):

"Thus far, NIR-II contrast agents including SWNTs[23,24], QDs[25,26], DSNPs[27-29] and organic dyes[30-33] have been established for *in vivo* imaging of vascular flow, lymphatic and tumors. Although the organic dyes shows great potentials to facilitate FDA approval and clinical

translation, these small molecule probes typically experienced short time tumor retention because of their rapid clearance, which hindered the following precision imaging guided resection. For the inorganic SWNTs, QDs and DSNPs NIR-II probes, the long term cytotoxicity is still a major concern. One way to overcome these problems is to design nanoprobes with sizes smaller than 10 nm, i.e., below what is believed to be the threshold for renal clearance[56,57]. Since the well controllable particle size and superior optical properties including non-photobleaching, long lifetime and high efficiency, DSNPs NIR-II probes have attracted a great of attentions for the *in vivo* bioimaging recently. For example, early ovarian tumor detection was explored by using lanthanide DSNPs (Er^{3+} and Ho^{3+} doped)[39,58] and even multi-organ cancer metastases could be successfully visualized (Er^{3+} doped)[59]. Unfortunately, all current in vivo bioimaging strategy for imaging-guided surgery with DSNPs NIR-II nanoprobes are still limited by the serious RES accumulation and short time tumor retention[28,29]. In the present work, we have developed a novel *in vivo* self-assembly bioimaging strategy to realize the rapid whole body clearance and long tumor retention nanoprobes and then utilized the NIR-II fluorescence bioimaging for efficient tumor surgery."

We have cited the following reference in the revised manuscript (on pages 23-24, lines 508-516, page 24, lines 533-535, page 26, lines 569-571, page 27, 582-592):

"(31) Li, B., Lu, L., Zhao, M., Lei, Z. & Zhang, F. Efficient 1064-nm NIR-II excitation fluorescent molecular dye for deep-tissue high-resolution dynamic bioimaging**.** *Angew. Chem. Int. Ed.* https://doi.org/10.1002/anie.201801226 (2018).

(32) Qi, J., Sun, C., Zebibula, A., Zhang, H., Kwok, R. T. K., Zhao, X., Xi, W., Lam, J. W. Y., Qian, J. & Tang, B. Z. Real-time and high-resolution bioimaging with bright aggregation-induced emission dots in short-wave infrared region. *Adv. Mater.* https://doi.org/10.1002/adma.201706856 (2018).

(33) Cosco, E. D., Caram, J. R., Bruns, O. T., Franke, D., Day, R. A., Farr, E. P., Bawendi, M. G. & Sletten, E. M. Flavylium polymethine fluorophores for imaging in the near- and shortwave infrared. *Angew. Chem. Int. Ed.* **56**, 13126-13129 (2017).

(39) Dang, X. N., Gu, L., Qi, J., Correa, S., Zhang, G., Belcher, A. M. & Hammond, P. T. Layer-by-layer assembled fluorescent probes in the second near-infrared window for systemic delivery and detection of ovarian cancer. *Proc. Natl. Acad. Sci. USA* **13**, 5179-5184 (2016).

(51) Pu, T., Xiong, L., Liu, Q., Zhang, M., Cai, Q., Liu, H., Sood, A. K., Li, G., Kang, Y. & Xu, C. Delineation of retroperitoneal metastatic lymph nodes in ovarian cancer with near-infrared fluorescence imaging. *Oncol. Lett.* **14**, 2869-2877 (2017).

(56) Peer, D., Karp, J. M., Hong, S., Farokhzad, O. C., Margalit, R. & Langer, R. Nanocarriers as an emerging platform for cancer therapy. *Nat. Nanotechnol.* **2**, 751-760 (2007).

(57) Ma, K., Sai, H. & Wiesner, U. Ultrasmall sub-10 nm near-infrared fluorescent mesoporous silica nanoparticles. *J. Am. Chem. Soc.* **134**, 13180-13183 (2012).

(58) Tao, Z., Dang, X., Huang, X., Muzumdar, M. D., Xu, E. S., Bardhan, N. M., Song, H., Wuckoff, J., Birrer, M. J., Belcher, A. M. & Ghoroghchian, P. P. Early tumor detection afforded by *in vivo* imaging of near-infrared II fluorescence. *Biomaterials*, **134**, 202-215 (2017).

(59) Kantanneni, H., Zevon, M., Donzanti, M. J., Zhao, X., Shen, Y., Barkund, S. R., McCabe, L. H., Petrosky, W. B., Higgins, L. M., Ganesan, S., Riman, R. E., Roth, C. M., Tan, M. C., Pierce, M. C., Ganapathy, V. & Moghe, P. V. Surveillance nanotechnology for multi-organ cancer metastases. *Nat. Biomed. Eng.* **1**, 993-1003 (2017)."

2. ***The use of a single xenograft model. Although the work presented here is about proof-of-methodology, it would be more impressive if a second model were utilised. This also raised the issue of FSH as the target peptide. FSH is not commonly assessed as an immunohistochemistry marker in high grade serous carcinoma – what proportion of these tumours express FSH at appropriate levels?***

Response: We appreciate these useful comments. We have prepared another two epidemic tumor models with ovarian tumor cell HO8910 and A2780 respectively. Then all the tumors were applied for the *in vivo* assembly strategy. NIR-II fluorescent bioimaging results exhibited that both HO8910 and A2780 cell lines epidermal tumors can be successfully identified in the optimal surgical time window. H & E staining were studied after tumors were removed under NIR-II fluorescence bioimaging, which confirmed the precise delineation of the tumor margin (Figs.40-41 in the revised supporting information). The liver localization of nanoprobes in these two ovarian epidermal tumors was analyzed by inductively coupled plasma mass spectrometer (ICP-MS). The results exhibited that HO8910 and A2780 cell lines epidermal tumor models have the similar liver retention with that of $CaOV_3$ cell line, suggesting that nanoprobes also experienced rapid whole-body elimination in these two tumor models (Fig. S42a in the revised supporting information).

In our ovarian tumor model, we utilized ovarian tumor cell line $CaOV_3$ which has highest the follicle-stimulating hormones receptor (FHSR) expression levels compared to other ovarian cancer cell lines, like OVCAR-3 and $SKOV_3$ cells (*Cancer Res.*, **2009**, *69* (16), 6506-6514). We have also conducted FSHR expression in the cell lines of $CaOV_3$, HO8910 and A2780 by immunohistochemical (IHC), wester-bloting (WB) and real time quantitative polymerase chain reaction (qPCR) methods to analyze the mRNA expression of FSHR. IHC, WB and qPCR results demonstrated that FSHR expression are $CaOV_3$ > HO8910 > A2780 (Fig. S2 in the revised supporting information). Meanwhile, tumor targeting efficiencies of nanoprobes for HO8910 and A2780 cell lines tumor model are less than that of $CaOV_3$ (Fig.42b in the revised supporting information). These results are well coincided with tumor targeting data of nanoprobes in the revised manuscript as Fig. S2.

In order to make this point clearer for the readers, we have added the following sentences in the revised manuscript (on page 11, lines 240-243): "Furthermore, other two ovarian epidermal tumor models (HO8910 and A2780 cell lines) with lower FSH_β receptor (FSHR) expression levels can also be observed and thoroughly removed, suggesting the universality of our approach for ovarian tumor surgery (Figs. S2, S40-42)."

We have added Figs. S40-42 in the revised supporting information as following:

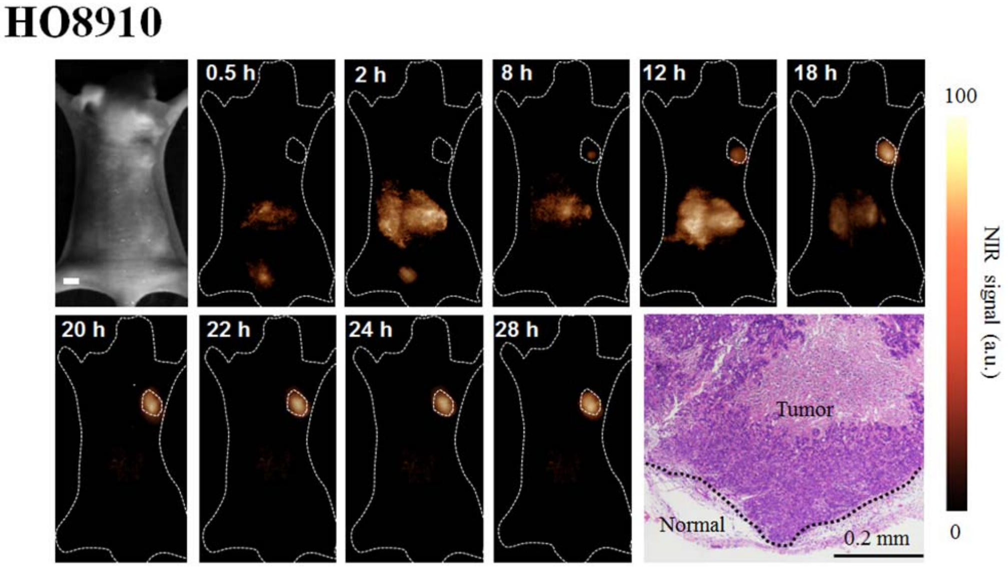

Fig. S40. NIR-II fluorescence image-guided surgery for HO8910 epidermal ovarian tumor model (21 days after HO8910 cells subcutaneous injection). The resection was operated in the optimal surgical time window (22 h PI of 1st injection,). Tumor margin was confirmed by the H & E staining result. Scale bar represents 5 mm for the NIR-II fluorescence bioimaging results. Representative images are for n = 5.

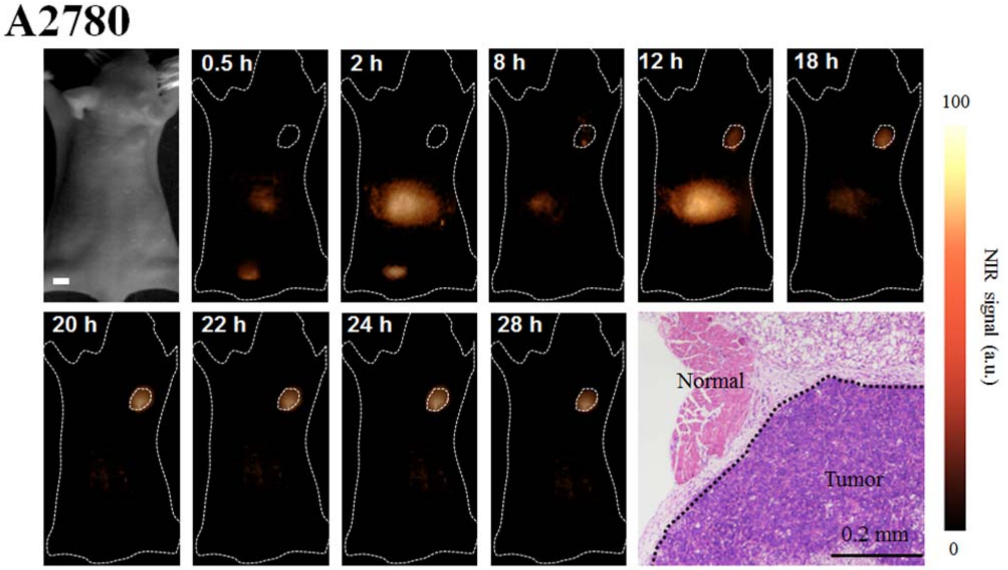

Fig. S41. NIR-II fluorescence image-guided surgery for A2780 epidermal ovarian tumor model (21 days after A2780 cells subcutaneous injection). The resection was operated in the optimal surgical time window (22 h PI of 1st injection,). Tumor margin was confirmed by the H & E staining result. Scale bar represents 5 mm for the NIR-II fluorescence bioimaging results. Representative images are for n = 5.

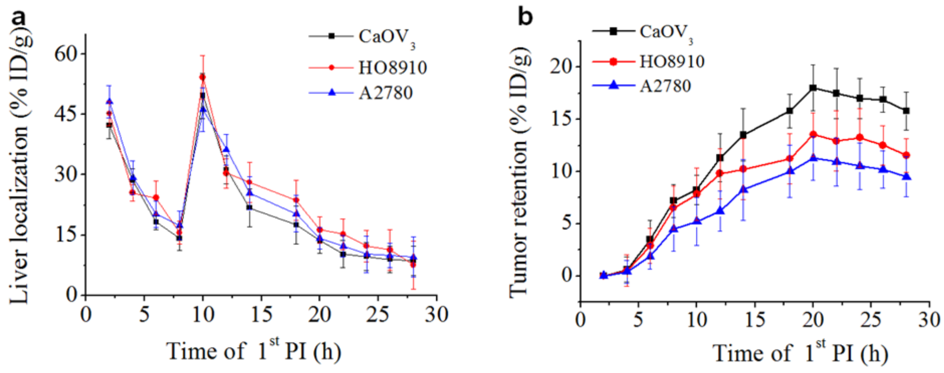

Fig. S42. Liver localization (**a**) and tumor retention (**b**) of nanorprobes in our epidermal tumor model ($CaOV_3$ cell line) and other two kinds of epidermal ovarian tumors models (HO8910 and A2780 cell lines) which were analyzed by ICP-MS. The 2nd injection of HO8910 and A2780 epidermal ovarian tumors-bearing mice were administrated by tail injection at 8 h PI of 1st injection which was the same as the $CaOV_3$ epidermal tumor. Mean ± s.d. for n = 5.

We have added Fig. S2 in the revised supporting information as following:

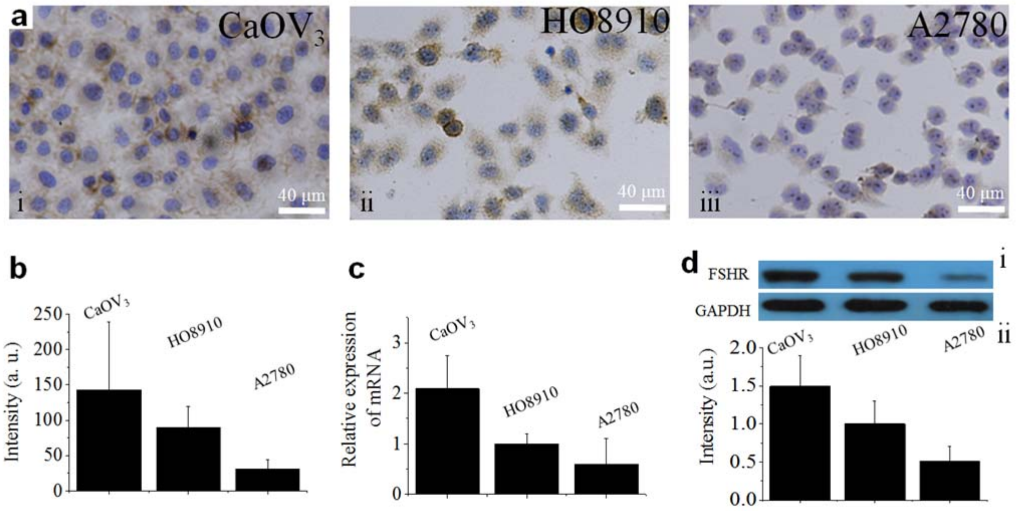

Fig. S2. (**a**) Expression of FSHR in the ovarian cancer cell lines $CaOV_3$ (i), HO8910 (ii) and A2780 (iii) as estimated by immunohistochemical (IHC) method. (**b**) The statistical analysis of the IHC results. (**c**) mRNA relative expression of FSHR in three different cell lines. (**d**) FSHR expression in three different cell lines measured by WB method. (i) Expression of FSHR protein in three different cell lines and GAPDH (glyceraldehyde-3-phosphate dehydrogenase) is used as loading control. Statistical analysis of WB results (ii). Representative images are for n = 5 per group. Mean ± s.d. for n = 5.

3. ***Downstream application. Is advanced ovarian cancer the correct setting in which to apply this technology? Experienced ovarian cancer surgeons can already remove all visible disease***

through extensive surgical effort (stripping diaphragmatic peritoneum etc) – and yet 80+% of women with advanced high grade serous carcinoma still relapse. The recent LION study indicated that the additional resection of macroscopically normal nodes in addition to all visible macroscopic disease made no impact upon progression/recurrence. Thus, I am struggling somewhat to see how this technology might be used in ovarian cancer. Other uses (e.g. detection of sentinel LN, resection margins within the liver or brain) might be more immediately useful.

Response: Thanks for the useful suggestions. We totally agree with the reviewer's point. As the reviewer mentioned, the recent LION study indicated that the additional resection of macroscopically normal nodes in addition to all visible macroscopic disease made no impact upon progression/recurrence. We speculate the recurrence might be owing to the ultra-small eye-invisible cancerous lesions are difficult to be removed thoroughly during the surgery. In the present work, since ≤ 1 mm metastatic lesions can be thoroughly removed, this novel tumor targeting strategy might be able to avoid the recurrence by correctly identifying eye-invisible cancerous metastases. We will further investigate this meaningful work in the future.

In order to make this point clearer for the readers, we have added the following sentences in the Discussion part of revised manuscript (on page 14, lines 309-315): "Experienced ovarian cancer surgeons can already remove all visible disease through extensive surgical effort, and yet over 80 % of women with advanced high grade serous carcinoma still relapse. The recent clinical study indicated that the additional resection of macroscopically normal nodes in addition to all visible macroscopic disease made no impact upon progression/recurrence. In the present work, since ≤ 1 mm metastatic lesions can be thoroughly removed, this novel tumor targeting strategy might be able to avoid the recurrence by correctly identifying eye-invisible cancerous metastases."

We really appreciate the reviewer's suggestion about the lymph node metastasis model detection. We have set up a popliteal lymph node metastasis model by intradermal injected $CaOV_3$ cell (5 *10^8 cells/mouse) to the hind paw of five-week-old mice. Figs. S50-S51 displayed that this model was successful built up after 21 days. Then we have studied the potential application of intraoperative imaging technology for popliteal lymph node metastasis model. Interestingly, compared with the normal lymph node, lymph node metastasis can be successfully delineated by NIR-II fluorescence bioimaging in the optimal surgical time window (Fig. 5d in the revised manuscript and Fig. S52 in the revised supporting information). Furthermore, NIR-II fluorescence imaging results of lymph node metastasis mice with intradermal hind paw injection were also investigated. Moreover, the lymph node metastasis cannot be observed by traditional intradermal hind paw injection in normal lymph node (*Adv. Funct. Mater.* **2017**, *27*, 1700995) (Fig. S53 in the revised supporting information), further suggesting the advantage of the *in vivo* assembly with two-staged intravenous injection. T/N ratios with *in vivo* assembly strategy are still kept on ~ 10 similar to that of the epidermal tumor in the optimal surgical time window (Fig. 5e in the revised manuscript). H & E staining illustrated the subsequent effective surgical resection of this macroscopic lymph node metastasis lesion (Fig. 5f in the revised manuscript and Fig. S54 in the revised supporting information).

In order to make this point clearer for the readers, we have added the following sentences in the revised manuscript (on page 17, lines 373-375): "**Popliteal lymph node metastasis model**: Five-week-old mice were inoculated in the left hind paw with $CaOV^3$ cells (5 *10^8

cells/mouse). After 21 days intradermal injection, these tumors cells spontaneously formed lymph node metastasis in the popliteal lymph nodes."

We have added the following sentences in the revised manuscript (on page 12, lines 256-259): "Furthermore, for additional resection of lymph node metastasis[51], the *in vivo* assembly strategy can further facilitate popliteal lymph node metastasis detection (Figs.5d-e and S50-54). H & E staining result demonstrated that microscopic lymph node metastasis can be successfully removed (Fig. 5f)."

We have cited the following reference in the reivsed manuscript (page 26, lines 569-571): " (51) Pu, T., Xiong, L., Liu, Q., Zhang, M., Cai, Q., Liu, H., Sood, A. K., Li, G., Kang, Y. & Xu, C. Delineation of retroperitoneal metastatic lymph nodes in ovarian cancer with near-infrared fluorescence imaging. *Oncol. Lett.* **14**, 2869-2877 (2017)."

We have added Figs. S50-S54 in the revised supporting information as following:

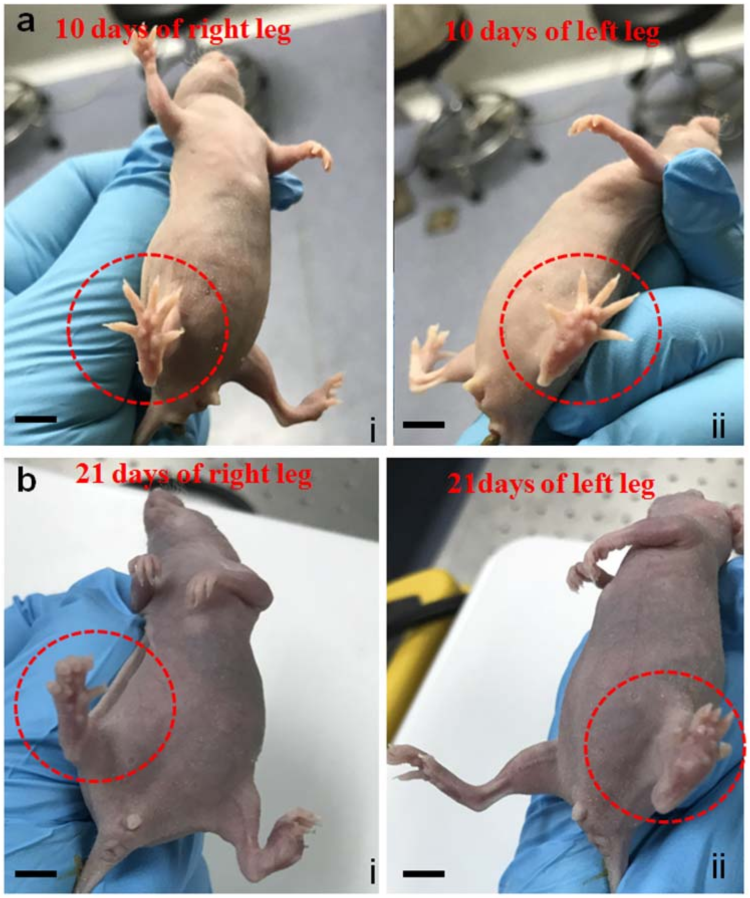

Fig. S50. A popliteal lymph node metastasis model by intradermal injected $CaOV_3$ cell (5 $*10^8$ cells/mouse) to the hind paw of five-week-old mice for 3 weeks. Digital images of mice with normal right legs (**ai**, **bi**) and left legs with intradermal hind paw injection of $CaOV_3$ cells after 10 days (**aii**) and 21 days (**bii**). Scale bars, 1 cm.

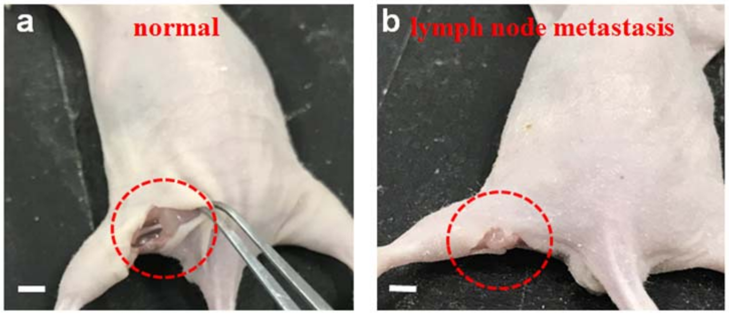

Fig. S51. Digital images of normal (**a**) and popliteal lymph node metastasis-bearing mice (**b**). Scale bars, 5 mm.

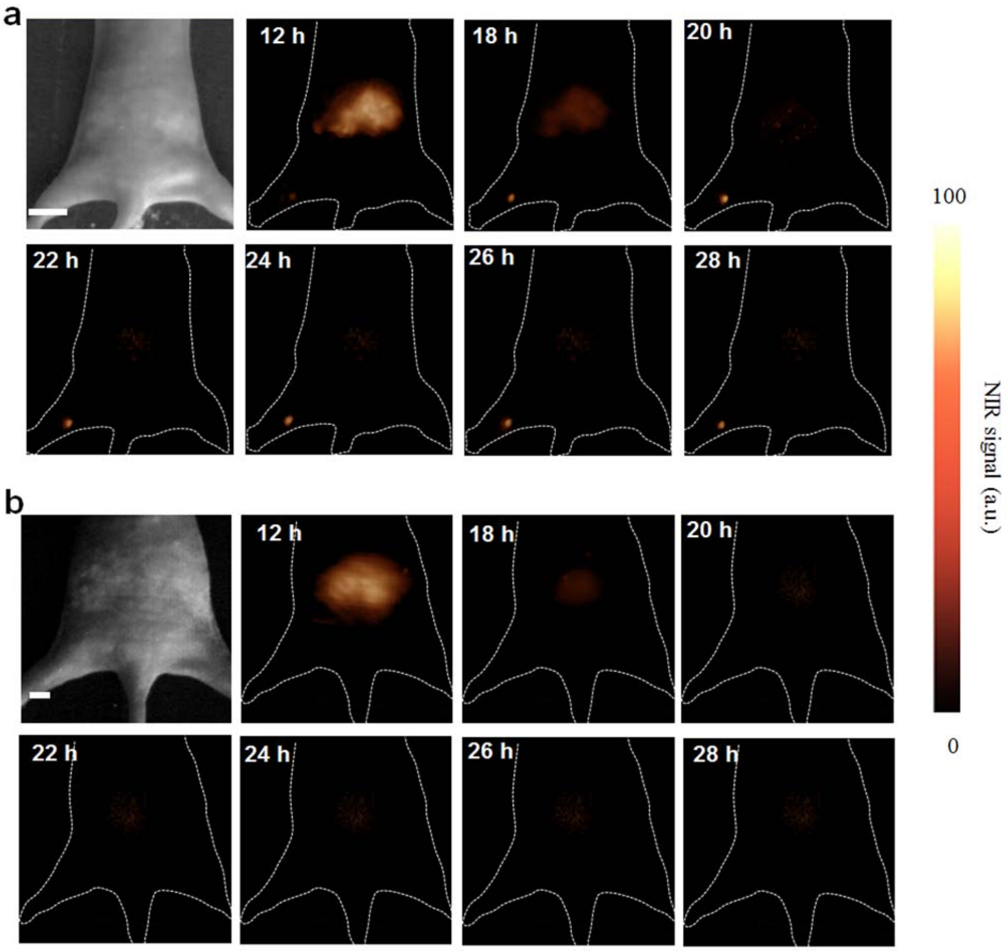

Fig. S52. NIR-II fluorescence bioimaging of popliteal lymph node metastasis model (**a**) and normal popliteal lymph node (**b**). The 2nd injection was administrated by tail injection at 8 h PI of 1st injection which was the same as the murine epidermal tumor. Scale bars, 5 mm. Compared with the normal lymph node, lymph node metastasis can be successfully delineated by NIR-II fluorescence bioimaging in the optimal surgical time window. Representative images are for n = 5 per group.

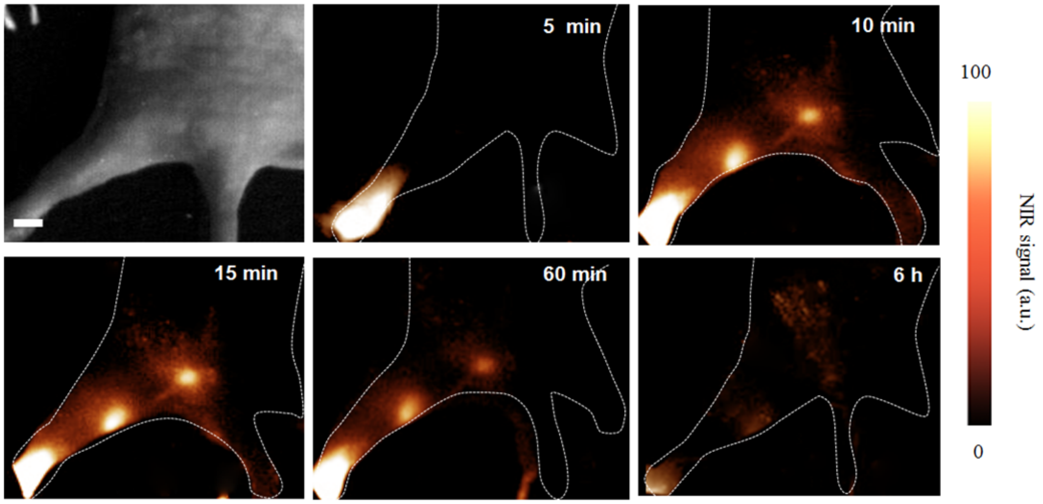

Fig. S53. NIR-II fluorescence bioimaging of normal lymph node after intradermal administrated the 1st injection at the dorsal skin of the left hind paw. The popliteal lymph node metastasis cannot be observed by traditional intradermal hind paw injection in popliteal lymph node metastasis model. Scale bar, 5 mm. Representative images are for n = 5.

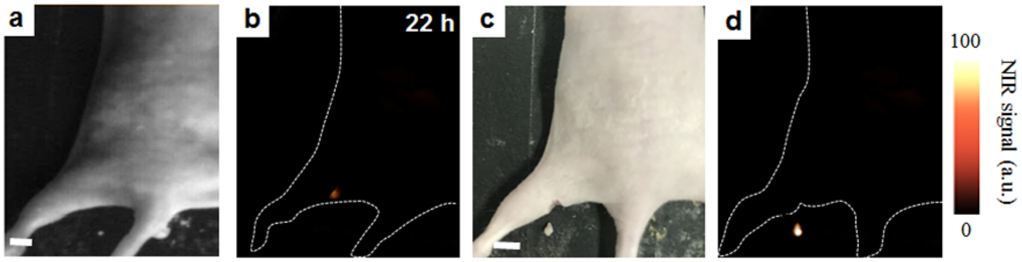

Fig. S54. The NIR II bioimaging results (22 h PI of 1st injection) of popliteal lymph node metastasis (**a**, **b**). digital image (**c**) and NIR-II fluorescence bioimaging result (d) after surgical resection. All scale bars, 5 mm. Representative images are for n = 3.

We have added Figs. 5d-f in the manuscript as following:

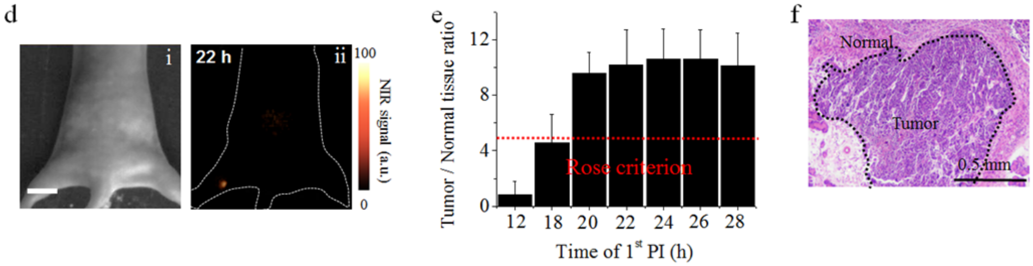

Fig. 5. | NIR-II image-guided surgical resection surgery. (**d**) NIR-II fluorescence bioimaging results of the popliteal lymph node metastasis at 22 h PI. (**e**) T/N ratios plotted as a function of different PI of the 1st injection, red dotted line is according to the Rose criterion. (**f**) H & E staining results of popliteal lymph node metastasis. Tumors were resected under NIR-II

fluorescence bioimaging guidance in d. All scale bars, 1cm. The T/N ratio is tumor-to-normal peripheral tissue ratio in this peritoneal metastases model and popliteal lymph node metastasis model experiments. Representative images are for n = 5 per model. Mean ± s.d. for n = 5.

4. ***Line 107. "5 %Nd@NaGdF4" - what does this actually mean?***

Response: $NaGdF_4$: 5 %Nd@$NaGdF_4$ refers to the core-shell structure of our down-shifting nanoparticles. This inorganic down-shifiting fluorescence core consists of a crystalline host ($NaGdF_4$) and a dopant (Nd^{3+}) added in a low concentration (5 %). The dopant provides luminescent centers, and the host lattice with its crystal structure provides a matrix to realize efficient fluorescent emission for bioimaging detection. The shell ($NaGdF_4$) with small lattice mismatch around the core to reduce non-radiative decay losses of the surface luminescence can provide an effective way to improve the optical properties of DSNPs (*Chem. Soc. Rev.*, **2015**, *44*, 1346-1378).

5. ***Figure 1 image: "Time interval with 8h" should be "Time interval of 8h".***

Response: We have replaced "Time interval with 8 h" with "Time interval of 8 h" in Fig.1 in the revised manuscript.

6. ***Fig. 2f – was this experiment conducted at 37 °C?***

Response: We have investigated the optical intensity of DSNPs in water and different psychological buffers (PBS, serum, blood) under 37 °C. **We have added this results** in the revised manuscript as Fig. 2f.

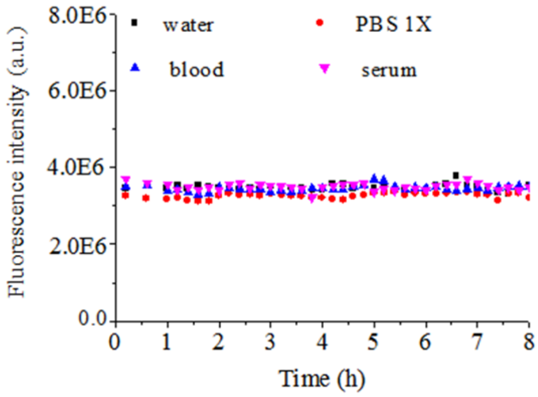

Fig. 2f Photostability of DSNPs in a variety of biological buffers at 37 °C under continuous 808 nm laser exposure at a power density of 0.2 W/cm^2.

7. ***Fig. S5 – I think that there is a mistake – think the red figure legend label should be DSNPs-L_1-FSH + DSNPs-L_2-FSH.***

Response: We have corrected the following mistake in the revised supporting information

(on page 14, lines 290-293): “DSNPs-L_1- $FSH_β$” was changed to “DSNPs-L_2- $FSH_β$”.

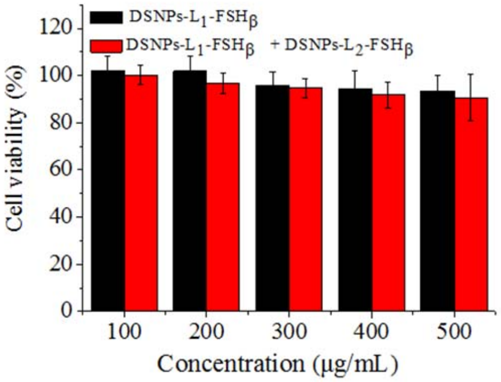

Fig. S10. Cell viabilities of DSNPs and intracellular assembled DSNPs. Mean ± s.d. for n = 5.

8. ***Fig. S6 – no error bars. How many mice were these experiments performed in? Please add error bars and state N in legend.***

Response: We have added error bars and “Mean ± s.d. for n = 5.” in the figure caption of Fig. S11 in the revised supporting information.

9. ***Lines 144 and 150 – the word ‘drastically’ is a little dramatic – ‘greatly’, ‘markedly’ or even ‘significantly’ are more appropriate.***

Response: We have corrected “drastically” to “markedly” in the revised manuscript on page 7, line 150 and line 156.

REVIEWERS' COMMENTS:

Reviewer #1 (Remarks to the Author):

The authors have made a concerted effort to address all the criticisms and the manuscript is substantially improved. The manuscript should be acceptable for publication in Nature Communications.

Reviewer #2 (Remarks to the Author):

The authors have made significant alterations to the manuscript in response to the reviewers' comments. The manuscript is improved as a result.

There are two comments that arise as a result of the revisions, both of them in response to reviewer 1 comments
1. Tumor-to-normal ratios. The authors have clarified that T/N ratios largely use liver as the normal. This is of course entirely acceptable. However, Figure S46 raised the important issue of background fluorescence in the gut – there appears to be a large quantity of fluorescence in the gastrointestinal tract in the intact mouse. This has disappeared in Figure S46D – was this due to tumor (in which case it should be shown in S46D) or gut? If the latter, then perhaps T/N ratios should be calculated relative to the normal tissue of immediate relevance for that tumor.
2. Immune responses. I acknowledge that a previous publication in Nat. Biotechnology suggests that IFN-alpha responses can be used to monitor immune response to nanoparticles. However, the immune system is somewhat more complicated than very acute interferon responses. The adaptive responses to nanoparticles (neutralising Ab in particular) could arise without significant IFN-alpha responses. Thus, I think that the sentence in which it is stated that the absence of IFN responses indicates that "immune response could be excluded for the in vivo bioimaging with the two-step injection strategy" is over-simplistic and should be removed or at least modified.

Two minor alterations.
Line 214 – 'metastatic' not 'metastasis'
Line 237 – 'worth mentioning' not 'worth to mention'

Point-by-Point Response to Referees
Reviewers' comments:

Reviewer #1 (Remarks to the Author):

The authors have made a concerted effort to address all the criticisms and the manuscript is substantially improved. The manuscript should be acceptable for publication in Nature Communications.

Response: We appreciate the reviewer for the positive comment.

Reviewer #2 (Remarks to the Author):

The authors have made significant alterations to the manuscript in response to the reviewers' comments. The manuscript is improved as a result.

There are two comments that arise as a result of the revisions, both of them in response to reviewer 1 comments

1. Tumor-to-normal ratios. The authors have clarified that T/N ratios largely use liver as the normal. This is of course entirely acceptable. However, Figure S46 raised the important issue of background fluorescence in the gut – there appears to be a large quantity of fluorescence in the gastrointestinal tract in the intact mouse. This has disappeared in Figure S46D – was this due to tumor (in which case it should be shown in S46D) or gut? If the latter, then perhaps T/N ratios should be calculated relative to the normal tissue of immediate relevance for that tumor.

Response: Thanks for the comments. Strong NIR-II fluorescence were observed in all resected peritoneal ovarian metastases, which result in the weak background signals in the gastrointestinal tract. In order to make this point clearer for the readers, we have also investigated the NIR-II fluorescence bioimage-guided metastatic surgery in another peritoneal ovarian metastases-bearing mouse. When the fluence rate is decreased from original 40 mW cm^{-2} to 25 mW cm^{-2}, the background signals can be observed (Supplementary Fig. 45 in the revised supporting information). Since the bioimaging signals in the liver were very weak, in the human ovarian adenocarcinoma peritoneal metastases model (Fig. 5 in the revised manuscript and Supplementary Fig. S47 in the revised supporting information) and popliteal lymph node metastasis model, the normal tissue refers to the normal peripheral tissue of the metastases.

We have added the "The T/N ratio is tumor-to-normal peripheral tissue ratio in this peritoneal metastases model and popliteal lymph node metastasis model experiments." **in the figure caption of Fig. 5 in the revised manuscript and** "The T/N ratio is tumor-to-normal peripheral tissue ratio in this peritoneal metastases model experiments" **in the figure caption of Supplementary Fig. 47 in the revised supporting information.**

We have added Supplementary Figure 45 in the revised supporting information as following:

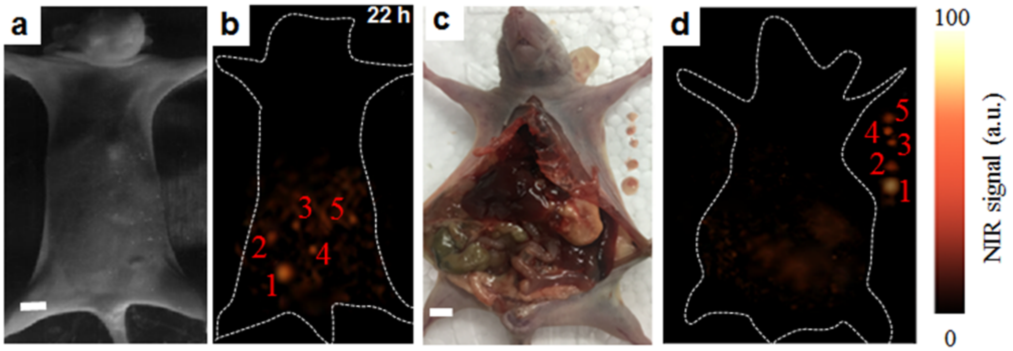

Supplementary Figure 45. The NIR-II fluorescence bioimaging result (22 h PI of 1st injection) of peritoneal metastases (**a**, **b**), digital image (**c**) and NIR-II fluorescence bioimaging result (**d**) after the surgical resection. Fluence rates, b = 40 mW cm^{-2} and d = 25 mW cm^{-2}. All scale bars, 5 mm. Representative images are for n = 5.

2. Immune responses. I acknowledge that a previous publication in Nat. Biotechnology suggests that IFN-alpha responses can be used to monitor immune response to nanoparticles. However, the immune system is somewhat more complicated than very acute interferon responses. The adaptive responses to nanoparticles (neutralising Ab in particular) could arise without significant IFN-alpha responses. Thus, I think that the sentence in which it is stated that the absence of IFN responses indicates that "immune response could be excluded for the in vivo bioimaging with the two-step injection strategy" is over-simplistic and should be removed or at least modified.

Response: Thanks so much for the useful comments and suggestions. We have removed the IFN-α results in the revised supporting information and the corresponding descriptions in the revised manuscript.

3. Line 214 – 'metastatic' not 'metastasis'

Response: We have corrected "metastasis" to "metastatic" in page 10, line 217.

4. Line 237 – 'worth mentioning' not 'worth to mention'

Response: **We have corrected** "worth to mention" to "worth mentioning" in page 11, line 241.
